# Unlocking the potential of stem cell-derived extracellular vesicles in osteoporosis therapy: a systematic review and meta-analysis of preclinical studies

**DOI:** 10.1186/s12967-025-06654-5

**Published:** 2025-06-18

**Authors:** Feng Shuang, Wen Wen, Yanjing You, Ying Zhang, Hao Li

**Affiliations:** 1 Department of Orthopedics, The 908th Hospital of Chinese People’s Liberation Army Joint Logistics Support Force, No.1028 Jinggangshan Street, Qingyunpu District, Nanchang, 330002 Jiangxi Province China; 2 Department of Respiratory and Critical Care Medicine, Fuzong Clinical Medical College of Fujian Medical University, Dongfang Hospital of Xiamen University, The 900th Hospital of Chinese People’s Liberation Army Joint Logistics Support Force, Fuzhou, 350000 Fujian Province China; 3 Department of Orthopedics, The 920th Hospital of Chinese People’s Liberation Army Joint Logistics Support Force, Kunming, 650032 Yunnan Province China

**Keywords:** Extracellular vesicle, Osteoporosis, Stem cell, Bone mineral density, Animal models, Meta-analysis

## Abstract

**Objective:**

In recent years, stem cell-derived extracellular vesicles (SC-EVs) have garnered widespread attention for the treatment of osteoporosis. Based on all available data, we conducted a comprehensive evaluation of the efficacy of SC-EVs in preclinical studies, aiming to provide the latest evidence to support their clinical translation.

**Methods:**

A comprehensive search was conducted in PubMed, Cochrane, Embase, and Web of Science databases for preclinical studies on SC-EVs for the treatment of osteoporosis, with the search period ending on November 10, 2024. Data extraction focused on three main aspects: general bone analysis parameters, histological quantification, and serum bone turnover markers. Additionally, the reporting quality and risk of bias of the studies were rigorously assessed.

**Results:**

A total of 21 studies met the inclusion criteria and were included in the final meta-analysis. The results showed that SC-EVs treatment, compared to placebo, significantly increased bone mineral density, bone volume fraction, trabecular number, and trabecular thickness, while reducing trabecular separation. Furthermore, SC-EVs treatment promoted an increase in osteoblast numbers, inhibited osteoclast numbers, and enhanced bone mineralization. Despite the presence of heterogeneity and publication bias, the results were relatively robust.

**Conclusions:**

Compared with placebo, SC-EV treatment increased bone mass and strength, improved bone microarchitecture, and enhanced biomechanical properties. These effects may be associated with the regulation of bone homeostasis through osteoblasts and osteoclasts within trabecular bone. In summary, SC-EVs demonstrate great potential in regulating bone homeostasis in osteoporosis. However, rigorous and standardized quality control in future studies is essential to facilitate the clinical translation of SC-EVs.

**Supplementary Information:**

The online version contains supplementary material available at 10.1186/s12967-025-06654-5.

## Introduction

Osteoporosis is the most common systemic metabolic disease worldwide, characterized by increased bone fragility, higher fracture risk, trabecular bone loss, and destruction of bone microarchitecture [1, 2]. It is widely recognized that bone metabolism imbalance is the primary cause of this disease, commonly seen in postmenopausal women and the elderly. With the intensification of global population aging, the risk and prevalence of osteoporosis are increasing annually. In the United States alone, approximately 1.9 million fractures per year are associated with osteoporosis, with an estimated annual healthcare expenditure of around $57 billion [3]. Complications related to osteoporosis cause immense suffering to patients, significantly reducing their quality of life and increasing rates of disability and mortality. The resulting disabilities and deaths impose a substantial burden on families and societies worldwide [4, 5]. 

Anti-osteoporosis drugs aim to reduce fracture risk by either increasing bone formation through osteoblast activity or decreasing bone resorption by inhibiting osteoclast activity. In clinical practice, the most commonly recommended bone resorption inhibitors include estrogen, calcitonin, and bisphosphonates [6]. However, studies have shown that long-term use of bone resorption inhibitors not only yields suboptimal efficacy but also leads to severe adverse effects, such as esophageal disorders and muscle pain [7, 8]. Similarly, bone formation-promoting drugs (e.g., abaloparatide, teriparatide) also face challenges of significant side effects and limited efficacy [9, 10]. For fractures caused by osteoporosis, surgical procedures like vertebral augmentation can repair fractures and relieve localized pain but may be accompanied by adverse events such as infection, embolism, and hematoma [11]. Therefore, there is an urgent need to explore novel alternative treatment options. 

In recent years, emerging therapies for osteoporosis have garnered significant attention. Stem cells, with their multilineage differentiation potential, hold promise for promoting tissue repair and regeneration, offering potential for treating osteoporosis [12, 13]. However, unresolved safety and ethical concerns, such as risks of thrombosis and tumorigenesis, remain a barrier to their clinical application [14, 15]. In contrast, stem cell-derived extracellular vesicles (SC-EVs) not only retain similar biological functions to stem cells but also present notable advantages, including low immunogenicity and excellent biocompatibility [16]. Additionally, SC-EVs enable allogeneic therapy and provide stable signaling capabilities. They facilitate intercellular communication through their molecular cargo, modulating the biological activity of recipient cells [17–19]. Studies have shown that SC-EVs promote tissue regeneration by regulating intercellular signal transduction [20], including in cases of spinal cord injury [21], skin wound healing [22], osteochondral damage [23], osteoarthritis [24], and primary ovarian insufficiency [25]. In the bone microenvironment, SC-EVs can restore cellular function and maintain homeostasis, thereby promoting bone repair and regeneration through various signaling pathways [26]. Over the past 5 years, an increasing number of preclinical studies have investigated the efficacy of SC-EVs in treating osteoporosis. 

Therefore, this systematic review and meta-analysis aims to explore the efficacy of SC-EVs in treating osteoporotic animal models while assessing publication bias and heterogeneity in the study results. The findings are intended to provide theoretical support for further research and clinical translation of SC-EVs.

## Materials and methods

### Systematic review

The study protocol was pre-defined in accordance with the Preferred Reporting Items for Systematic Reviews and Meta-Analyses (PRISMA) 2020 guidelines [27]. It was registered in the Prospective Register of Systematic Reviews (PROSPERO) (ID: CRD42020159). 

### Literature search strategy

Three authors independently conducted a comprehensive search for relevant studies across four publicly available databases: PubMed, Cochrane, Embase, and Web of Science. The search was completed with a cutoff date of November 10, 2024. Both manual and independent searches were performed using Medical Subject Headings (MeSH) terms and their free-text equivalents, with “Extracellular Vesicles” and “Osteoporosis” as the primary MeSH terms. Detailed search strategies for each database are provided in Supplementary File 1.

### Study selection criteria

Inclusion criteria:

1. Only the in vivo components of preclinical studies were included, without restrictions on species (e.g., rats, mice), sex, age, body weight, or modeling methods;
2. Experimental groups must have received treatment with SC-EVs and been designed as controlled experiments. Variations in injection methods, dosage, and administration frequency were not considered exclusionary;
3. Studies were required to report at least one of the following five outcome measures: A. Bone mineral density (BMD); B. Bone volume/total volume (BV/TV); C. Trabecular number (Tb. N); D. Trabecular separation/marrow thickness (Tb. Sp); E. Trabecular thickness (Tb. Th).
4. All included studies were based on mammalian models.

Exclusion criteria:

Phase 1 (Title and abstract screening).

1. In vitro studies only;
2. Reviews, meta-analysis, letters, and comments;
3. Non-osteoporosis animal models;
4. Publications not written in English;
5. Studies unrelated to the application of SC-EVs in the treatment of osteoporosis.

Phase 2 (Full-text review).

1. Studies involving non-SC-EVs;
2. Non-controlled experiments;
3. Studies lacking outcome measures to assess the efficacy of SC-EVs in osteoporosis treatment;
4. Studies with unreliable experimental results.

### Study selection and data extraction

Two independent authors conducted a comprehensive screening of the retrieved literature in parallel. Discrepancies were resolved through discussion with a third author. In the first phase, duplicate records were removed, and initial screening was performed based on titles and abstracts, focusing on study type, target disease, and animal models. In the second phase, full-text articles were reviewed in detail, and studies were selected according to the predefined inclusion and exclusion criteria. Reasons for exclusion were systematically documented. Additionally, the reference lists of relevant review articles were manually searched to identify any potentially eligible studies not captured in the initial database search.

Next, A standardized data extraction form was developed, and two independent authors conducted full-text reviews and extracted data simultaneously. The extracted data encompassed four main categories: general study information, animal characteristics, SC-EV characteristics, and outcome measures. General information included the year of publication, authors, and country. Animal characteristics included species, gender, age, body weight, sample size, and modeling methods. SC-EVs characteristics included their source, diameter, synthesis method, administration route, engineering target, dosing schedule, and treatment duration. Outcome measures primarily included quantitative bone parameters (BMD, BV, BV/TV, Ct.Th, Tb.BV/TV, Tb.N, Tb.Sp, Tb.Th, and femur ultimate load), hematological markers (CTX1 and OCN), and histological indicators (mineral apposition rate (MAR), number of osteoblasts, and number of osteoclasts). When statistical data were presented graphically, values were extracted using Origin software (version 2021) and reported as mean ± standard deviation (mean ± SD). Any discrepancies or data variations exceeding 10% were discussed and resolved with a third author. For studies where graphical data could not be accurately extracted, efforts were made to contact the corresponding authors to obtain the original data.

### Primary and secondary outcomes

Primary outcomes included quantitative bone parameters (BMD, BV/TV, Tb.N, Tb.Sp, and Tb.Th), all of which were reported in 15 or more studies. Secondary outcomes included BV, Ct.Th, Tb.BV/TV, femur ultimate load, hematological markers (CTX1 and OCN), and histological indicators (MAR, number of osteoblasts, and number of osteoclasts).

### Risk of bas assessment

The SYRCLE’s risk of bias assessment [28] was used to evaluate the quality of included animal studies. This tool consists of 10 items: sequence generation (selection bias), baseline characteristics (selection bias), allocation concealment (selection bias), random housing (performance bias), blinding (performance bias), random outcome assessment (detection bias), blinding of outcome assessment (detection bias), incomplete outcome data (attrition bias), selective outcome reporting (reporting bias), and other sources of bias. Two independent researchers conducted the evaluations, and any discrepancies in the results were resolved through discussion with a third researcher.

### Statistical analysis

Origin 2021, Review Manager (RevMan) 5.4, and Stata 17 were employed respectively for extracting graphical data, performing meta-analysis (including forest plots, subgroup analyses, and publication bias evaluation), and conducting sensitivity analyses. All quantitative data, whether reported directly in the studies or extracted from graphs, were recorded as mean ± SD and analyzed as standardized mean differences (SMDs) with corresponding 95% confidence intervals (CIs).

Heterogeneity across studies was evaluated using the I^2^ statistic and the χ^2^ test, following the Cochrane Handbook guidelines. Heterogeneity was classified as follows: 0–40% was considered low heterogeneity, 30–60% moderate heterogeneity, 50–90% substantial heterogeneity, and 75%−100% considerable heterogeneity. Specifically, if *I*^2^ ≤ 50% and *p* > 0.10, a fixed-effects model was used for pooling and analysis; otherwise, a random-effects model was applied. For summary analyses with ≥ 10 studies and substantial heterogeneity (*I*^2^ > 50%), subgroup analyses were conducted to identify sources and provide explanations for the heterogeneity. Sensitivity analyses were performed to evaluate the robustness of the results. Publication bias was assessed using funnel plots. If publication bias was not visually discernible from the funnel plot, Egger's test was used for further assessment. For cases where *p* ≤ 0.05, the trim-and-fill method was employed to evaluate the impact of publication bias on the results. *p* < 0.05 was considered statistically significant for all analyses.

## Results

### Search results

**Fig. 1:**
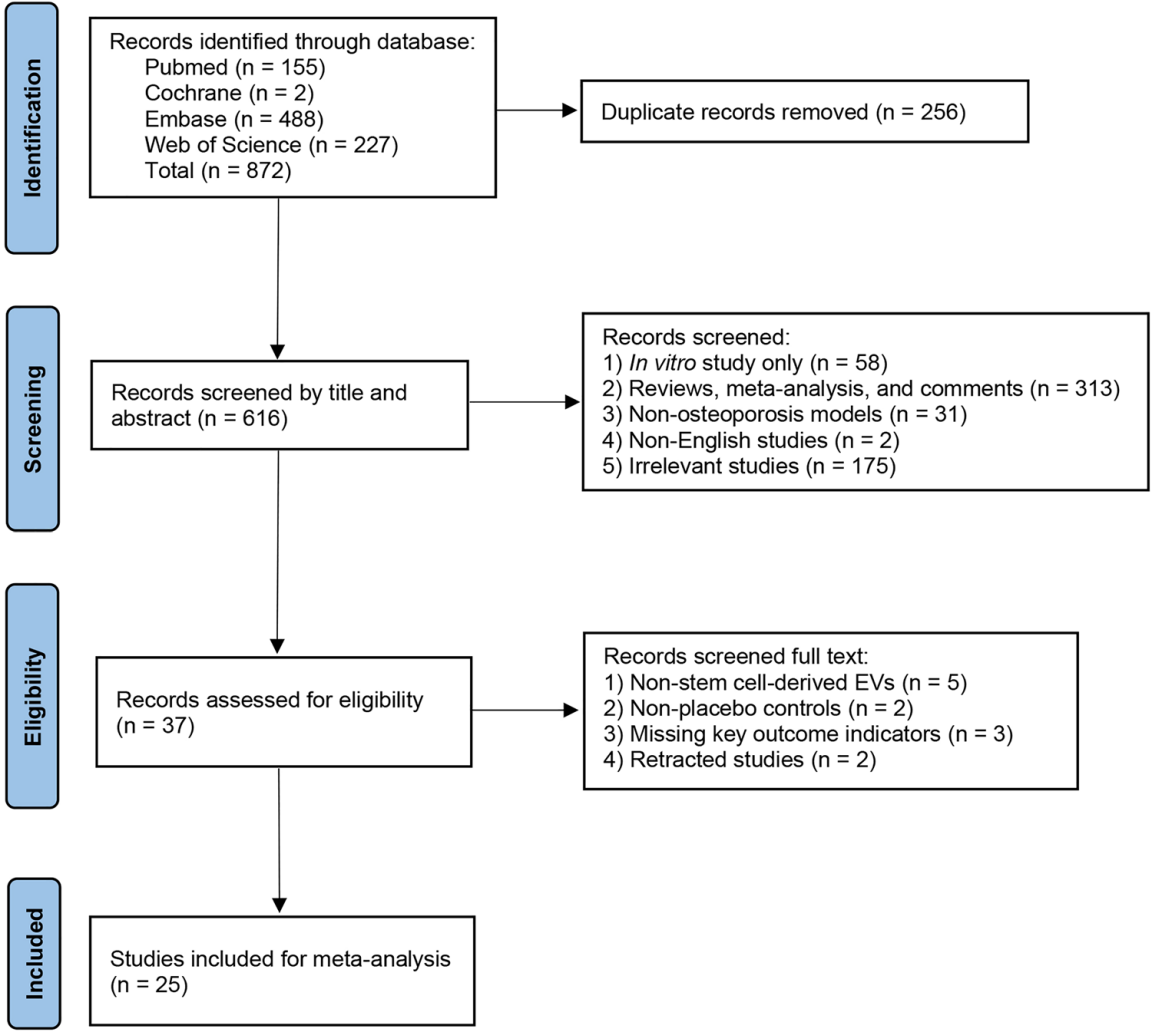
Flowchart of the study based on the PRISMA guidelines

A comprehensive search of the PubMed, Embase, Web of Science, and Cochrane databases, a total of 872 studies were included. After removing duplicates, 256 studies remained. Subsequently, titles and abstracts were screened to identify studies related to SC-EVs for the treatment of osteoporosis. During this process, 579 studies, including in vitro studies, reviews, meta-analyses, letters, commentaries, and non-English articles, were excluded. The remaining 37 studies underwent full-text review to determine their eligibility based on inclusion and exclusion criteria. Sixteen studies were excluded for failing to meet the inclusion criteria. Ultimately, 25 studies [29–53] were included in the meta-analysis. The study selection process is illustrated in Fig. 1.

### Study characteristics

#### General characteristics

The 25 included studies were published between 2016 and 2024. Interestingly, studies published before 2020 accounted for only 8% (2/25), while over 90% were published in 2020 or later, reflecting the growing attention to preclinical research on SC-EVs for osteoporosis treatment in recent years. Of these 25 studies, one was from Korea, and the remaining 24 were from China.

#### Characteristics of animal models

A total of 337 osteoporosis animals were included in the analysis, comprising 169 treated with SC-EVs and 168 receiving only placebo. Table 1 provides detailed characteristics of the animal models in the included studies. Specifically, 14 studies used mice as models, 10 studies employed Sprague Dawley (SD) rats, and 1 study did not specify the species. 18 studies utilized female animals, 5 studies used males, and 2 study did not report the sex of the animals. Regarding animal age, except for 2 studies that did not report it, the age range in the remaining studies was from 7 weeks to 6 months. 9 studies included information on animal body weight. As for osteoporosis modeling methods, the majority of studies (N = 18) used ovariectomy to induce osteoporosis, 3 studies employed intraperitoneal injection of streptozotocin, one study used dexamethasone induction, one used an accelerated aging model, one simulated osteoporosis caused by mechanical unloading, and one study did not provide details on the modeling method.

**Table 1:** Characteristics of animal models included in the 25 studies

Author	Year	Country	Specie	Gender	Age	Weight	Total number	Model of osteoporosis
Chen et al. [29]	2019	China	C57BL/7 mice	Female	8–10 week-old	Not description	30	Ovariectomized
Ge et al. [30]	2021	China	C57BL/6 mice	Female	10-week-old	Not description	40	Ovariectomized
Gong et al. [31]	2020	China	Accelerated senescence mice	Male	6-month-old	Not description	10	Senescence-accelerated mouse prone eight mice
Gui et al. [32]	2024	China	C57BL/6 mice	Female	8-week-old	Not description	50	Ovariectomized
Hu et al. [33]	2020	China	C57BL/6 mice	Female	8-week-old	Not description	30	Ovariectomized
Huang et al. [34]	2021	China	SD rats	Female	10-week-old	230–250 g	40	Ovariectomized
Lee et al. [35]	2021	Korea	ICR (CD‐1) mice	Female	Not description	Not description	24	Ovariectomized
Li et al. [36]	2021	China	SD rats	Female	8-week-old	294 ± 11 g	40	Ovariectomized
Li et al. [37]	2023	China	Not description	Female	6-week-old	Not description	24	Ovariectomized
Li et al. [38]	2024	China	C57BL/6 J mice	Female	2-month-old	Not description	20	Ovariectomized
Lu et al. [39]	2020	China	C57BL/6 J mice	Male	3-month-old	Not description	15	Not description
Lu et al. [40]	2021	China	BALB/c mice	Female	8-week-old	25–30 g	30	Ovariectomized
Qi et al. [41]	2016	China	SD rats	Female	12 weeks old	250–300 g	60	Ovariectomized
Qi et al. [42]	2023	China	SD rats	Female	10 weeks old	230–250 g	18	Ovariectomized
Qiu et al. [43]	2020	China	SD rats	Female	12 weeks old	280–300 g	66	Ovariectomized
Wang et al. [44]	2020	China	SD rats	Female	6-month-old	300–350 g	50	Ovariectomized
Wang et al. [45]	2022	China	C57BL/6 J mice	Female	12 weeks old	28–30 g	40	Ovariectomized
Wang et al. [46]	2023	China	C57BL/6 mice	Female	Not description	Not description	42	Ovariectomized
Xiao et al. [47]	2021	China	C57BL/6 J mice	Male	6-month-old	Not description	20	Osteoporosis caused by mechanical unloading
Xu et al. [48]	2022	China	C57BL/6 J mice	Female	8-week-old	Not description	40	Ovariectomized
Yang et al. [49]	2023	China	SD rats	Not description	8-week-old	200 ± 25 g	50	Intraperitoneally administered with 2.5 mg/kg Dexamethasone once a day
Yao et al. [50]	2024	China	BALB/c mice	Female	7 weeks old	Not description	18	Ovariectomized
Yang et al. [51]	2022	China	SD rats	Male	7–8-week-old	180 ± 20 g	70	Intraperitoneal injection with streptozotocin
Zhang et al. [52]	2021	China	SD rats	Male	8-week-old	Not description	20	Intraperitoneal injection with streptozotocin
Zhang (1) et al. [53]	2021	China	SD rats	Not description	8–10-week-old	Not description	21	Intraperitoneal injection with streptozotocin

*ICR (CD‐1) mice* Improved Castle Road (CD‐1) mice, *SD rats* Sprague Dawley rats

#### SC-EVs characteristics

We included all types of SC-EVs used in the treatment of osteoporosis in animal models (Table 2). Specifically, SC-EVs were derived from bone marrow mesenchymal stem cells (BMSCs) in 12 studies, adipose-derived stem cells (ASC) in 4 studies, human umbilical cord MSCs (hUCMSCs) in 2 studies, urine-derived stem cells (USCs) in 1 study, Wharton’s jelly-derived MSCs (WJ-MSC) in 1 study, Dental pulp stem cells (DPSC) in 1 study and Human embryonic stem cells (hESC) in 1 study. The remaining 3 studies did not specify the type of stem cells. 22 studies reported the diameter of the EV, ranging from approximately 20–5000 nm. Regarding SC-EV isolation methods, the majority of studies (N = 20) employed ultracentrifugation, while 2 studies used polymer precipitation kits, one study used tangential flow filtration (TFF), and one study adopted the ExoEasy Maxi Kit; the remaining study did not specify the method. In terms of purification procedures, 14 studies filtered the EVs through a membrane, 2 studies washed the pellet with PBS, one study purified by resuspending in cold PBS, and the remaining 5 studies did not provide specific details about the purification process. For administration routes, most studies (N = 19) utilized intravenous injection, with others including intraperitoneal injection (N = 2), scaffold loading (N = 1), injection through the periosteum of the femur (N = 1), and gavage treatment (N = 1). One study did not specify the administration route. A total of 25 studies provided details on dosing regimens. 6 administration frequencies were noted: once a week (N = 10), twice a week (N = 5), a single administration (N = 4), thrice a week (N = 3), once a day (N = 2), and every 3 days (N = 1). Additionally, all studies reported treatment durations, which ranged from 1 week to 6 months.

**Table 2:** Characteristics and therapeutic methods of SC-EVs

Author	Year	Characteristics of EV									Therapeutic methods			
		Isolation and purification				EV characterization								
		Source	Cell culture	Isolation	Purification	TEM	Particle concentration	Protein concentration	Marker	Diameter (nm)	Route of administration	Dose of administration	Time of administration	Duration
Chen et al. [29]	2019	hUSC	Cultivate to P2-P6	Polymer precipitation kits	Not description	Cup- or sphere-like morphology	Not description	(4–6) × 10^9^ vesicles	TSG101, CD81, CD63, and CD9	Not description	Intravenously	100 μg	Once a week	2 months
Ge et al. [30]	2021	hUC-MSC	Cultivate to P3	Ultracentrifugation	Filtered through a 0.22 µm sterile filter membrane	Sphere-like morphology	Not description	Not description	CD9, CD63, and TSG101	20–200 μm	Intraperitoneally	0.5 mg/kg	Every 3 days	6 weeks
Gong et al. [31]	2020	hESC	Cultivate hESC line H9	Ultracentrifugation	Filtered through a 0.22 μm pore PES	Cup-shaped morphology	2.1 × 10^11^ ± 1.5 × 10^10^ particles/mL	1114.04 ± 42.66 ng/mL	CD9, CD63, and TSG101	133.2 ± 11.2 nm	Gavage treated	1 × 10^10^ particles/100 μL PBS	Once a day	6 months
Gui et al. [32]	2024	BMSC	BMSCs were treated with staurosporine (0.5 µM) for 6 h	Ultracentrifugation	Not description	Cup-shaped morphology	Not description	Not description	PKH67	220–396 nm	Intravenously	10 mg/kg	Once a week	4 weeks
Hu et al. [33]	2020	hUC-MSC	Cultivate to P2-P6	Ultracentrifugation	Filtered through a 0.22 μm filter	Cup- or sphere-like morphology	Not description	Not description	CD9, CD63, CD81, and TSG101	60–150 nm	Intravenously	100 μg/100 μL PBS	Once a week	3 months
Huang et al. [34]	2021	BMSC	Cultivate to P2-P4	Ultracentrifugation	Filtered through a 0.22 μm filter	Cup- or sphere-like morphology	Not description	100 μg/ml	CD9, CD63, and CD81	40–120 nm	Intravenously	100 μg	Once a week	2 months
Lee et al. [35]	2021	ASC	Cultivate to P5	TFF	Not description	Round shape	Not description	Not description	CD9, CD63, and CD81	88 nm	Intravenously	1 × 10^8^ or 5 × 10^8^ particles/100 μL PBS	Thrice a week	2 weeks
Li et al. [36]	2021	hBMSC	Cultivate	Polymer precipitation kits	Not description	Not description	Not description	Not description	Alixs, CD63, and CD81	100–150 nm	Intravenously	100 μL	Once a week	1 month
Li et al. [37]	2023	BMSC	Cultivate	Not description	Not description	Not description	Not description	Not description	Not description	Not description	Intravenously	Not description	Once a week	4 weeks
Li et al. [38]	2024	MSC	STS inducing apoptosis	Ultracentrifugation	Suspended in ice-cold PBS	Round shape	Not description	4.6 × 10^9^ particles/mL	Annexin V, Histone 3, Cleaved-caspase 3, and CD63	50–5000 nm	Intravenously	100 μg	Once a week	2 months
Lu et al. [39]	2020	BMSC	Cultivate	Ultracentrifugation	Filtered through a 0.22 μm filter	Round shape	1–2 × 10^10^ particles/Ml	Not description	Syntenin 1, and TSG101	30–150 nm	Not description	100 μg	Twice a week	2 months
Lu et al. [40]	2021	WJ-MSC	Cultivate	Ultracentrifugation	Filtered through a 0.22 μm filter	Round shape	Not description	Not description	CD9, CD63, and HSP70	185 nm	Intravenously	200 μg	Once a week	2 months
Qi et al. [41]	2016	hiPSC-MSC	Cultivate to 80–90%	Ultracentrifugation	Filtered through a 0.22 μm filter	Not description	Not description	Not description	CD9, CD63, and CD81	50–150 nm	Scaffold loading	200 µg	Once	2 months
Qi et al. [42]	2023	BMSC	Cultivate to P3	Ultracentrifugation	Filtered through a 0.22 μm filter	Hollow spherical microvesicles	Not description	Not description	CD63, CD81, and TSG101	50–120 nm	Intravenously	100 μg	Once a week	2 months
Qiu et al. [43]	2020	BMSC	Cultivate to P3	ExoEasy Maxi Kit	Filtered through a 0.45 μm filter	Low-density electrons in the vesicles	Not description	Not description	CD63 and CD9	30–100 nm	Intravenously	100 μg	Once a day	2 weeks
Wang et al. [44]	2020	mMSC	Cultivate	Ultracentrifugation	Not description	Round shape	Not description	Not description	Not description	Not description	Intravenously	750 μg	Twice a week	2 months
Wang et al. [45]	2022	MSC	Cultivate	Ultracentrifugation	Filtered through a 0.22 μm filter	Round shape	Not description	Not description	CD63 and CD9	40–100 nm	Injected through periosteum of the femur	20 μL	Twice a week	1 week
Wang et al. [46]	2023	BMSC	Cultivate	Ultracentrifugation	Filtered through a 0.22 μm filter	Cup-shaped morphology	Not description	Not description	CD9, CD63, and CD81	100 nm	Intravenously	100 μg	Twice a week	3 months
Xiao et al. [47]	2021	BMSC	Cultivate to 80–90%	Ultracentrifugation	Filtered through a 0.22 μm filter	Round shape	Not description	Not description	CD63 and TSG101	40–260 nm	Intravenously	100 μL	Twice a week	4 weeks
Xu et al. [48]	2022	BMSC	Cultivate	Ultracentrifugation	Not description	Cup-shaped or flat-shaped	Not description	Not description	CD63, HSP70, TSG101, and GM130	20–200 nm	Intravenously	100 μL	Once	8 weeks
Yang et al. [49]	2022	BMSC	Cultivate to 50–60%	Ultracentrifugation	The pellet was washed with PBS	Cup-shaped morphology	Not description	Not description	Alix, CD63, TSG101, and CD81	500 nm	Intraperitoneally	100 μg	Thrice a week	4 weeks
Yao et al. [50]	2023	ASC	Cultivate to P3	Ultracentrifugation	The pellet was washed with PBS	Round shape	Not description	Not description	HSP70, CD63, and CD81	100 nm	Intravenously	100 μg	Thrice a week	5 weeks
Yang et al. [51]	2024	DPSC	STS inducing apoptosis	Ultracentrifugation	Filtered through a 0.22 μm filter	Cup-shaped morphology	Not description	Not description	CD63, CD81, and TSG101	200 nm	Intravenously	Not description	Once a week	2 months
Zhang et al. [52]	2021	ASC	Cultivate to 50–60%	Ultracentrifugation	Not description	Round shape	4.5 × 10^9^ particles/Ml	Not description	HSP70, TSG101, and CD9	50–100 nm	Intravenously	1.6 mg/kg	Once	12 weeks
Zhang (1) et al. [53]	2021	ASC	Cultivate	Ultracentrifugation	Filtered through a 0.22 μm filter	Round shape	5.5 × 10^9^ particles/mL	Not description	HSP70, TSG101, and CD63	40–100 nm	Intravenously	1.6 mg/kg	Once	6 weeks

*ASC* Adipose-derived stem cell, *BMSC* Bone marrow mesenchymal stem cell, *DPSC* Dental pulp stem cell, *hESC* Human embryonic stem cell, *hUC-MSC* Human umbilical cord mesenchymal stromal cell, *hUSC* Human urine-derived stem cell, *hBMSC* Human bone marrow mesenchymal stem cell, *hiPSC-MSC* mesenchymal stem cells derived from human induced pluripotent stem cell, *MSC* Mesenchymal stem cell, *mMSC* Mouse mesenchymal stem cell, *STS* Staurosporine, *TFF* Tangential flow filtration, *WJ-MSC* Wharton’s jelly-mesenchymal stem cell

### Risk of bias assessment

**Fig. 2:**
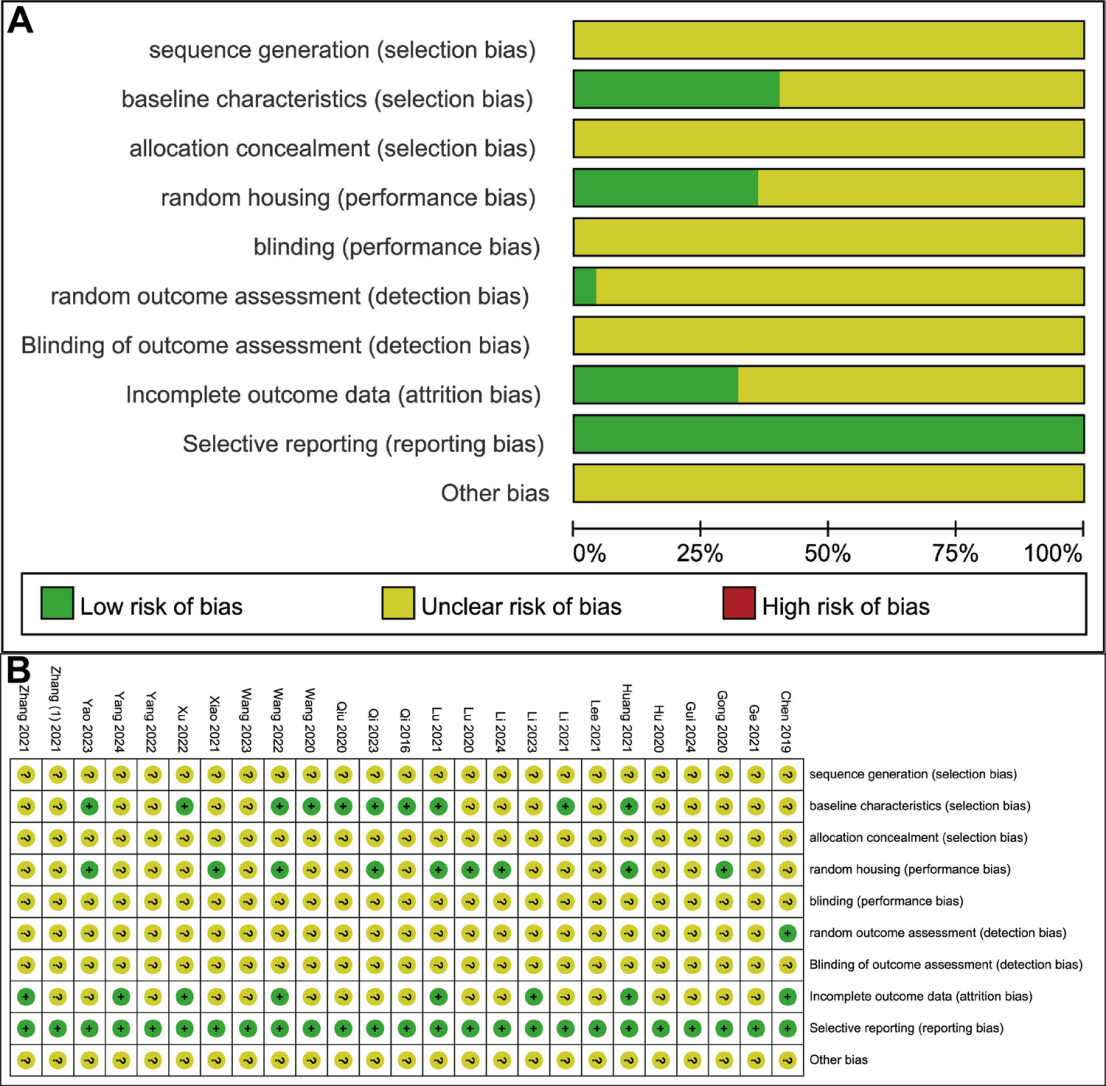
Risk of bias assessment of the 21 included studies based on SYRCLE’s ROB tool. **A** Risk of bias graph; **B** Risk of bias summary

We assessed the quality of the 25 included studies using SYRCLE’s ROB tool. Overall, the majority of studies showed an unclear risk of bias. None of the studies reported or disclosed the methods of animal randomization. 10 studies provided information on key baseline characteristics of the animals, such as age and sex, which are relevant to bone status. None of the studies explicitly detailed whether allocation concealment was implemented. 9 studies described the randomization of animal housing and placement. No studies reported blinding of animal caretakers and researchers. Only one study emphasized the randomization of outcome assessment. None of the studies reported blinding of the outcome evaluators. 8 studies demonstrated a low risk of bias concerning missing data during the experiments. All studies were evaluated as having a low risk of bias for selective outcome reporting. The summary of the risk of bias assessment is shown in Fig. 2A, B.

### Outcomes

#### BMD and BV/TV

**Fig. 3:**
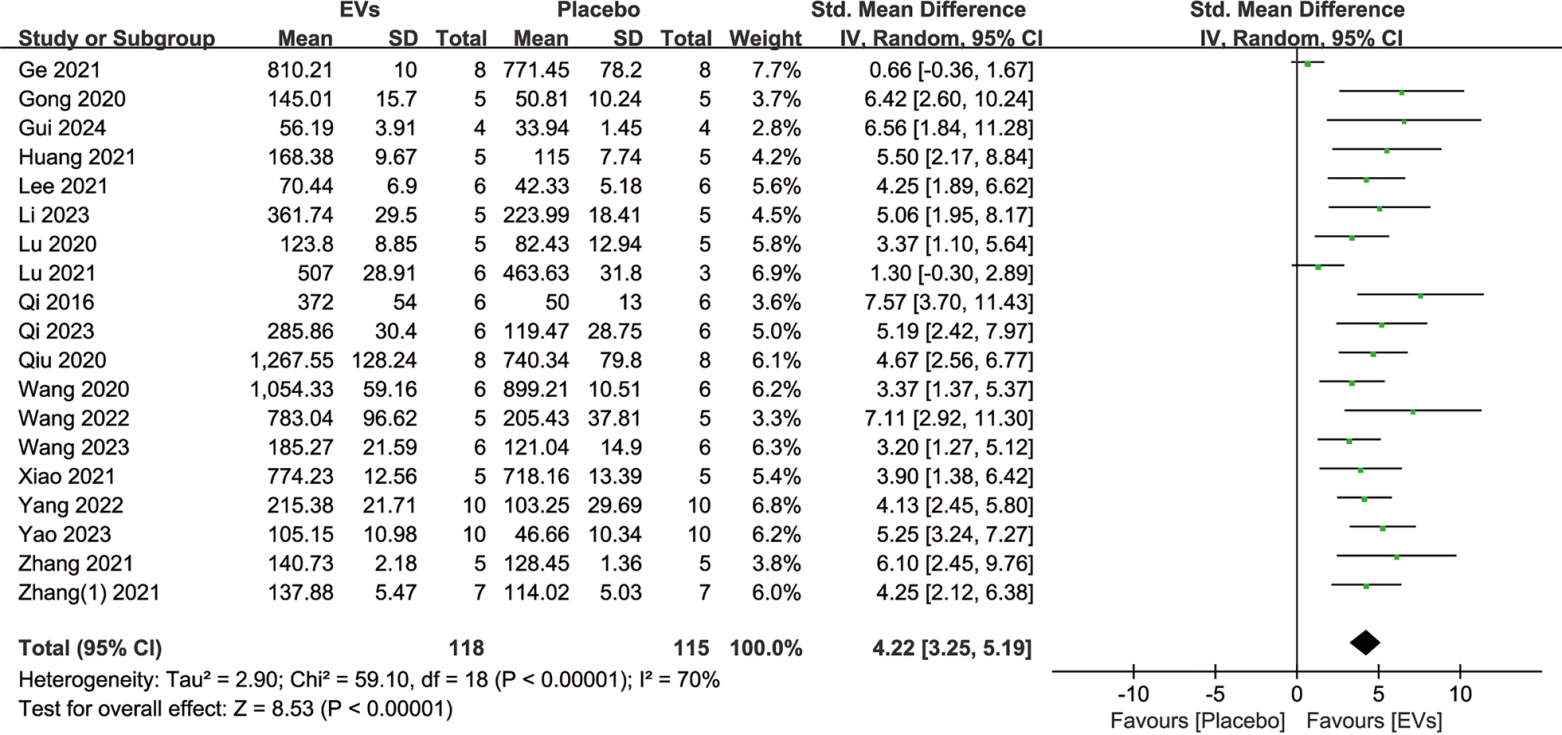
Forest plot showing the difference in bone mineral density (BMD) between SC-EVs treatment and placebo in osteoporosis models. Data are presented as standardized mean difference (SMD) with 95% confidence intervals (CI)

**Fig. 4:**
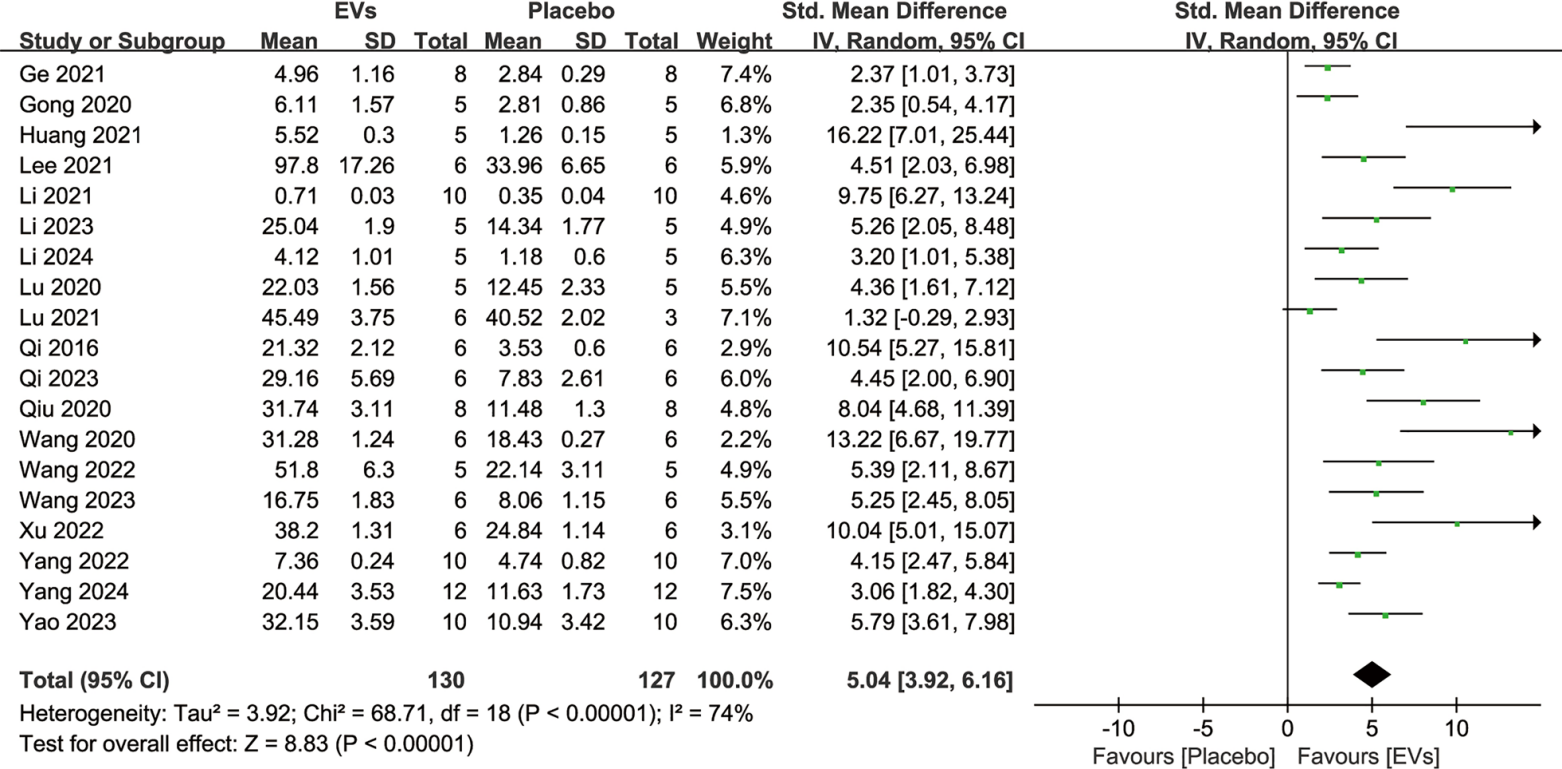
Meta-analysis results of the difference in bone volume fraction (BV/TV) between SC-EVs treatment and placebo in osteoporosis models. Data are presented as standardized mean difference (SMD) with 95% confidence intervals (CI)

19 studies reported quantitative BMD analyses following SC-EVs treatment and placebo, involving a total of 233 animals. The analysis indicated that SC-EVs treatment significantly increased BMD in osteoporosis models compared to placebo (SMD = 4.22, 95% CI 3.25–5.19, *p* < 0.00001) (Fig. 3). However, the heterogeneity test revealed substantial heterogeneity (*I*^2^ = 70%, *p* < 0.00001). Additionally, 19 studies reported BV/TV quantitative data following SC-EVs treatment and placebo. The pooled analysis indicated that SC-EVs treatment significantly increased BV/TV in osteoporosis models (SMD = 3.87, 95% CI 3.35–4.39, *p* < 0.00001) (Fig. 4). Due to significant heterogeneity (*I*^2^ = 74%, *p* < 0.00001), the results should be interpreted with caution.

#### Bone microstructural parameters

**Fig. 5:**
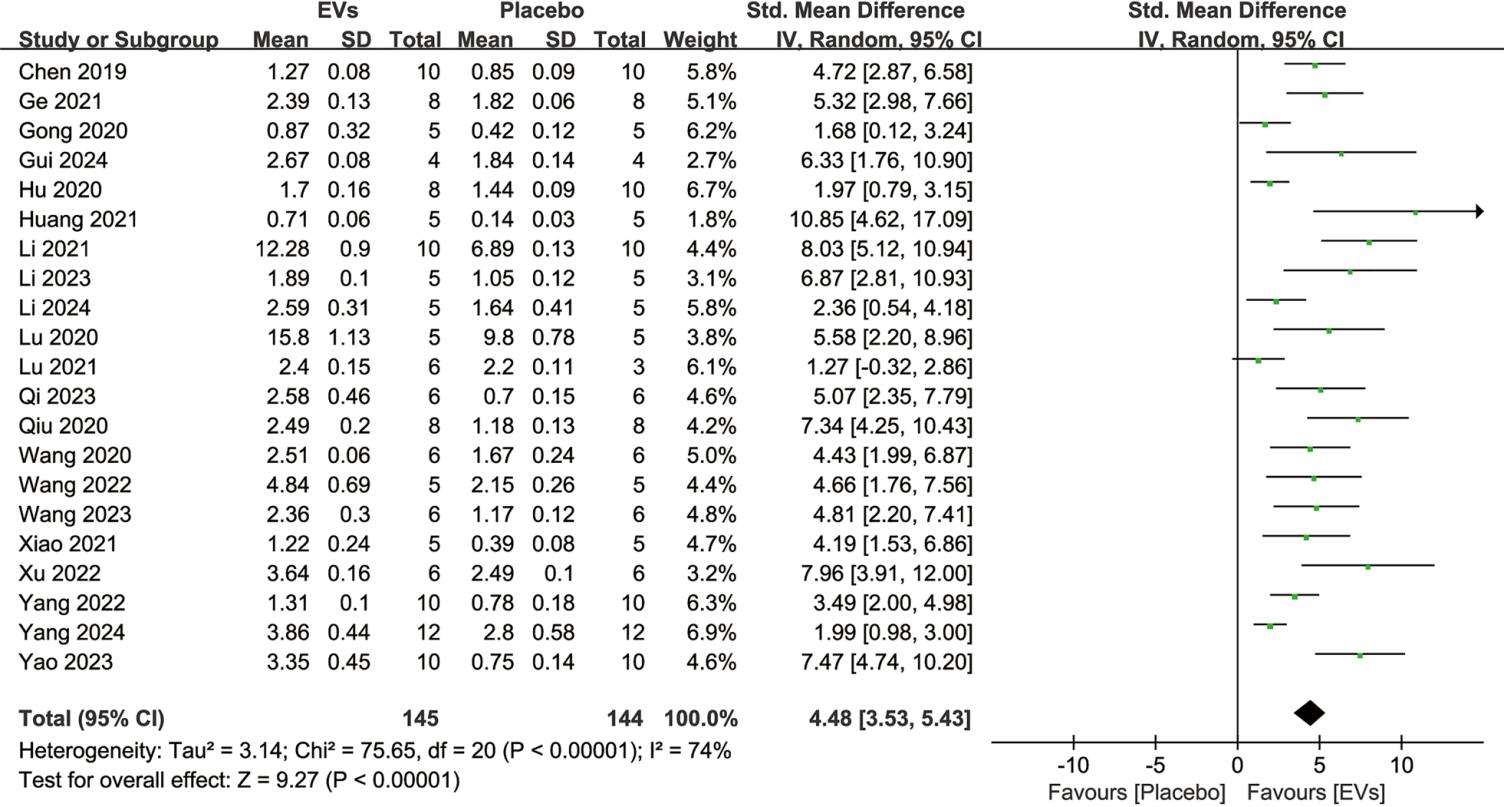
Forest plot showing the difference in trabecular number (Tb. N) between SC-EVs treatment and placebo in osteoporosis models. Data are presented as standardized mean difference (SMD) with 95% confidence intervals (CI)

**Fig. 6:**
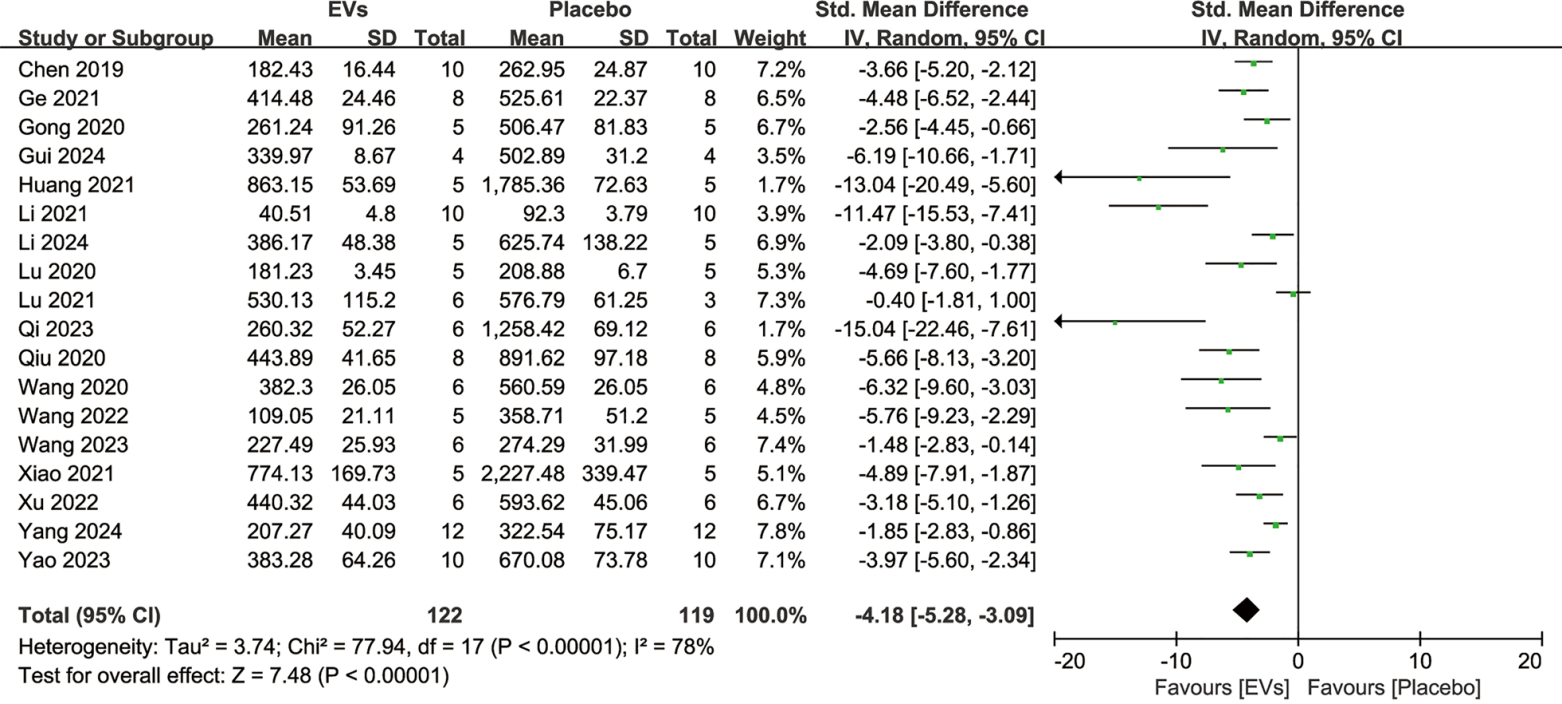
Forest plot describing the difference in trabecular separation/marrow thickness (Tb. Sp) between SC-EVs treatment and placebo in osteoporosis models. Data are presented as standardized mean difference (SMD) with 95% confidence intervals (CI)

**Fig. 7:**
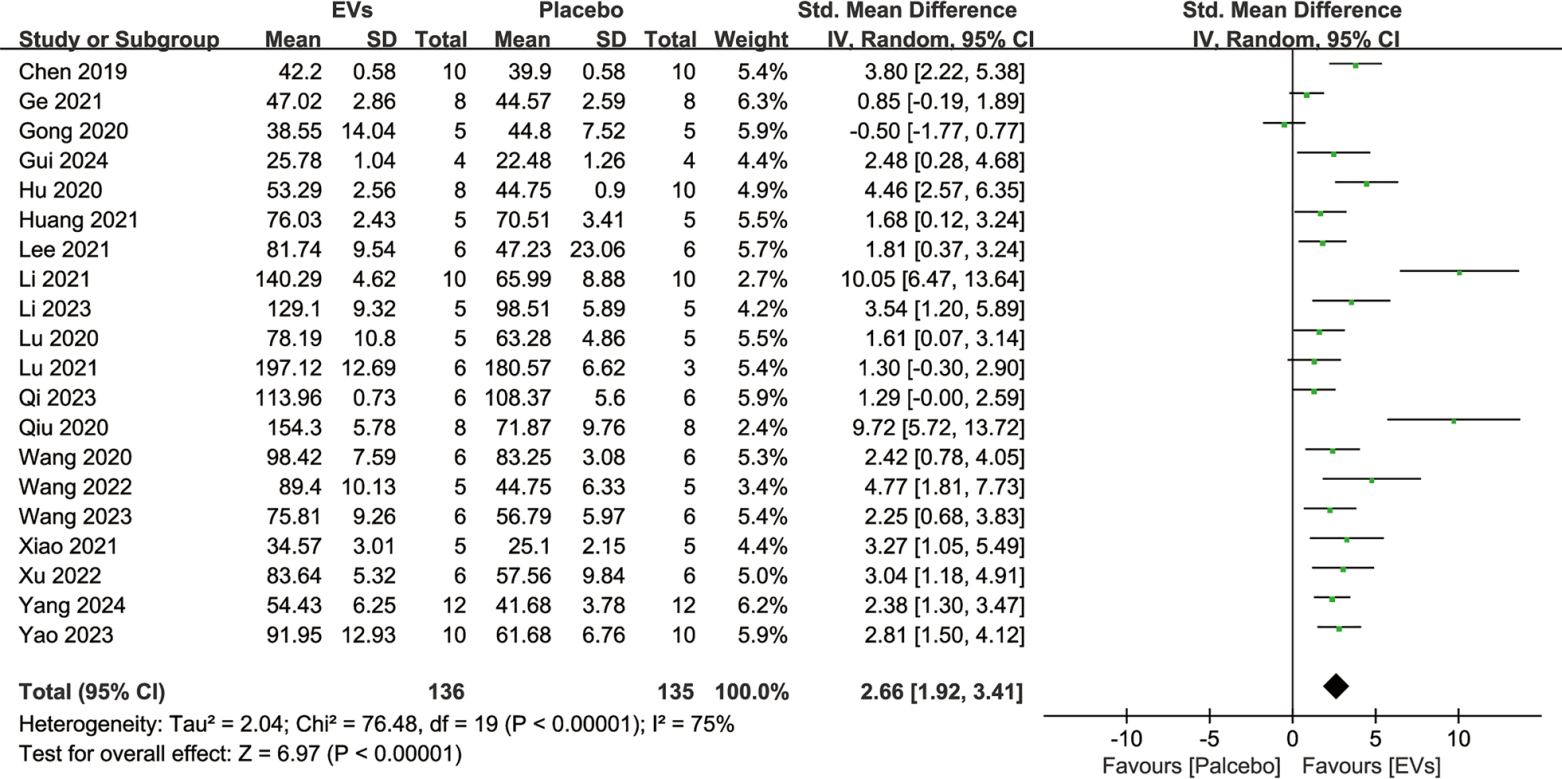
Forest plot depicting the difference in trabecular thickness (Tb. Th) between SC-EVs treatment and placebo in osteoporosis models. Data are presented as standardized mean difference (SMD) with 95% confidence intervals (CI)

Bone microstructural parameters reflect the microarchitectural characteristics of bone and can be used to evaluate the extent of repair in osteoporosis models. 21 studies reported quantitative analysis results of Tb.N involving 289 animals. The results showed that SC-EVs treatment increased the trabecular number in osteoporosis models (SMD = 3.38, 95% CI 2.94–3.82, *p* < 0.00001) (Fig. 5). 18 studies reported quantitative analysis results of Tb.Sp involving 241 animals. The results indicated that SC-EVs treatment reduced Tb.Sp in osteoporosis models (SMD = −2.94, 95% CI −3.40 to −2.48, *p* < 0.00001) (Fig. 6). 20 studies reported quantitative analysis results of Tb.Th involving 271 animals. The results showed that SC-EVs treatment increased Tb.Th in osteoporosis models (SMD = 2.14, 95% CI 1.79–2.50, *p* < 0.00001) (Fig. 7). Due to the presence of significant heterogeneity (*I*^2^ > 50%, *p* < 0.00001), the beneficial effects of SC-EVs on bone microstructural parameters should be interpreted with caution.

#### Other quantitative bone parameters

**Fig. 8:**
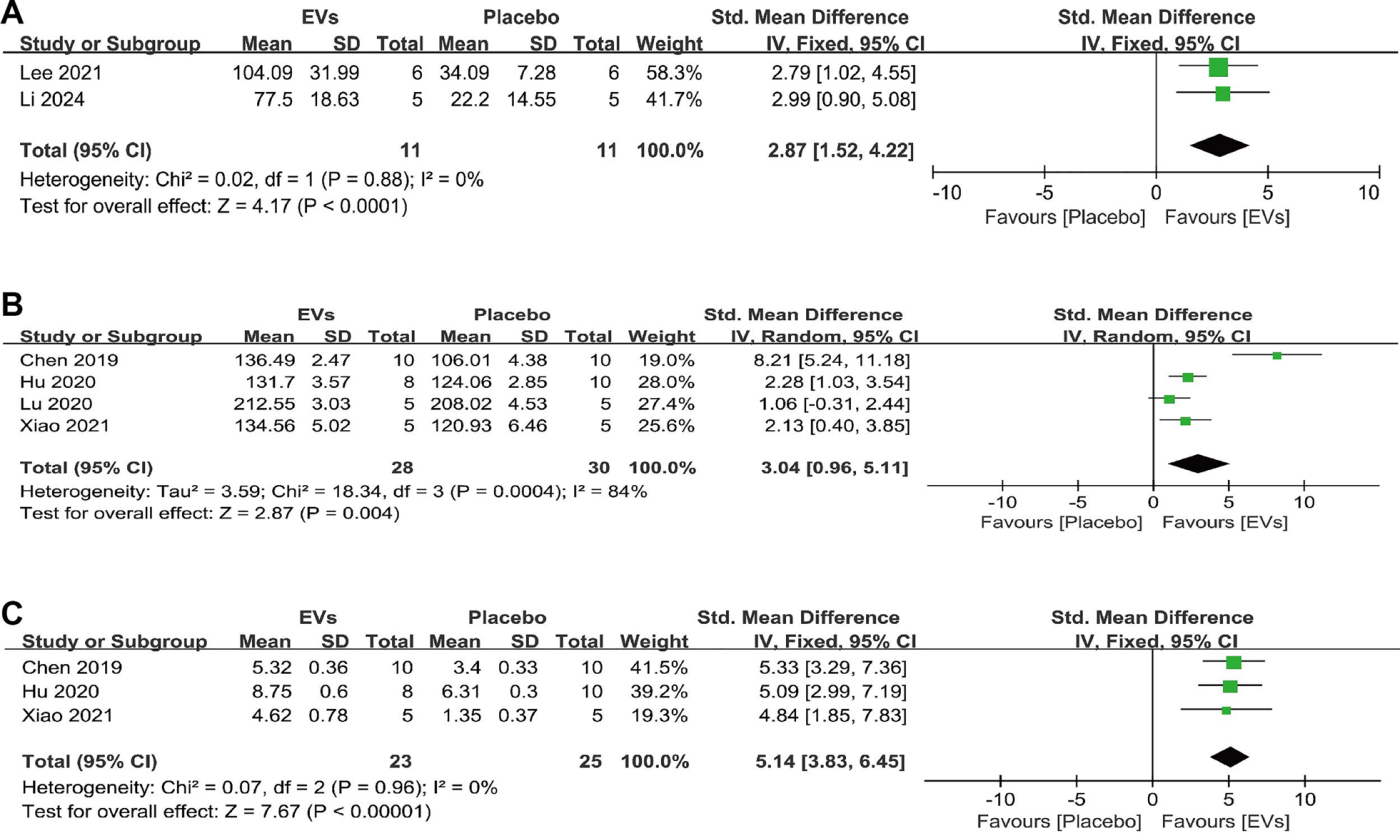
Forest plots describing the effects of SC-EVs treatment on other skeletal parameters in osteoporosis models: **A** Bone volume (BV); **B** Cortical thickness (Ct. Th); **C** Bone volume fraction (trabecular bone volume/total volume, Tb. BV/TV). Data are presented as standardized mean difference (SMD) with 95% confidence intervals (CI)

**Fig. 9:**
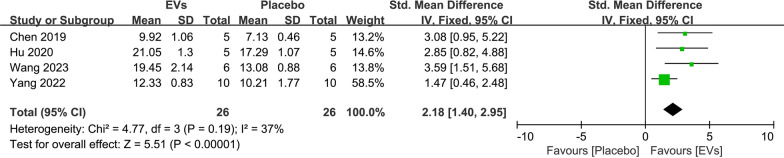
Forest plot showing the difference in femur ultimate load between SC-EVs treatment and placebo in osteoporosis models. Data are presented as standardized mean difference (SMD) with 95% confidence intervals (CI)

We also conducted a meta-analysis of other bone quantitative data. For BV, the combined analysis of two studies showed that SC-EVs treatment can increase BV in osteoporosis models (SMD = 2.87, 95% CI 1.52–4.22, *p* < 0.0001) (Fig. 8A). Four studies reported Ct. Th data, and the analysis showed that SC-EVs treatment can increase cortical bone thickness (SMD = 3.04, 95% CI 0.96–5.11, *p* = 0.004) (Fig. 8B). However, the combined heterogeneity was high (*I*^2^ = 84%, *p* = 0.0004), so caution should be exercised when interpreting this result. For Tb. BV/TV, the combined analysis indicated that SC-EVs can improve the trabecular bone volume fraction in osteoporosis models (SMD = 5.14, 95% CI 3.83–6.45, *p* < 0.00001) (Fig. 8C). These results suggest that, compared to placebo, SC-EVs have a positive effect on increasing bone mass (both cortical and trabecular bone) in osteoporosis models. Additionally, 4 studies reported the femur ultimate load after treatment. The combined analysis showed that, compared to placebo, the femur ultimate load was greater after SC-EVs treatment (SMD = 2.18, 95% CI 1.40–2.95, *p* < 0.00001) (Fig. 9).

#### Histological quantitative analyses

**Fig. 10:**
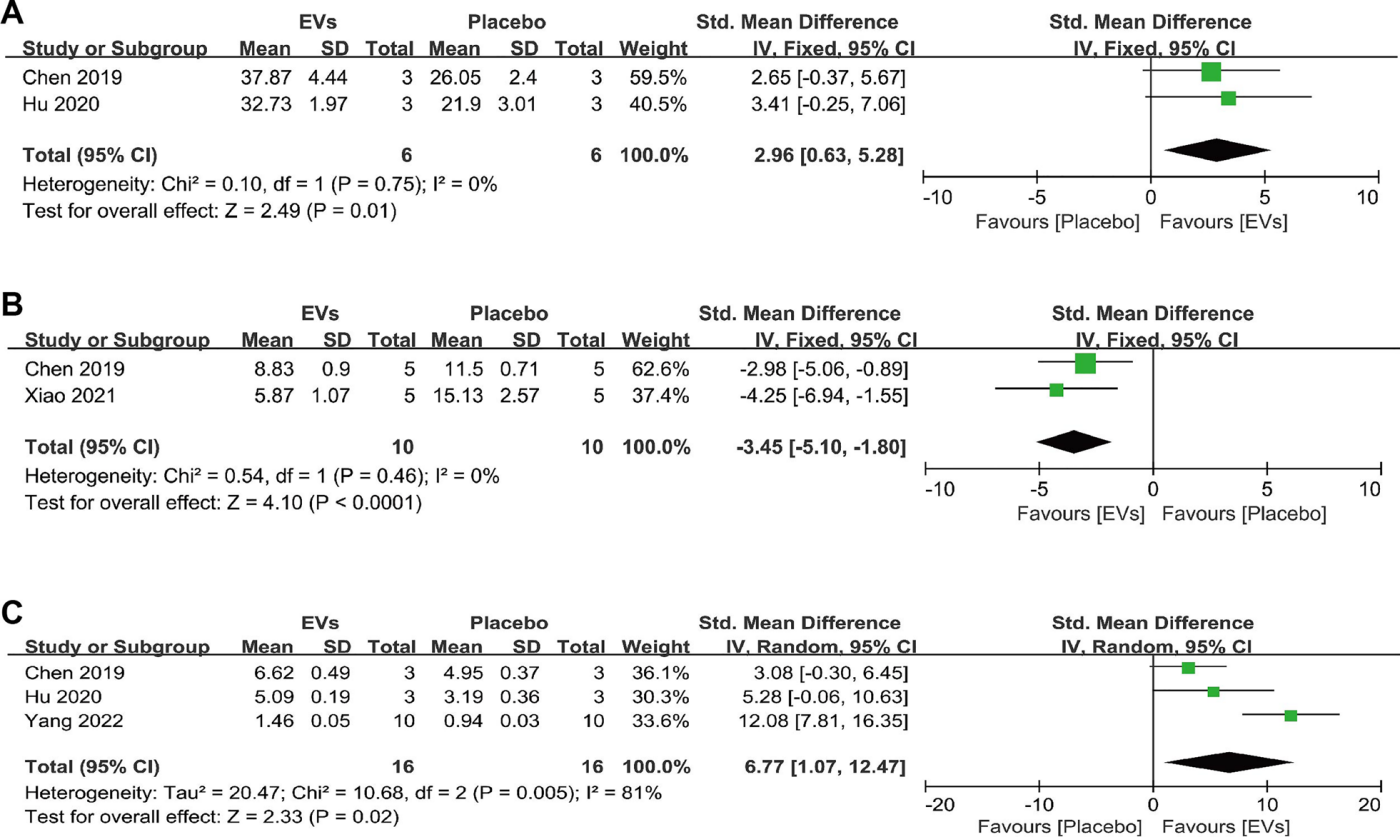
Forest plots depicting the differences in histological quantitative results between SC-EVs treatment and placebo in osteoporosis models: **A** Number of osteoblasts per trabecular bone surface (N. OBs/BS); **B** Number of osteoclasts per trabecular bone surface (N. OCs/BC); **C** Mineral apposition rate (MAR). Data are presented as standardized mean difference (SMD) with 95% confidence intervals (CI)

The occurrence and progression of osteoporosis are often accompanied by an imbalance between osteoblasts and osteoclasts. To explore this, we collected quantitative evidence from in vivo histological staining to investigate the effects of SC-EVs treatment on osteoclasts and osteoblasts in osteoporosis models. For the number of osteoblasts in trabecular bone (N. OBs/BS), the combined analysis showed that SC-EVs treatment can increase osteoblast number (SMD = 2.96, 95% CI 0.63–5.28, *p* = 0.01) (Fig. 10A), while also decreasing osteoclast number (N. OCs/BS) (SMD = −3.45, 95% CI −5.10 to −1.80, *p* < 0.0001) (Fig. 10B). Interestingly, we also found that SC-EVs treatment can increase the mineral apposition rate (MAR) (SMD = 6.77, 95% CI 1.07–12.47, *p* = 0.02) (Fig. 10C). However, the combined result showed high heterogeneity (*I*^2^ = 81%, *p* = 0.005), so caution should be exercised when interpreting this result. The combined histological staining results indicate that, compared to placebo, SC-EVs treatment helps to increase osteoblast number and bone mineralization in osteoporosis models, while reducing osteoclast number. This may explain, at the cellular level, the potential mechanisms by which SC-EVs improve bone mass.

#### Bone turnover markers quantified by ELISA

**Fig. 11:**
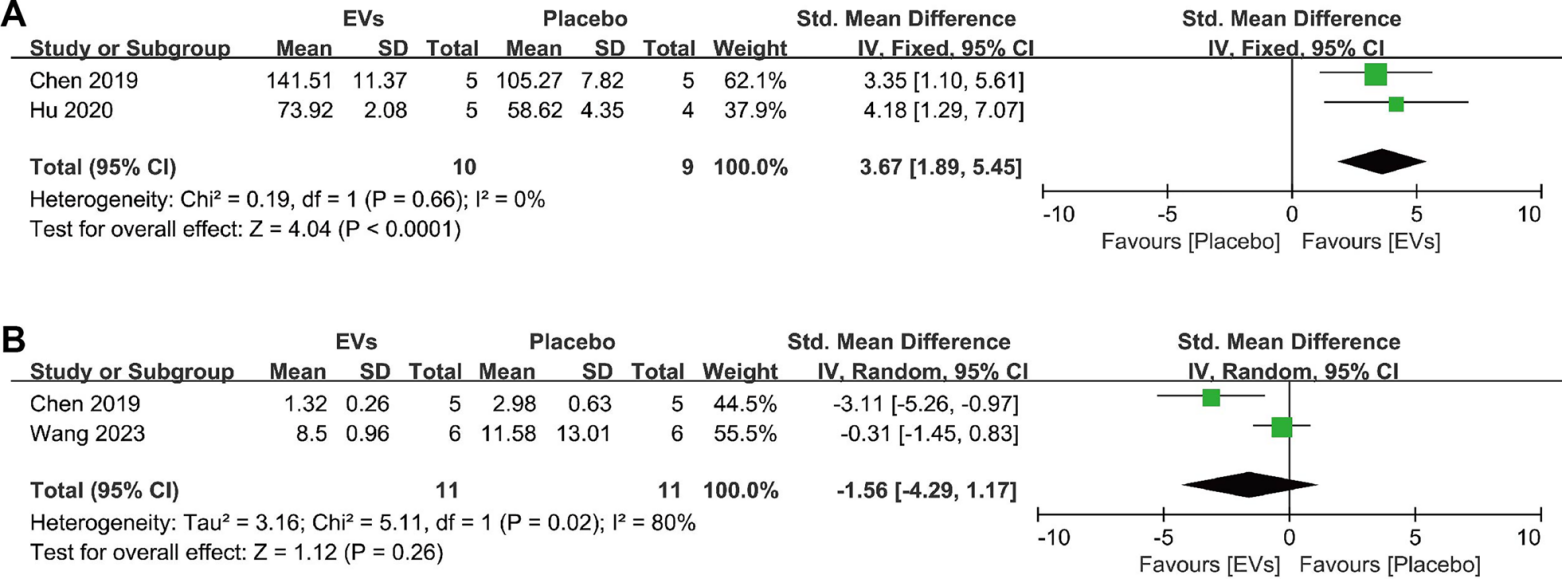
Forest plots showing the differences in bone turnover marker quantification results between EVs treatment and placebo in osteoporosis models: **A** Osteocalcin (OCN); **B** C-terminal telopeptides of type I collagen (CTX-I). Data are presented as standardized mean difference (SMD) with 95% confidence intervals (CI)

Additionally, we examined data on bone metabolism markers detected by ELISA following SC-EVs treatment. The results showed that SC-EVs promoted the expression of serum OCN, suggesting an accelerated rate of bone formation (SMD = 3.67, 95% CI 1.89–5.54, *p* < 0.0001) (Fig. 11A). However, SC-EVs had no significant effect on the expression of serum CTX1 (*p* = 0.26) (Fig. 11B). Given the significant heterogeneity (*I*^2^ = 80%, *p* = 0.02) and the limitation of sample size, these results should be interpreted with caution.

#### Subgroup analysis

Considering that different characteristics of EVs, including their origin, preparation, characterization, and intervention methods, as well as variations in animal models (such as sex, age, and modeling approaches) may influence the therapeutic efficacy of SC-EVs in osteoporosis models, we further conducted subgroup analyses to investigate the potential sources of significant heterogeneity.

Based on the distinct characteristics of EVs described in the recent MISEV2023 guidelines [54], the included studies were categorized according to EV source, isolation method, purification method, EV size, route of administration, frequency of intervention, dosage, and treatment duration. Subgroup analysis revealed that EVs derived from bone marrow mesenchymal stem cells (BMSCs), isolated via ultracentrifugation, purified through filtration, classified as small EVs (< 200 nm), administered intravenously, and applied at intervention frequencies of once or twice weekly, at doses ≤ 100 μl/μg, and for treatment durations of < 2 months or ≥ 2 months, all significantly increased BMD, BV/TV, Tb.N, and Tb.Th, while reducing Tb.Sp (Supplementary Figures S1–S40). Furthermore, subgroup analyses based on animal model characteristics were conducted, including animal age, sex, and modeling method. The results indicated that SC-EVs consistently enhanced BMD, BV/TV, Tb.N, and Tb.Th, and decreased Tb.Sp across subgroups of different sexes (male and female), ages (immature and adult), and modeling approaches (ovariectomized models) (Supplementary Figures S41–S55). However, due to the persistence of significant heterogeneity, none of these subgroup variables were identified as definitive sources of heterogeneity.

Due to limitations such as small sample sizes and significant heterogeneity in the subgroup analyses, further evidence is required to substantiate the findings; therefore, the results should be interpreted with caution. Moreover, although we conducted a comprehensive investigation of the therapeutic effects of each subgroup on osteoporosis models, no definitive sources of heterogeneity were identified. This highlights the need for future studies to focus on the standardization of SC-EV preparation, intervention protocols, and animal modeling approaches, in order to reduce heterogeneity and enhance the overall quality of evidence.

#### Publication bias

For BMD, the evaluation of publication bias revealed asymmetry in the included studies, confirmed by Egger’s test results (t = 8.78, *p* = 0.000 < 0.05) (Supplementary Figure S56, Table S1). To address this, the trim-and-fill method was employed to comprehensively assess the stability of the results. By adding seven virtual studies and recalculating the pooled effect size, the results showed Q = 113.015, *p* = 0.000 < 0.05, pooled Est = 28.029 (95% CI 10.222–76.859) (Supplementary Table S2), indicating that the direction of pooled effect size remained unchanged, demonstrating the robustness of the pooled effect size. Additionaly, we assessed publication bias for key outcome indicators, including BV/TV, Tb.N, Tb.Sp, and Tb.Th, using funnel plots and Egger’s test. For outcomes showing evidence of publication bias, the trim-and-fill method was applied. The results indicated significant publication bias for BV/TV, Tb.N, and Tb.Th (*p* = 0.000 < 0.05) (Supplementary Figures S57–S59, Tables S3–S5). However, after incorporating hypothetical studies using the trim-and-fill method, the direction of the pooled effect sizes remained unchanged, supporting the robustness and reliability of the meta-analysis findings. Moreover, for Tb.Sp, the trim-and-fill method showed “no trimming performed, no new studies added, and data unchanged” (Supplementary Figure S60). Therefore, the stability of the Tb.Sp result will be further evaluated through sensitivity analysis.

#### Sensitivity analysis

**Fig. 12:**
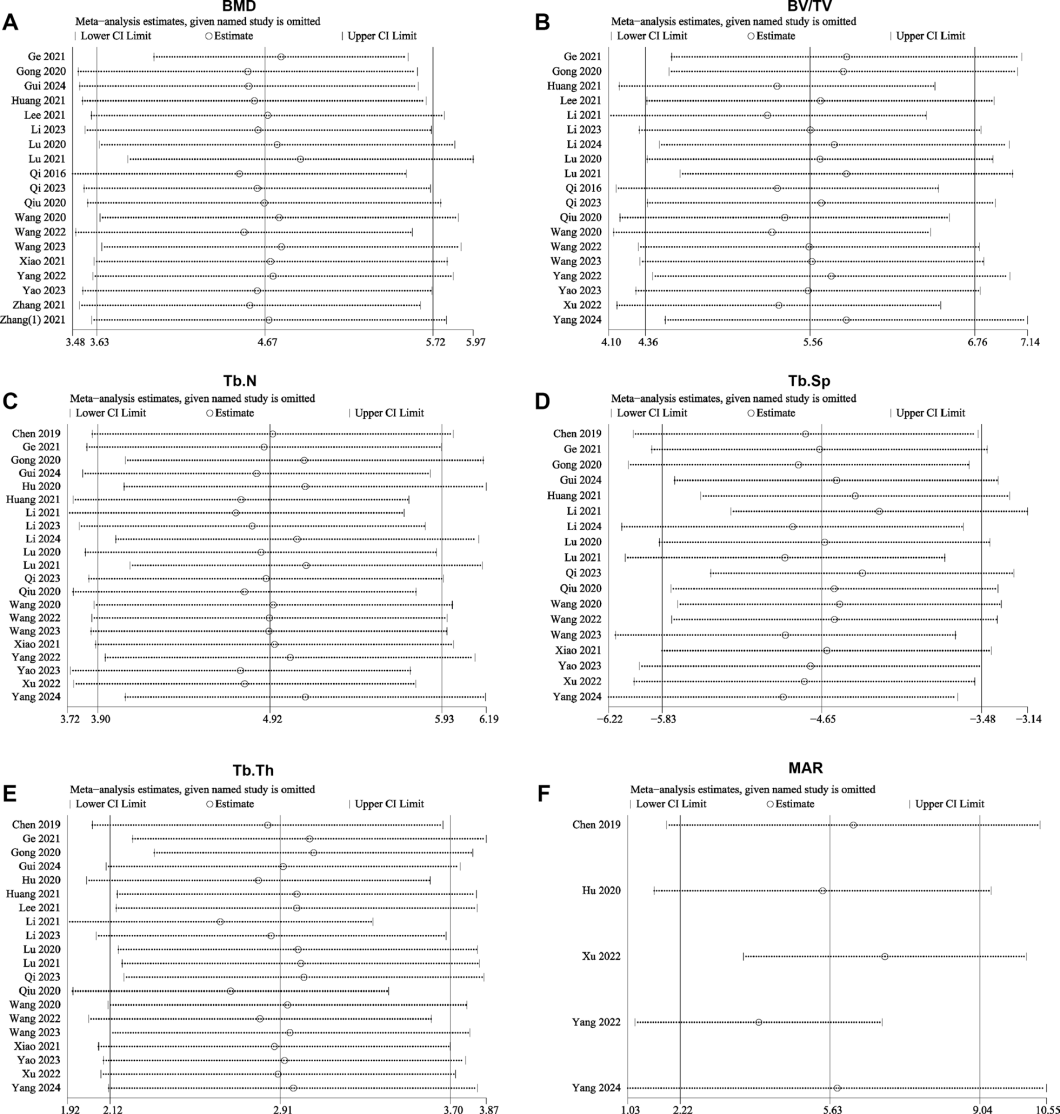
Sensitivity analysis results for various outcome measures. **A** Bone mineral density (BMD); **B** Bone volume fraction (BV/TV); **C** Trabecular number (Tb. N); **D** Trabecular separation/marrow thickness (Tb. Sp); **E** Trabecular thickness (Tb. Th); **F** Mineral apposition rate (MAR)

Sensitivity analysis of the BMD showed that excluding individual studies did not significantly alter the pooled effect size, further supporting the stability of the SMD results (Fig. 12A). Furthermore, the “leave-one-out” sensitivity analyses for BV/TV, Tb.N, Tb.Sp, and Tb.Th indicated that sequentially excluding each individual study did not alter the direction of the pooled effect sizes. This consistency confirms the stability and robustness of the results for these outcome measures.

## Discussion

This systematic review and meta-analysis collected the latest preclinical studies from the past 5 years on the treatment of osteoporosis using SC-EVs. It preliminarily explored the efficacy of this emerging technology in treating animal models of osteoporosis, focusing on three main aspects: general bone analysis parameters, histological quantification, and serum bone turnover markers. The pooled analysis results demonstrated that, compared to placebo, SC-EVs treatment significantly promoted bone repair and regeneration in osteoporosis models. At the microscopic level, analysis based on a limited number of studies and sample sizes suggested that SC-EVs may exert positive effects by increasing the number of osteoblasts and bone mineralization rate within trabecular bone, reducing the number of osteoclasts, and upregulating serum OCN expression. Additionally, we included osteoporosis animal models induced by various methods, such as the diabetic osteoporosis model, accelerated aging model, and mechanical unloading model, with the aim of obtaining more comprehensive and generalized results. This meta-analysis is the first to conduct further subgroup analysis based on different EV characteristics (including EV source, isolation method, purification method, EV size, dosage, route of administration, frequency, and duration of intervention) as well as animal model characteristics (such as sex, age, and modeling method), in order to study the effect differences under various factors.

### Risk of bias and heterogeneity

Since 2020, SC-EVs have garnered significant attention in the field of osteoporosis treatment, with a rapid increase in the number of studies. In addition to summarizing the bone repair potential of SC-EVs in osteoporosis animal models, we also focused on the reporting quality, risk of bias, and heterogeneity of merged effect sizes in the included studies. First, 21 studies exhibited numerous unclear risk-of-bias characteristics, primarily in four areas: methods of sequence generation, allocation concealment, implementation of blinding, and blinding during outcome assessment. While most studies reported random group allocation, they lacked detailed descriptions of the methods used for randomization and allocation concealment. Furthermore, we did not observe detailed descriptions of blinding in experiments and outcome evaluations. 9 studies provided detailed data on animal sex, age, and weight, which were considered to have balanced baseline characteristics and thus posed a low risk of bias. All studies demonstrated low risk of bias in selective reporting. Overall, while most studies showed unclear risks of bias, the overall risk level was acceptable. Future preclinical trials of SC-EVs for osteoporosis should emphasize the importance of providing detailed descriptions of selection bias and blinding methods.

For the heterogeneity observed in the five analyzed outcomes (BMD, BV/TV, Tb.N, Tb.Sp, and Tb.Th), we conducted subgroup analyses to explore potential sources of heterogeneity. The grouping was based on different EV characteristics (including EV source, isolation method, purification method, EV size, dosage, route of administration, frequency, and duration of intervention) as well as animal model characteristics (such as sex, age, and modeling method). However, the subgroup analysis revealed that the within-group heterogeneity remained significant (*I*^2^ > 50%), indicating that these grouping characteristics were not the sources of heterogeneity. Therefore, the interpretation of these results should be approached with caution.

### The positive effects of SC-EVs on bone repair

Osteoporosis is a metabolic bone disease primarily characterized by increased bone fragility, reduced trabecular bone, and microstructural damage [55, 56]. Improving or reversing bone microstructural damage is a key strategy for treating osteoporosis patients. While anti-resorptive and bone anabolic drugs are clinically used, their long-term side effects and limited efficacy have driven the development of novel therapeutic approaches [57]. SC-EVs have been shown to induce osteoblast differentiation and repair damaged MSCs [33]. Their low immunogenicity (lacking MHC I and MHC II proteins) and stable biocompatibility have attracted significant attention in the field of osteoporosis treatment [14, 58]. For example, a previous study combined SC-EVs with alendronate sodium, which maintained bone affinity while significantly improving bone loss in hormone-induced osteoporotic rats [44]. Lu et al. [40] demonstrated the beneficial effects of WJ MSC-EVs on osteoporotic mice by observing osteoblast activity and bone microstructure. In our meta-analysis, SC-EVs also significantly improved various bone microstructural parameters in osteoporosis models, including BMD, BV/TV, Tb.N, Tb.Sp, Tb.Th, BV, Ct.Th, and Tb.BV/TV, highlighting the positive role of SC-EVs in promoting bone repair.

Additionally, we conducted subgroup analyses based on different characteristics of SC-EVs to explore potential sources of heterogeneity and preliminarily investigate the efficacy of various subgroups on osteoporosis models. For SC-EVs derived from different sources, the analysis indicated that, compared to placebo, the BMSC-EVs subgroup significantly increased BMD, BV/TV, and trabecular number and thickness while notably reducing trabecular separation. Wang et al. [46] also demonstrated that BMSC-EVs improved bone mass and skeletal strength in ovariectomy-induced osteoporotic mice while mitigating trabecular bone loss. Moreover, the adipose tissue-derived stem cells-EVs (ASC-EVs) subgroup showed significant increases in BMD, BV/TV, and Tb.Th, reflecting the potential bone repair effects of ASC-EVs. A previous study reported that intravenous injection of ASC-EVs extended their retention time in bone tissue and effectively alleviated bone loss in osteoporotic mice [35].

Additionally, Subgroup analyses based on different isolation methods, purification techniques, and EV sizes revealed that the ultracentrifugation subgroup, the filtered-through-a-filter subgroup, and the small EVs subgroup significantly improved bone mass and strength in osteoporosis models. It is worth noting that different separation and purification methods may induce varying degrees of EV damage, which in turn affects immunogenicity [59]. For instance, ultracentrifugation and dead-end filtration may cause membrane damage, EV aggregation, and biomolecular damage [60]. Among the 25 studies included, 20 used ultracentrifugation, 2 employed precipitation kits, and 1 used Tangential Flow Filtration (TFF). This may be a significant reason for the substantial heterogeneity (*I*^2^ > 50%) observed in the BMD, BV/TV, Tb.Th, Tb.N, and Tb.Sp results from the meta-analysis based on the ultracentrifugation method. In contrast, TFF, due to its controlled shear rate, can serve as a gentle EV separation method to minimize EV damage [61]. However, only one of the 25 studies utilized this technique. Additionally, ultracentrifugation and precipitation kits can lead to the co-sedimentation of large amounts of protein contaminants, which may also affect dosing accuracy [61]. Regarding the size of EVs, a systematic review based on the MISEV2018 guidelines, which classified EVs into large EVs and small EVs, indicated that these two sizes of EVs exhibit different biodistribution and accumulation patterns [62]. For instance, small EVs appear first in the liver and kidneys following intravenous injection, whereas large EVs are initially observed in the lungs. This differential distribution pattern may result in variations in the specific effects on the osteoporosis model, potentially influencing the heterogeneity of the results.

In terms of administration methods, intravenous injection of EVs is the most common approach. We observed that intravenous injection improved BMD, BV/TV, Tb.N, and Tb.Th while reducing Tb.Sp. Intraperitoneal injection of SC-EVs significantly increased BV/TV and Tb.N, but due to the limited number of studies, the effect on Tb.Sp could not be evaluated. Given the insufficient concentration of SC-EVs at local lesions, intravenous and intraperitoneal administration may require engineering strategies to enhance bone targeting and biodistribution. For instance, biomaterial-based delivery systems, such as hydrogels [63, 64], bio-scaffolds [65, 66], and membranes [67], have been shown to improve the release characteristics of SC-EVs. A previous study demonstrated that loading EVs onto GelMA and HAMA composite hydrogels allowed for a sustained release for up to 17 days in vitro and promoted the repair of rat calvarial defects [68]. Notably, the intervention dosage of SC-EVs may also influence their therapeutic efficacy in osteoporosis models. However, the existing literature on EV dosing is mostly focused on small animal models, which may not be consistent with human metabolic conditions. In a study on non-human primates, Driedonks et al. [69] found that repeated intravenous injections significantly reduced the circulation time of EVs, potentially involving innate immune memory in resident macrophages and accelerated blood clearance, which could be a key limitation of repeated or high-dose EV interventions. As observed in the subgroup analysis, we found that two intervention frequencies (once a week and twice a week), as well as different treatment durations (< 2 months and ≥ 2 months subgroups), contributed to increased bone mass and strength. The accelerated blood clearance after repeated intravenous injection may affect the efficacy of different EV intervention doses and frequencies in treating the osteoporosis model. Therefore, given the high heterogeneity, these findings should be interpreted with caution. However, existing studies have primarily emphasized the critical role of administration routes and targeted delivery [35, 40], while lacking sufficient exploration into standardized dosing regimens or treatment durations. Future studies should focus on standardized dosage regimens and optimized administration methods to better evaluate the efficacy of SC-EVs in treating osteoporosis.

In animal models, among the 25 studies included, the majority used ovariectomy-induced osteoporosis, with other modeling methods including intraperitoneal injection of streptozotocin, dexamethasone induction, accelerated aging models, and mechanical unloading. In the ovariectomy-induced animal model, bone loss is related to estrogen deficiency [70]. However, there are differences in the timing of bone loss between rat and mouse models. For instance, in rats, significant bone loss occurs in the proximal tibial metaphysis 14 days after ovariectomy, gradually extending to the lumbar vertebrae [71]. In mice, trabecular bone loss occurs 10 days after ovariectomy [72]. In the subgroup analysis of this study, the summary results of BMD, BV/TV, Tb.Th, Tb.N, and Tb.Sp in the ovariectomy group still showed significant heterogeneity, which may be due to the presence of both rat and mouse models within this subgroup. Although the streptozotocin-induced diabetic osteoporosis model subgroup used SD rats, differences in age and body weight may contribute to the heterogeneity of the results. As for the accelerated aging model and mechanical unloading model, due to limitations in the number of studies and sample sizes, no clear conclusions can be drawn for these subgroups. Future research based on SC-EV interventions in these two models is still needed for further analysis.

CTX-1 is a widely recognized bone resorption-specific biomarker used for monitoring osteoporosis treatment and predicting fracture risk. Specifically, CTX-1 is a degradation product of type I collagen, released during osteoclastic bone resorption, and thus serves as a surrogate marker of OC activity [73, 74]. Interestingly, we observed that SC-EV intervention reduced the number of OCs, yet did not lead to a decrease in serum CTX-1 levels in animals. A possible explanation is that, despite the reduction in OC number, the activity of the remaining cells may be sufficient to maintain CTX-1 levels, potentially resulting in a temporal dissociation between the decrease in OC number and serum CTX-1 concentration. On the other hand, changes in bone tissue morphology are not entirely consistent with the levels of serum markers such as CTX-1. Makras et al. [75] found in a clinical study that after denosumab injection, serum osteoclast markers (TRAcP5b) changed synchronously with CTX-1, but the BMD at all skeletal sites remained low. Similarly, in this study, the evaluation of OBs/OCs numbers was based on bone tissue staining, while CTX-1 was assessed using serological markers, which may lead to inconsistencies in the changes observed between the two indicators. Moreover, serum CTX-1 levels may be affected by various factors, including characteristics of the animal models (such as sex, age, circadian rhythm, and diet) and specific details of the SC-EV intervention (such as dosage, frequency, and duration) [76, 77]. Among the three studies that analyzed OBs/OCs numbers and CTX-1, there were differences in the SC-EV intervention frequency and treatment duration, which may contribute to the inconsistency between OBs/OCs numbers and CTX-1. A previous study suggested that with age, the number and activity of osteoclasts may increase, leading to enhanced bone resorption and upregulation of CTX-1 levels [78]. Although CTX-1 is primarily released by osteoclasts, the contribution of non-osteoclast sources of CTX-1 should not be overlooked. In a mouse study, inhibition of RANKL was associated with an increase in osteoblast morphology and osteoclast precursors, which continued to circulate and differentiate into osteoclasts, potentially in a time-dependent manner [79]. Therefore, the OB and OC precursors in animal models following SC-EV injection may also depend on the injection frequency and duration, continuing to differentiate into osteoclasts, and subsequently affecting serum CTX-1 levels. However, due to the limited number of included studies and sample sizes, the microscopic effects of SC-EVs on animal models (including OBs/OCs numbers, MAR, and serum OCN and CTX-1 levels) should be interpreted with caution. Future studies should involve larger sample sizes and explore the effects of SC-EVs on osteoblasts and osteoclasts under standardized models and intervention protocols.

It is noteworthy that among the 25 studies included, only four reported safety data on the intervention of SC-EVs in osteoporotic animal models. Specifically, Gui et al. [32] performed histopathological examinations of the heart, liver, spleen, lungs, and kidneys using hematoxylin and eosin (HE) staining, and assessed serum levels of ALT, AST, CK, CR, and BUN to evaluate heart, liver, and kidney functions, with no significant organ toxicity observed. Huang et al. [34] similarly conducted histopathological examinations of the heart, liver, spleen, lungs, and kidneys using HE staining, and measured the levels of inflammatory cytokines IL-1β and TNF-α in serum using ELISA, finding no in vivo toxicity or significant inflammatory responses. Although Lee et al. [35] investigated the distribution of ADSC-EVs in bone tissue in vivo, no assessments of organ toxicity were conducted. In contrast, another study combined HE staining and biophotonic imaging analysis to comprehensively evaluate the in vivo toxicity and metabolism of SC-EVs, showing no significant organ toxicity. Furthermore, they observed the distribution of BMSC-EV-loaded nanoparticles in visceral organs after injection, finding that most nanoparticles were cleared by the liver, with small amounts present in the kidneys, spleen, lungs, and around the femur. BMSC-EVs administered intravenously showed a clearance rate exceeding 80% within 24 h, indicating excellent long-term circulation properties [49]. The lack of in vivo safety evaluations in most studies poses challenges for the clinical translation of SC-EV-based therapies for osteoporosis. A previous animal study demonstrated that intravenous administration of hUCMSC-EVs did not cause toxic reactions, with normal immune and hepatic-renal function tests [80]. In a few phase I clinical trials, EV administration was stable in circulation and had low toxicity [81, 82], but clinical studies on the biodistribution of EVs are limited, and more reliable clinical safety data are needed in the future.

### Potential mechanisms of SC-EVs in regulating bone homeostasis

The dynamic balance between osteoblasts and osteoclasts maintains normal bone homeostasis and microenvironment, ensuring bone density and strength remain within the normal range [83]. However, in osteoporosis patients, enhanced bone resorption caused by overactive osteoclasts disrupts this balance, initiating pathological bone remodeling processes [83, 84]. Currently, the main strategy of SC-EVs in treating osteoporosis involves promoting osteoblast activity while inhibiting osteoclast activity to improve or reverse pathological bone remodeling. Analysis of general bone parameters revealed that SC-EVs treatment improved bone mass and strength in osteoporosis models, enhancing the biomechanical properties of bones. At the cellular level, SC-EVs may help regulate the bone microenvironment by promoting osteoblast proliferation and enhancing bone mineralization, while concurrently reducing osteoclast numbers. These effects align with the observed improvements in bone microarchitecture. Previous studies have demonstrated the effects of SC-EVs on osteoblast and osteoclast function. For instance, EVs derived from hADSCs have shown enhanced pro-osteogenic differentiation in vitro, accompanied by upregulation of ALP and RUNX2 [85]. hUCMSCs-EVs have been proven effective in inhibiting macrophage differentiation into osteoclasts, reducing bone resorption in osteoporotic mice [33]. Additionally, some EVs can simultaneously regulate both osteoblasts and osteoclasts, thereby maintaining bone homeostasis. For example, Lee et al. [35] found that ASC-EVs could promote the migration of BMSCs and inhibit osteoclast differentiation, facilitating bone remodeling. Interestingly, we observed that serum OCN, a bone turnover marker, was significantly upregulated in the SC-EVs treatment group, further confirming the potential regulatory role of SC-EVs in bone homeostasis.

Mechanistically, several key signaling pathways are crucial for SC-EVs to regulate the balance between bone formation and resorption, including the WNT/β-catenin, MAPK, PI3K/Akt, Hippo, and Smad pathways. For example, Zuo et al. [86] found that BMSC-EVs can mitigate radiation-induced bone loss by upregulating β-catenin expression, restoring the balance between osteogenic and adipogenic differentiation. Another study revealed that co-culturing BMSC-EVs with osteoblasts promoted cell proliferation, which was accompanied by the expression of key proteins in the MAPK signaling pathway [87]. Additionally, BMSC-EVs have been shown to promote bone formation in ovariectomy-induced osteoporotic rats via the Hippo signaling pathway [36]. BMSC-EVs delivering miR-22-3p have been proven to promote osteoblast differentiation by inhibiting the MYC/PI3K/AKT signaling axis [88]. Nakao et al. [89] reported that MSC-EVs derived from gingival tissue (GMSC-EVs) target the Wnt5a-mediated RANKL pathway, thereby suppressing osteoclast activity and reducing osteoclast numbers. Moreover, Xu et al. [90] demonstrated that MSC-EVs from aged rats, enriched in miR-128-3p, inhibit osteogenic differentiation by directly acting on Smad5.

**Fig. 13:**
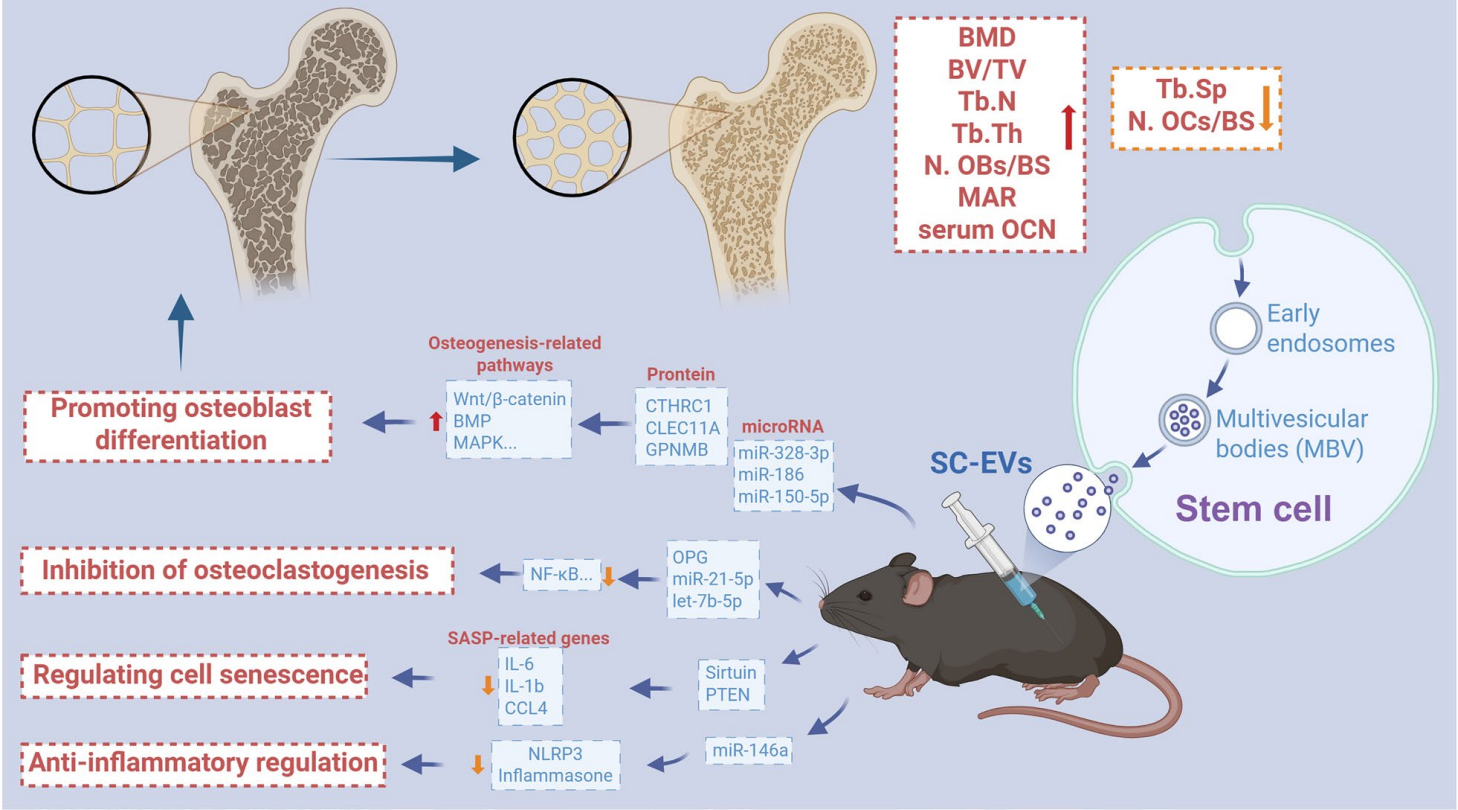
Potential Mechanisms of SC-EVs in the Treatment of Osteoporosis. SC-EVs originate from multivesicular bodies (MVBs) and, upon secretion, exert therapeutic effects on osteoporosis through multiple coordinated mechanisms, including promoting osteoblast differentiation, inhibiting osteoclastogenesis, regulating cellular senescence, and modulating anti-inflammatory responses

Based on current literature [29–53], SC-EVs may improve osteoporosis through a multi-target synergistic mechanism (Fig. 13). The specific mechanisms include: (1) Promotion of osteoblast differentiation: SC-EVs deliver proteins such as CTHRC1, CLEC11A, and GPNMB, as well as microRNAs like miR-328-3p and miR-186, to activate osteogenesis-related signaling pathways including Wnt/β-catenin, BMP, and AMPK, thereby enhancing bone formation and alleviating osteoporosis. (2) Inhibition of osteoclastogenesis: SC-EVs carry molecules such as OPG, miR-21-5p, and let-7b-5p that suppress the NF-κB signaling pathway, thereby reducing bone resorption. (3) Regulation of cellular senescence: SC-EVs can upregulate anti-aging genes via the Sirtuin and PTEN pathways, thereby restoring the function of bone marrow mesenchymal stem cells (BMSCs). (4) Anti-inflammatory modulation: SC-EVs mitigate the inflammatory microenvironment of osteoporosis by delivering miR-146a and inhibiting activation of the NLRP3 inflammasome. Notably, the Wnt signaling pathway serves as a convergence point for multiple mechanisms, and the miRNA and protein components within EVs form a cascading regulatory network that collectively restores bone metabolic homeostasis in osteoporosis. Although the potential mechanisms of SC-EVs in treating osteoporosis are being extensively studied, the current evidence remains limited. Further in-depth research on mechanisms and safety is needed before clinical translation can be achieved.

### Limitations and prospects

Our meta-analysis has several limitations. First, although significant therapeutic effects of SC-EVs on osteoporosis were observed, most of the included studies had unclear risks of bias, and there was considerable heterogeneity among the characteristics of animal models and SC-EVs. These factors necessitate a more cautious and objective interpretation of the efficacy of SC-EVs. Secondly, the analysis results exhibited varying degrees of heterogeneity and publication bias. Future studies should give adequate consideration to negative or null findings to ensure the objectivity of the conclusions. Moreover, the osteoporotic animal models used in the included studies were rats and mice, which may not fully align with the most suitable in vivo models for humans. Given this common limitation of preclinical studies, future research on SC-EVs should focus on animal models with genetic profiles more closely resembling humans. Finally, the majority of the included studies did not assess safety parameters (such as liver and kidney function), as well as in vivo distribution and metabolism, following SC-EV treatment. This represents a crucial step before future clinical translation.

Although current preclinical studies on SC-EV therapy for osteoporosis have demonstrated promising efficacy, several challenges remain for clinical translation. Firstly, variations in the source, isolation, purification, characterization, and intervention protocols of SC-EVs may influence therapeutic outcomes. In future preclinical studies, the standardization of the entire process from SC-EV preparation to administration should be achieved to enhance the consistency of therapeutic outcomes. Secondly, the in vivo biodistribution of SC-EVs may be affected by the route of administration; the use of carrier materials or sustained-release systems, such as hydrogels or nanoparticles, represents a promising direction for future development. Furthermore, while current studies suggest that EVs demonstrate favorable safety profiles in early-stage clinical trials, such as aerosolized MSC-EVs [91] and BMSC-EVs [92], long-term safety data and large-scale evaluations are still lacking. Therefore, rigorous and standardized clinical trials are essential to confirm the efficacy and safety of SC-EVs and to facilitate their successful clinical translation.

## Conclusions

This meta-analysis comprehensively compared the efficacy of SC-EVs in osteoporotic animal models across three aspects: bone analysis parameters, histological quantification, and serological indicators. Compared to placebo, SC-EVs demonstrated beneficial efficacy, particularly in increasing bone strength and mass (both cortical and trabecular bone), promoting osteoblast proliferation, enhancing bone mineralization, reducing osteoclast numbers, and upregulating serum OCN. These findings lay the foundation for further investigation into the therapeutic potential of SC-EVs for osteoporosis. However, researchers must address two critical issues: first, standardizing specific protocols for SC-EVs treatment in osteoporosis to reduce inter-study heterogeneity and improve the reliability of results; second, conducting necessary safety assessments to ensure its clinical translation potential.

## Supplementary Information

Supplementary material 1.

Supplementary material 2.

## Data Availability

All data relevant to the study are included in the article.

## Abbreviations

ALP: Alkaline phosphatase
ASC: Adipose-derived stem cells
BMD: Bone mineral density
BMSCs: Bone marrow mesenchymal stem cells
BV: Bone volume
BV/TV: Bone volume/total volume
Ct.Th: Cortical thickness
CTX1: C-Terminal Telopeptide of Type I Collagen
DPSC: Dental pulp stem cell
EV: Extracellular vesicle
GelMA: Gelatin methacryloyl
GMSC: Gingival mesenchymal stem cells
HAMA: Hyaluronic acid methacryloyl
hADSC: Human adipose-derived stem cells
hESC: Human embryonic stem cell
hUSC: Human urine-derived stem cell
hUCMSC: Human umbilical cord mesenchymal stem cell
hiPSC-MSC: Mesenchymal stem cells derived from human induced pluripotent stem cell
MAR: Mineral apposition rate
MVB: Multivesicular bodies
MHC: Major histocompatibility complex
MSC: Mesenchymal stem cell
N. OBs/BS: Number of osteoblasts per bone surface
N. OCs/BS: Number of osteoclasts per bone surface
OCN: Osteocalcin
PI3K: Phosphoinositide 3-kinase
PRISMA: Preferred Reporting Items for Systematic Reviews and Meta-Analyses
PROSPERO: Prospective Register of Systematic Reviews
RANKL: Receptor activator of nuclear factor kappa-β ligand
RUNX2: Runt-related transcription factor 2
SC-EVs: Stem cell-derived extracellular vesicles
SD: Sprague Dawley
SMD: Standardized mean difference
STS: Staurosporine
SYRCLE: Systematic Review Centre for Laboratory Animal Experimentation
TFF: Tangential flow filtration
Tb.BV/TV: Trabecular bone volume/total volume
Tb.N: Trabecular number
Tb.Sp: Trabecular separation
Tb.Th: Trabecular thickness
WJ-MSC: Wharton’s jelly-mesenchymal stem cell
