# Supplementary material for: Unlocking the potential of stem cell-derived extracellular vesicles in osteoporosis therapy: a systematic review and meta-analysis of preclinical studies
Source: J Transl Med. 2025 Jun 18;23:683. doi: 10.1186/s12967-025-06654-5 (PMC12178078; doi:10.1186/s12967-025-06654-5)
Supplement: Supplementary file 1 — Supplementary material 1. [file 12967_2025_6654_MOESM1_ESM.docx]

**Supplement** [**Materials**](D:/%E8%BD%AF%E4%BB%B6/360%E6%B8%B8%E8%A7%88%E5%99%A8/Youdao/Dict/8.9.9.0/resultui/html/index.html#/javascript:;)

**The Detailed Search Strategy:**

**Pubmed Cut-off retrieval date: November 10, 2024**

#1: "Extracellular Vesicles"[Mesh]

Results: 32,438

#2: (Extracellular Vesicle[Title/Abstract]) OR (Vesicle, Extracellular[Title/Abstract]) OR (Vesicles, Extracellular[Title/Abstract]) OR (Exovesicles[Title/Abstract]) OR (Exovesicle[Title/Abstract]) OR (Apoptotic Bodies[Title/Abstract]) OR (Apoptotic Body[Title/Abstract]) OR (Bodies, Apoptotic[Title/Abstract]) OR (Body, Apoptotic[Title/Abstract]) OR (Exosomes[Title/Abstract]) OR (Endosomes[Title/Abstract]) OR (Secretory Vesicles[Title/Abstract]) OR (Cell-Derived Microparticles[Title/Abstract]) OR (Exosome Multienzyme Ribonuclease Complex[Title/Abstract])

Results: 56,132

#3: #1 OR #2

Results: 68,510

#4: "Osteoporosis"[Mesh]

Results: 65,924

#5: (Osteoporoses[Title/Abstract]) OR (Osteoporosis, Age-Related[Title/Abstract]) OR (Osteoporosis, Age Related[Title/Abstract]) OR (Age-Related Osteoporosis[Title/Abstract]) OR Age-Related Osteoporoses[Title/Abstract]) OR (Age Related Osteoporosis[Title/Abstract]) OR (Osteoporoses, Age-Related[Title/Abstract]) OR (Bone Loss, Age-Related[Title/Abstract]) OR (Age-Related Bone Loss[Title/Abstract]) OR (Age-Related Bone Losses[Title/Abstract]) OR (Bone Loss, Age Related[Title/Abstract]) OR (Bone Losses, Age-Related[Title/Abstract]) OR (Osteoporosis, Senile[Title/Abstract]) OR (Osteoporoses, Senile[Title/Abstract]) OR (Senile Osteoporoses[Title/Abstract]) OR (Senile Osteoporosis[Title/Abstract]) OR (Osteoporosis, Involutional[Title/Abstract]) OR (Osteoporosis, Post-Traumatic[Title/Abstract]) OR (Osteoporosis, Post Traumatic[Title/Abstract]) OR (Post-Traumatic Osteoporoses[Title/Abstract]) OR (Post-Traumatic Osteoporosis[Title/Abstract])

Results: 4,195

#6: #4 OR #5

Results: 67,728

#7: #3 AND #6

(("Extracellular Vesicles"[Mesh]) OR ((Extracellular Vesicle[Title/Abstract]) OR (Vesicle, Extracellular[Title/Abstract]) OR (Vesicles, Extracellular[Title/Abstract]) OR (Exovesicles[Title/Abstract]) OR (Exovesicle[Title/Abstract]) OR (Apoptotic Bodies[Title/Abstract]) OR (Apoptotic Body[Title/Abstract]) OR (Bodies, Apoptotic[Title/Abstract]) OR (Body, Apoptotic[Title/Abstract]) OR (Exosomes[Title/Abstract]) OR (Endosomes[Title/Abstract]) OR (Secretory Vesicles[Title/Abstract]) OR (Cell-Derived Microparticles[Title/Abstract]) OR (Exosome Multienzyme Ribonuclease Complex[Title/Abstract]))) AND (("Osteoporosis"[Mesh]) OR ((Osteoporoses[Title/Abstract]) OR (Osteoporosis, Age-Related[Title/Abstract]) OR (Osteoporosis, Age Related[Title/Abstract]) OR (Age-Related Osteoporosis[Title/Abstract]) OR Age-Related Osteoporoses[Title/Abstract]) OR (Age Related Osteoporosis[Title/Abstract]) OR (Osteoporoses, Age-Related[Title/Abstract]) OR (Bone Loss, Age-Related[Title/Abstract]) OR (Age-Related Bone Loss[Title/Abstract]) OR (Age-Related Bone Losses[Title/Abstract]) OR (Bone Loss, Age Related[Title/Abstract]) OR (Bone Losses, Age-Related[Title/Abstract]) OR (Osteoporosis, Senile[Title/Abstract]) OR (Osteoporoses, Senile[Title/Abstract]) OR (Senile Osteoporoses[Title/Abstract]) OR (Senile Osteoporosis[Title/Abstract]) OR (Osteoporosis, Involutional[Title/Abstract]) OR (Osteoporosis, Post-Traumatic[Title/Abstract]) OR (Osteoporosis, Post Traumatic[Title/Abstract]) OR (Post-Traumatic Osteoporoses[Title/Abstract]) OR (Post-Traumatic Osteoporosis[Title/Abstract])))

**Results: 155**

**Cochrane Library Cut-off retrieval date: November 10, 2024**

#1: MeSH descriptor: [Extracellular Vesicles] explode all trees

Results: 202

#2: (Extracellular Vesicle):ab,ti,kw OR (Vesicle, Extracellular):ab,ti,kw OR (Vesicles, Extracellular):ab,ti,kw OR (Exovesicles):ab,ti,kw OR (Exovesicle):ab,ti,kw OR (Apoptotic Bodies):ab,ti,kw OR (Apoptotic Body):ab,ti,kw OR (Bodies, Apoptotic):ab,ti,kw OR (Body, Apoptotic):ab,ti,kw OR (Exosomes):ab,ti,kw OR (Endosomes):ab,ti,kw OR (Secretory Vesicles):ab,ti,kw OR (Cell-Derived Microparticles):ab,ti,kw OR (Exosome Multienzyme Ribonuclease Complex):ab,ti,kw

Results: 807

#3: #1 OR #2

Results: 807

#4: MeSH descriptor: [Osteoporosis] explode all trees

Results: 5,539

#5: (Osteoporoses):ab,ti,kw OR (Osteoporosis, Age-Related):ab,ti,kw OR (Osteoporosis, Age Related):ab,ti,kw OR (Age-Related Osteoporosis):ab,ti,kw OR (Age-Related Osteoporoses):ab,ti,kw OR (Age Related Osteoporosis):ab,ti,kw OR (Osteoporoses, Age-Related):ab,ti,kw OR (Bone Loss, Age-Related):ab,ti,kw OR (Age-Related Bone Loss):ab,ti,kw OR (Age-Related Bone Losses):ab,ti,kw OR (Bone Loss, Age Related):ab,ti,kw OR (Bone Losses, Age-Related):ab,ti,kw OR (Osteoporosis, Senile):ab,ti,kw OR (Osteoporoses, Senile):ab,ti,kw OR (Senile Osteoporoses):ab,ti,kw OR (Senile Osteoporosis):ab,ti,kw OR (Osteoporosis, Involutional):ab,ti,kw OR (Osteoporosis, Post-Traumatic):ab,ti,kw OR (Osteoporosis, Post Traumatic):ab,ti,kw OR (Post-Traumatic Osteoporoses):ab,ti,kw OR (Post-Traumatic Osteoporosis):ab,ti,kw

Results: 1,462

#6: #4 OR #5

Results: 6,597

#7: #3 AND #6

**Results: 2**

**Web of Science Cut-off retrieval date: November 10, 2024**

#1: TS=(Extracellular Vesicle OR Exovesicles OR Exovesicle OR Apoptotic Bodies OR Apoptotic Body OR Exosomes OR Endosomes OR Secretory Vesicles OR Cell-Derived Microparticles OR Exosome Multienzyme Ribonuclease Complex)

Results: 196,094

#2: TS=(Osteoporoses OR Age-Related Osteoporosis OR Age-Related Osteoporoses OR Age Related Osteoporosis OR Age-Related Bone Loss OR Age-Related Bone Losses OR Senile Osteoporoses OR Senile Osteoporosis OR Post-Traumatic Osteoporoses OR Post-Traumatic Osteoporosis)

Results: 37,402

#3: #1 AND #2

**Results: 227**

**Embase Cut-off retrieval date: November 10, 2024**

#1: 'extracellular vesicles'/exp

Results: 68,956

#2: 'Extracellular Vesicle':ab,ti,kw OR 'Vesicle, Extracellular':ab,ti,kw OR 'Vesicles, Extracellular':ab,ti,kw OR 'Exovesicles':ab,ti,kw OR 'Exovesicle':ab,ti,kw OR 'Apoptotic Bodies':ab,ti,kw OR 'Apoptotic Body':ab,ti,kw OR 'Bodies, Apoptotic':ab,ti,kw OR 'Body, Apoptotic':ab,ti,kw OR 'Exosomes':ab,ti,kw OR 'Endosomes':ab,ti,kw OR 'Secretory Vesicles':ab,ti,kw OR 'Cell-Derived Microparticles':ab,ti,kw OR 'Exosome Multienzyme Ribonuclease Complex':ab,ti,kw

Results: 71,293

#3: #1 OR #2

Results: 100,563

#4: 'Osteoporosis'/exp

Results: 166,005

#5: 'Osteoporoses':ab,ti,kw OR 'Osteoporosis, Age-Related':ab,ti,kw OR 'Osteoporosis, Age Related':ab,ti,kw OR 'Age-Related Osteoporosis':ab,ti,kw OR 'Age-Related Osteoporoses':ab,ti,kw OR 'Age Related Osteoporosis':ab,ti,kw OR 'Osteoporoses, Age-Related':ab,ti,kw OR 'Bone Loss, Age-Related':ab,ti,kw OR 'Age-Related Bone Loss':ab,ti,kw OR 'Age-Related Bone Losses':ab,ti,kw OR 'Bone Loss, Age Related':ab,ti,kw OR 'Bone Losses, Age-Related':ab,ti,kw OR 'Osteoporosis, Senile':ab,ti,kw OR 'Osteoporoses, Senile':ab,ti,kw OR 'Senile Osteoporoses':ab,ti,kw OR 'Senile Osteoporosis':ab,ti,kw OR 'Osteoporosis, Involutional':ab,ti,kw OR 'Osteoporosis, Post-Traumatic':ab,ti,kw OR 'Osteoporosis, Post Traumatic':ab,ti,kw OR 'Post Traumatic Osteoporoses':ab,ti,kw OR 'Post-Traumatic Osteoporosis':ab,ti,kw

Results: 2,499

#6: #4 OR #5

Results: 166,721

#7: #3 AND #6

**Results: 488**
