# Supplementary material for: Unlocking the potential of stem cell-derived extracellular vesicles in osteoporosis therapy: a systematic review and meta-analysis of preclinical studies
Source: J Transl Med. 2025 Jun 18;23:683. doi: 10.1186/s12967-025-06654-5 (PMC12178078; doi:10.1186/s12967-025-06654-5)
Supplement: Supplementary file 2 — Supplementary material 2. [file 12967_2025_6654_MOESM2_ESM.docx]

**Supplementary materials**

**Supplementary figures**

**
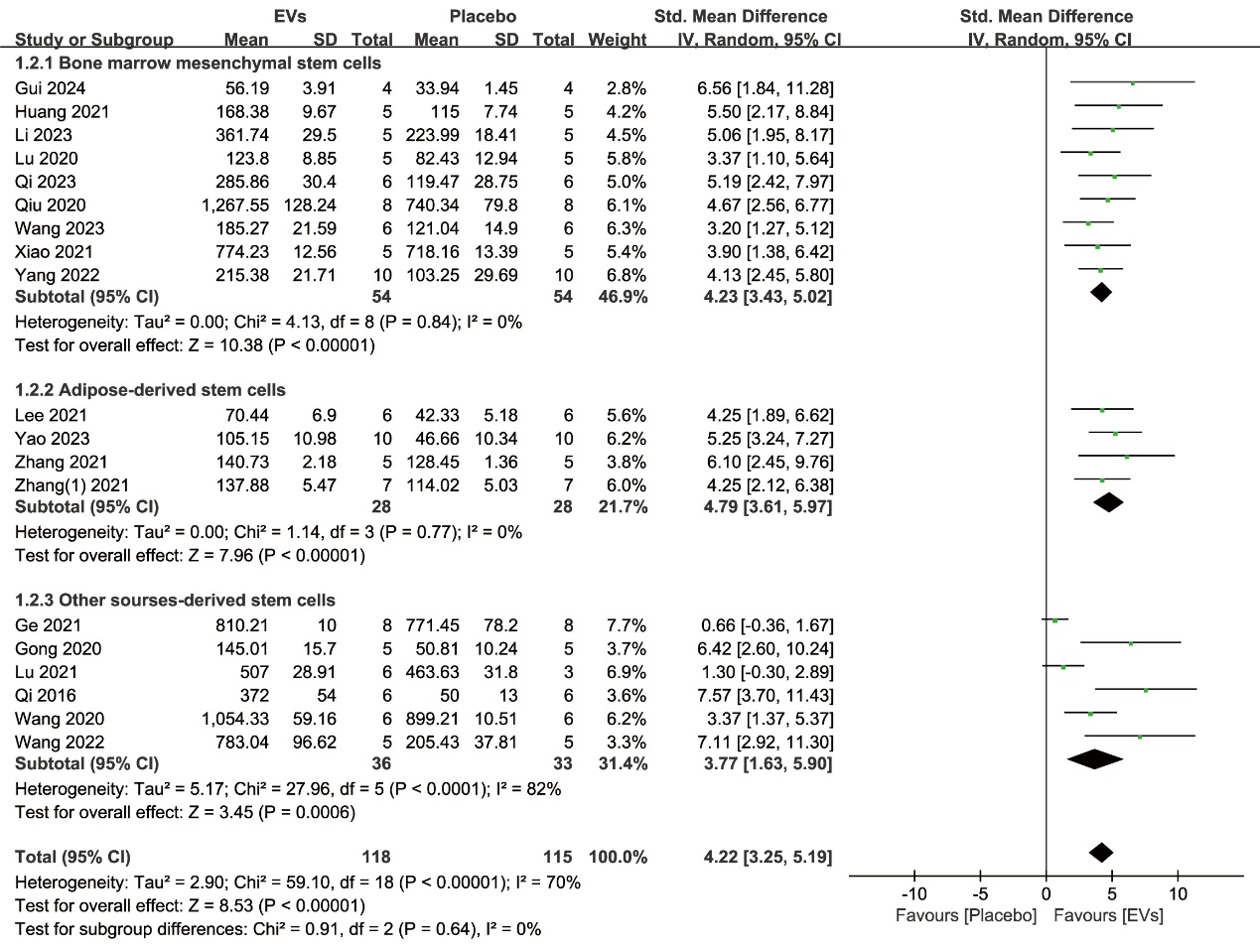
**

**Figure S1.** Subgroup analysis based on different SC-EVs sources for bone mineral density (BMD). Data are presented as standardized mean difference (SMD) with 95% confidence intervals (CI).


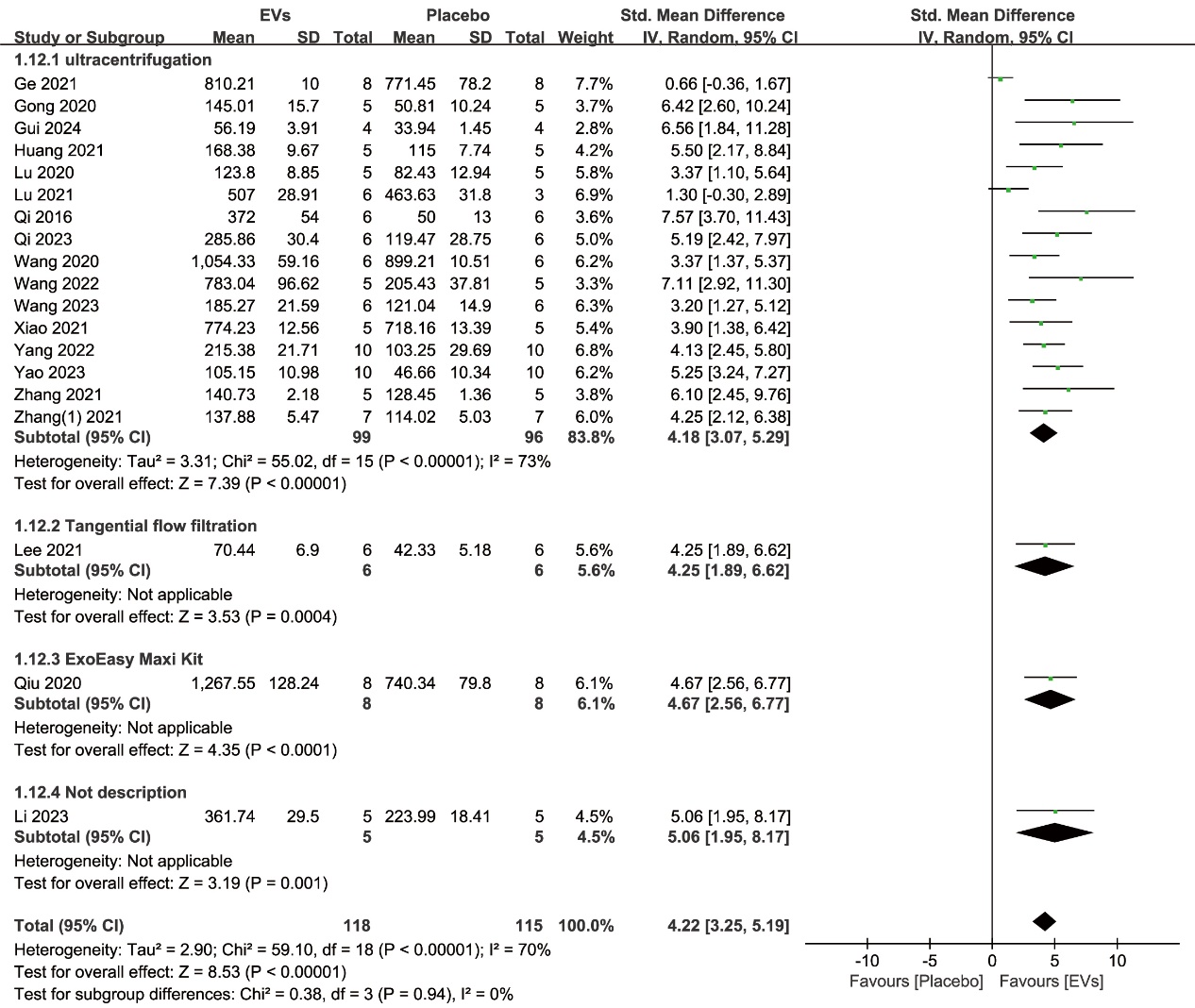


**Figure S2.** Subgroup analysis of bone mineral density (BMD) based on different SC-EVs isolation methods. Data are presented as standardized mean difference (SMD) with 95% confidence intervals (CI).


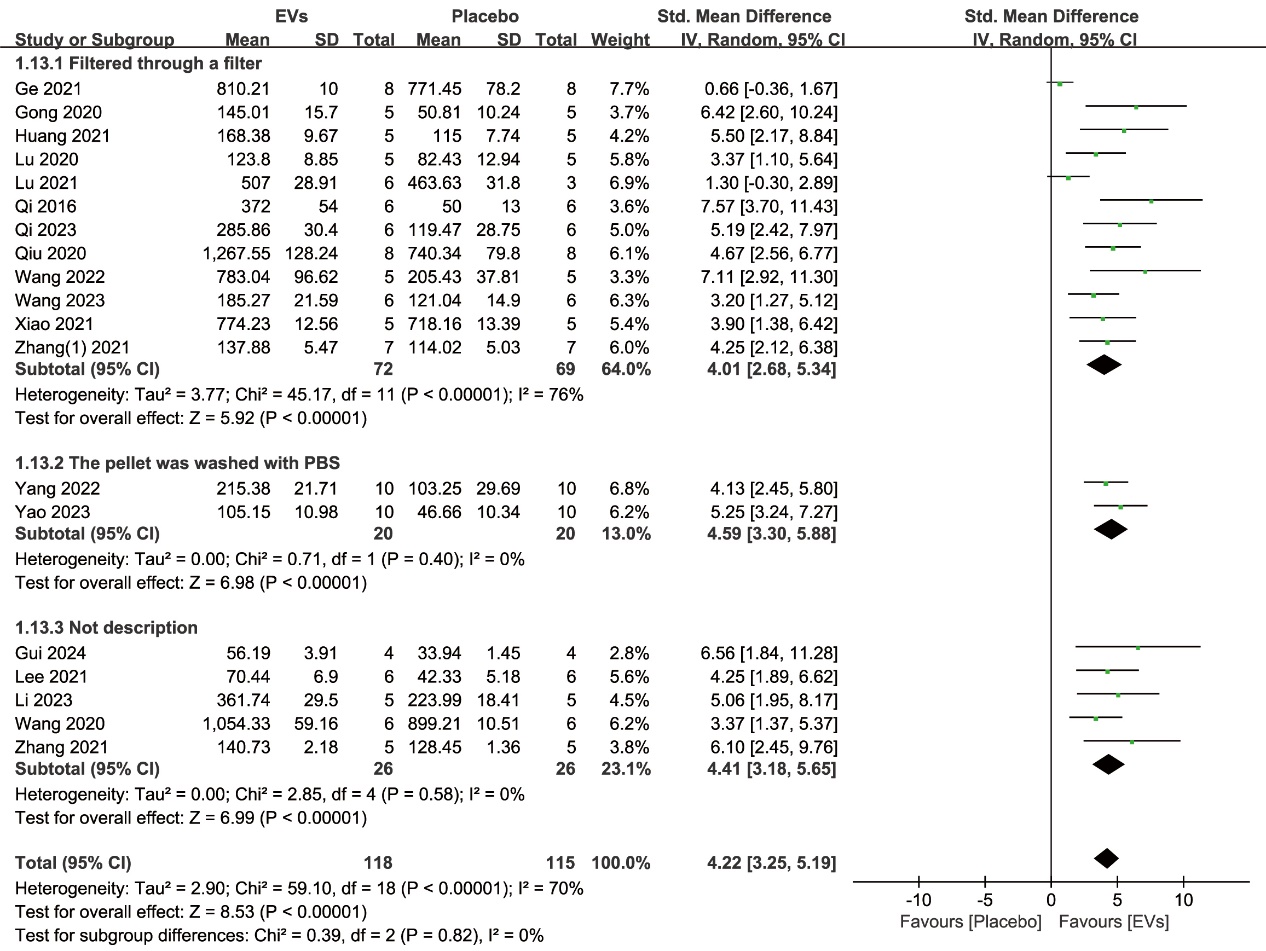


**Figure S3.** Subgroup analysis of bone mineral density (BMD) based on different SC-EV purification methods. Data are presented as standardized mean difference (SMD) with 95% confidence intervals (CI).


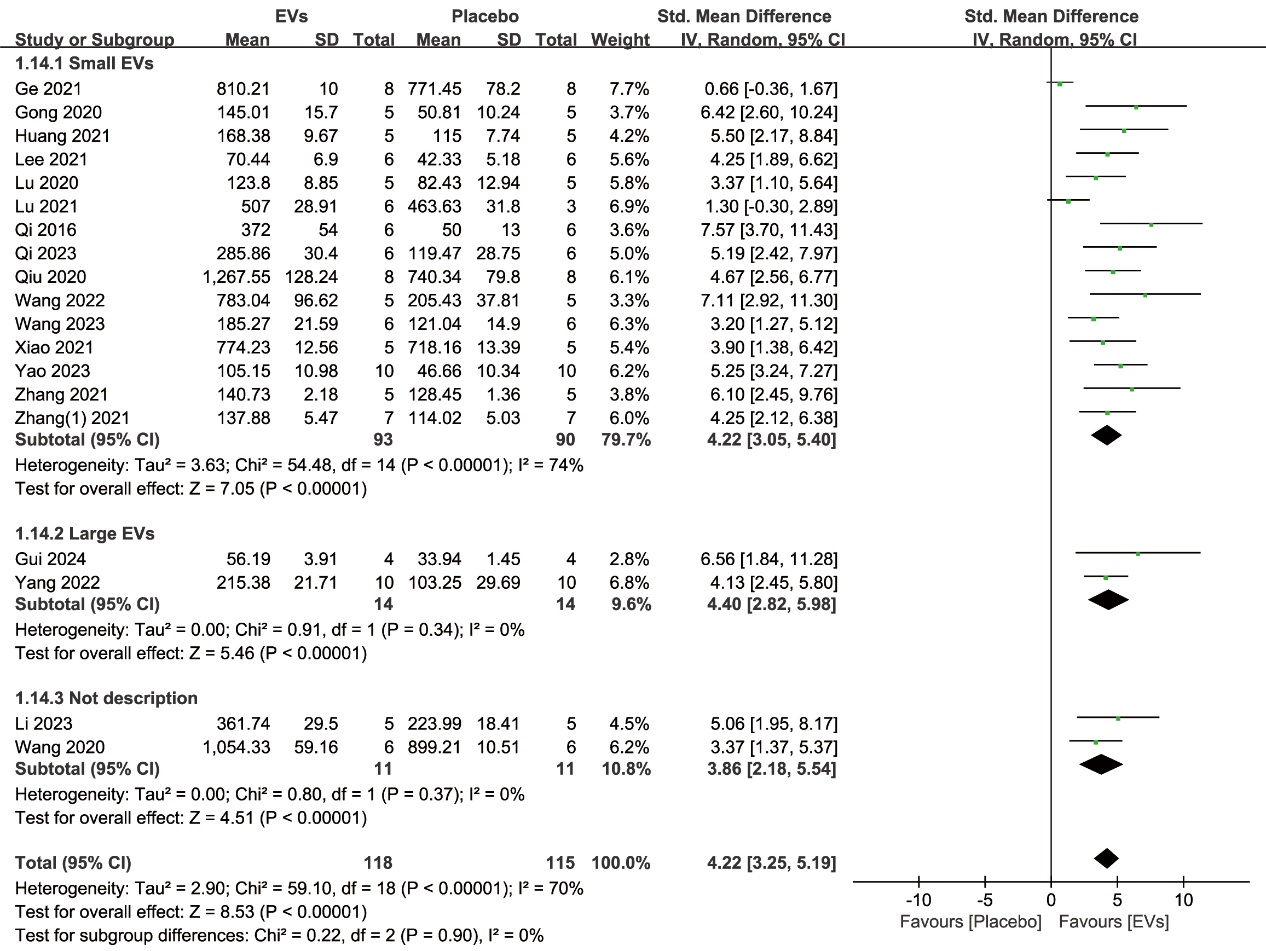


**Figure S4.** Subgroup analysis of bone mineral density (BMD) based on different SC-EV sizes. Data are presented as standardized mean difference (SMD) with 95% confidence intervals (CI).

**
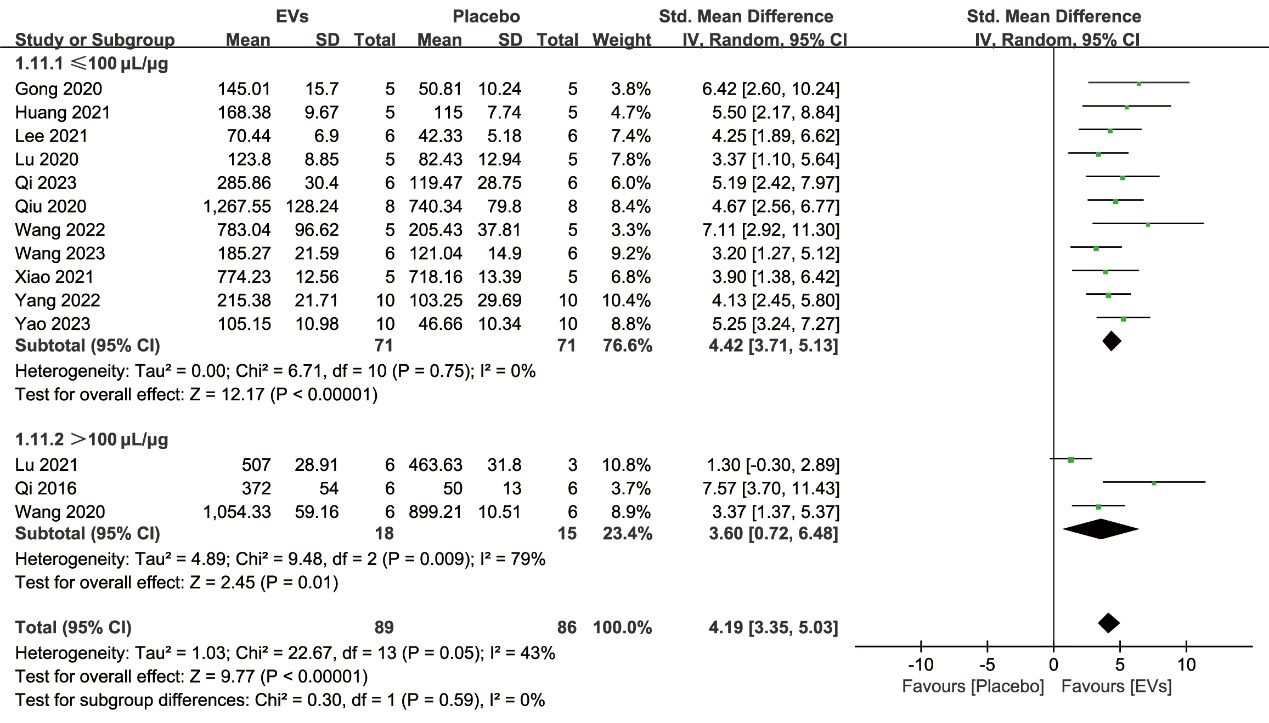
**

**Figure S5.** Subgroup analysis of Bone mineral density (BMD) based on different SC-EV intervention doses. Data are presented as standardized mean difference (SMD) with 95% confidence intervals (CI).


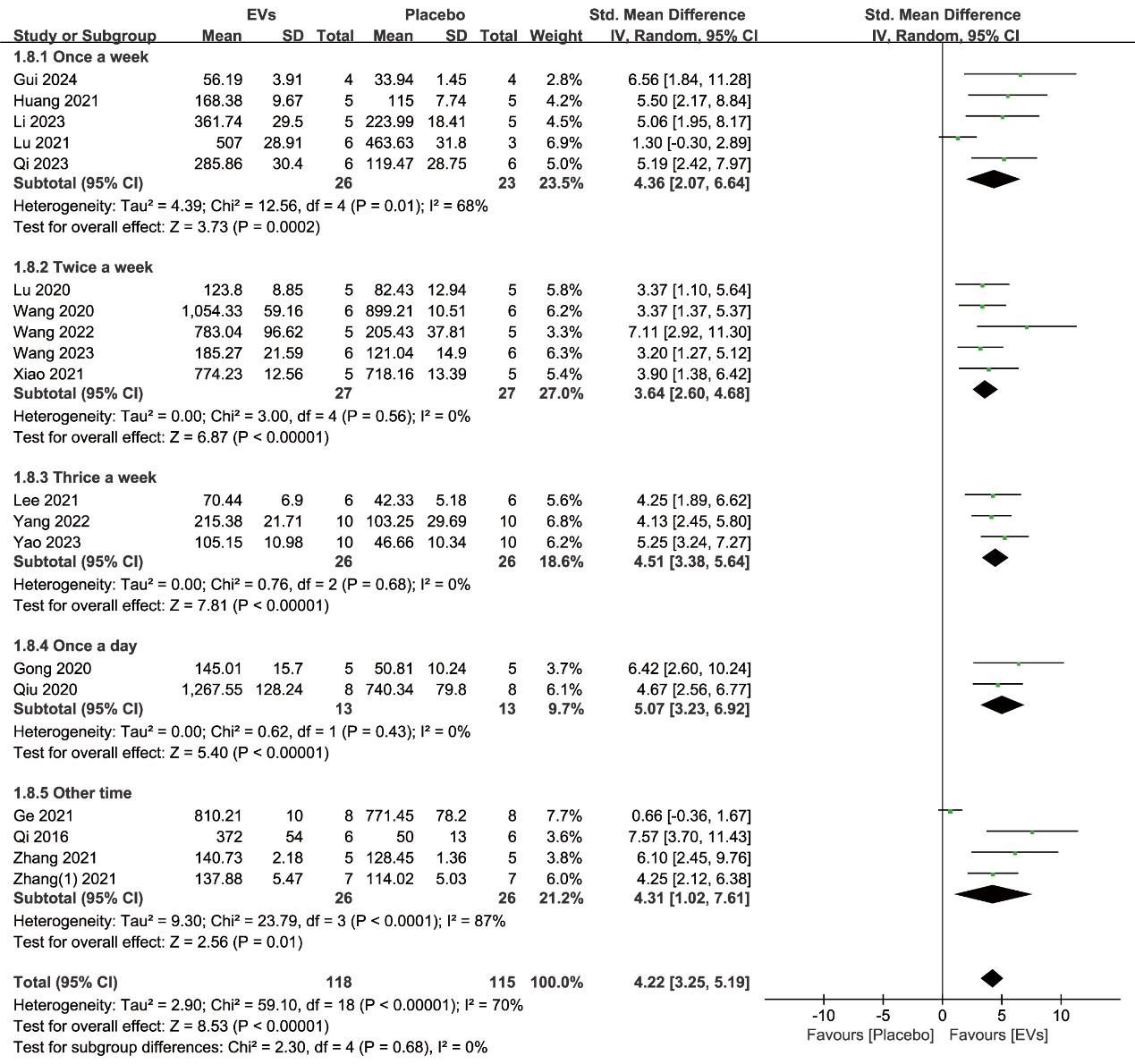


**Figure S6.** Subgroup analysis of bone mineral density (BMD) based on different administration frequencies. Data are presented as standardized mean difference (SMD) with 95% confidence intervals (CI).


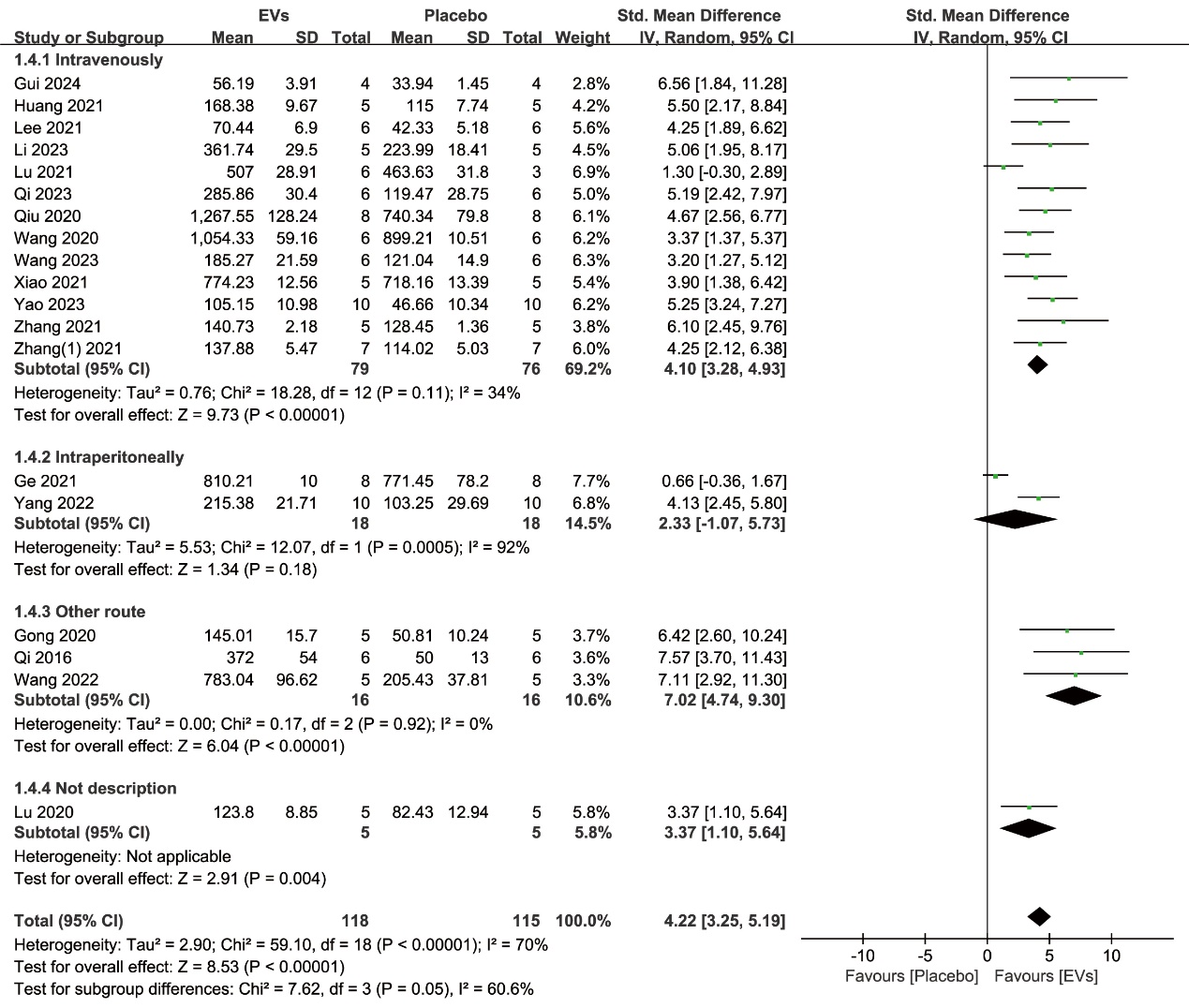


**Figure S7.** Subgroup analysis of bone mineral density (BMD) based on different administration routes. Data are presented as standardized mean difference (SMD) with 95% confidence intervals (CI).


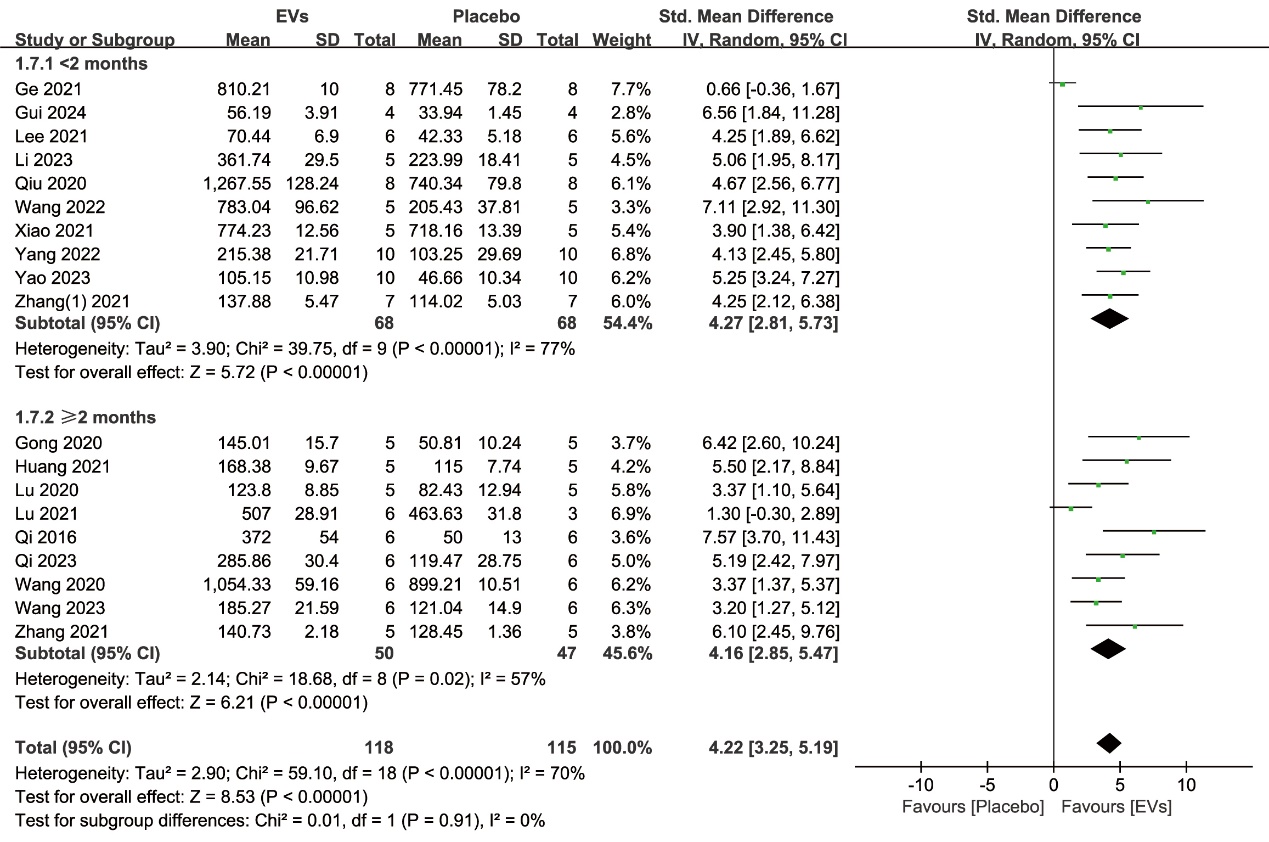


**Figure S8.** Subgroup analysis of bone mineral density (BMD) based on different treatment durations. Data are presented as standardized mean difference (SMD) with 95% confidence intervals (CI).

**
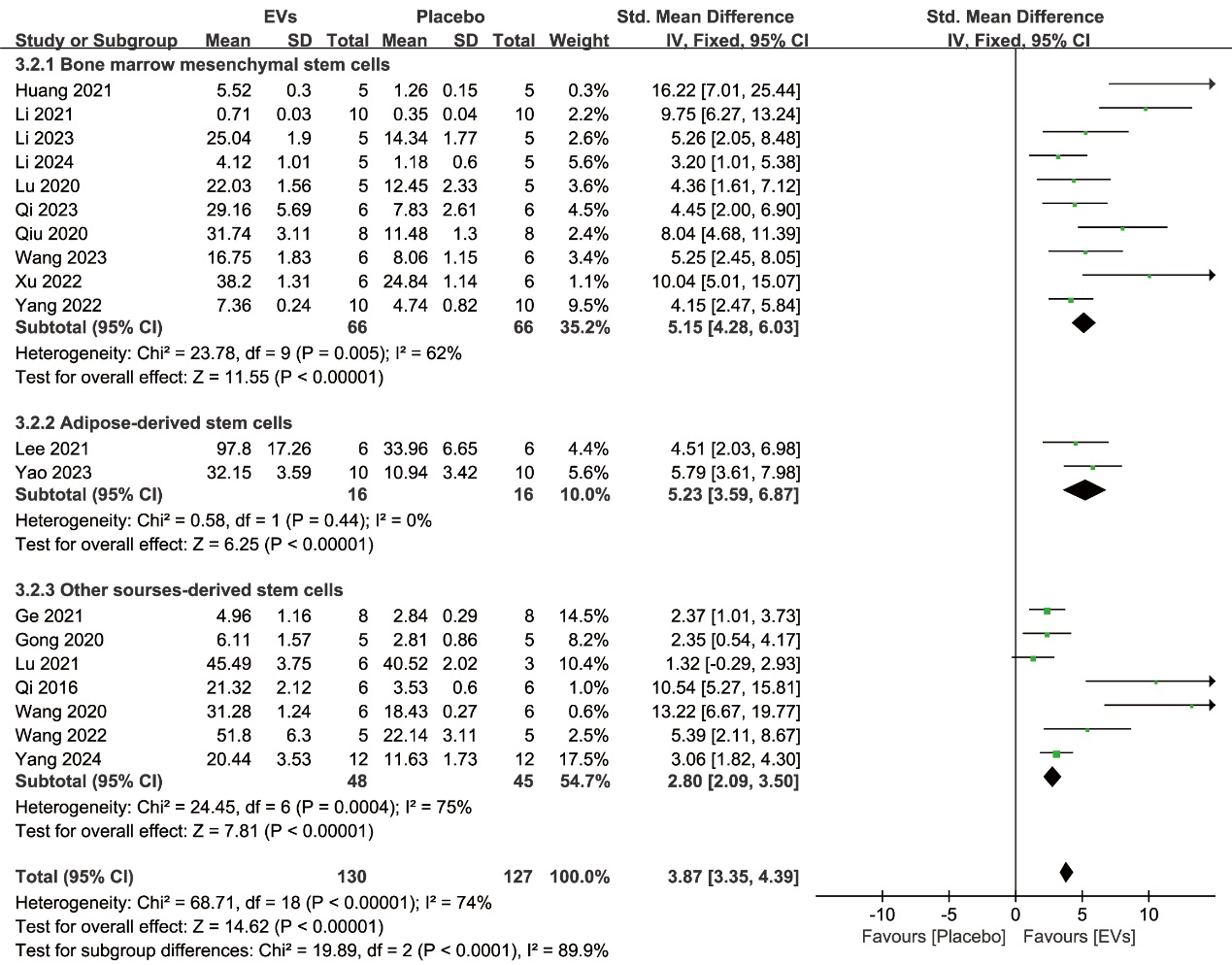
**

**Figure S9.** Subgroup analysis based on different SC-EVs sources for BV/TV. Data are presented as standardized mean difference (SMD) with 95% confidence intervals (CI).


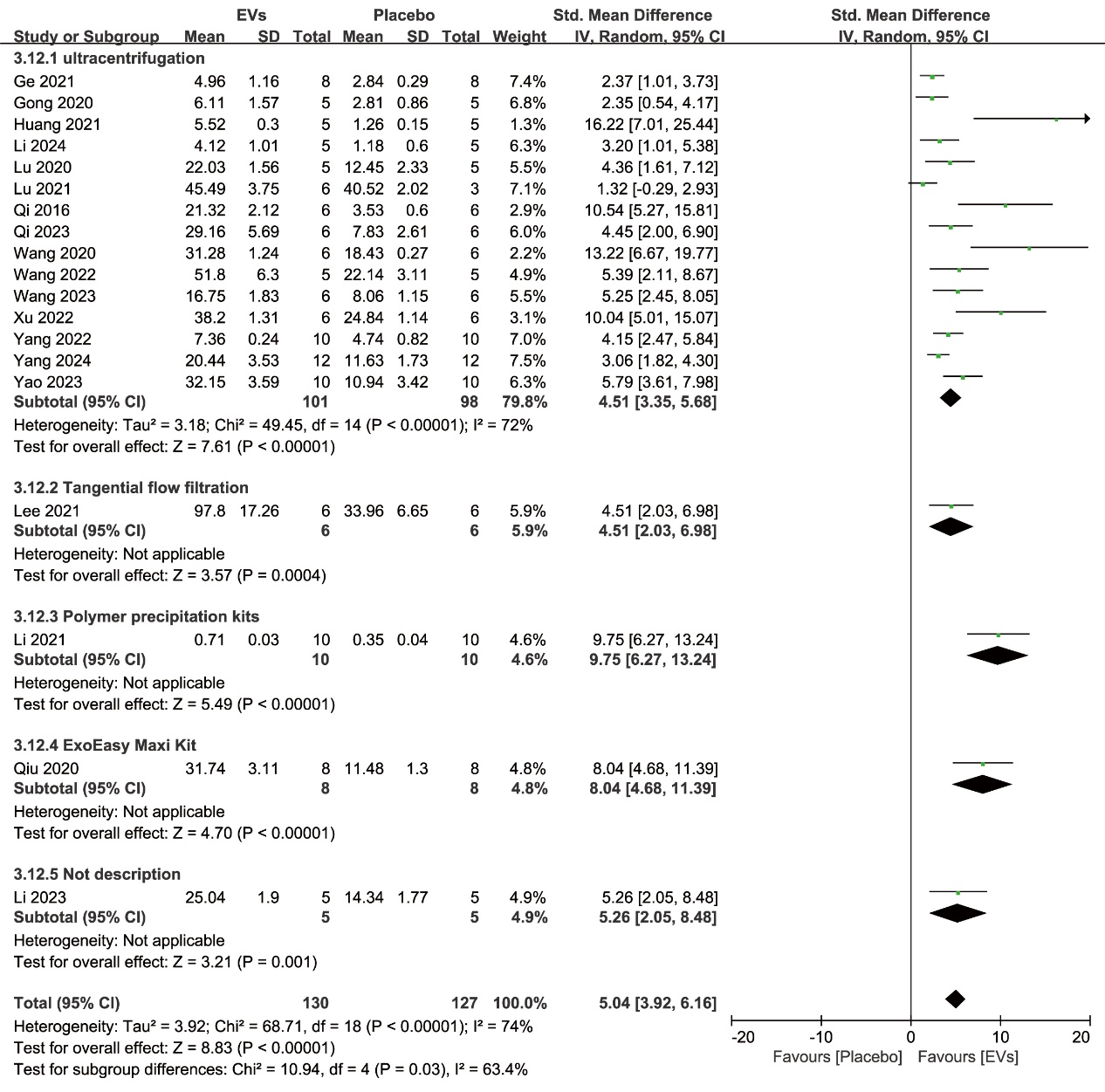


**Figure S10.** Subgroup analysis of BV/TV based on different SC-EVs isolation methods. Data are presented as standardized mean difference (SMD) with 95% confidence intervals (CI).


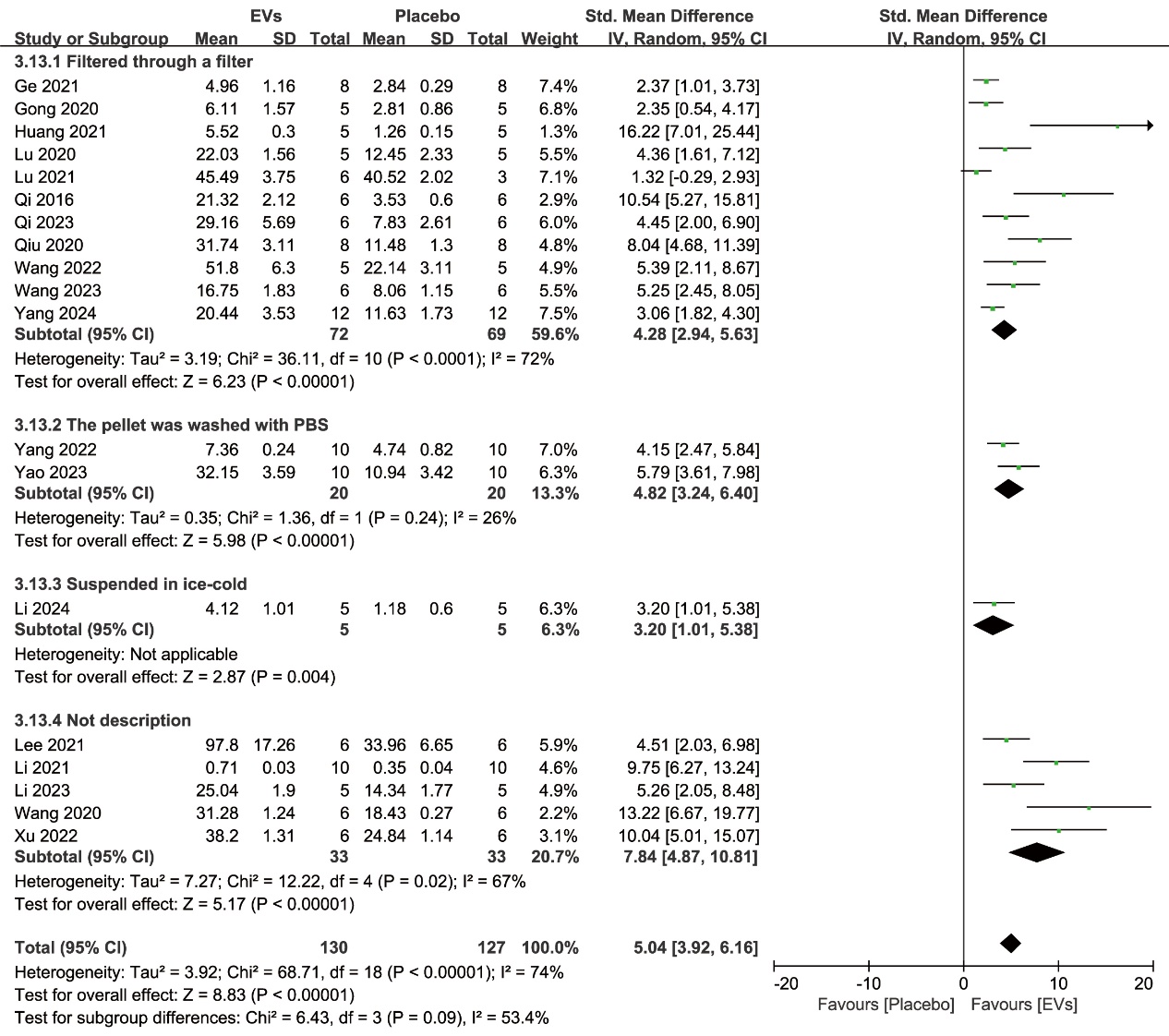


**Figure S11.** Subgroup analysis of BV/TV based on different SC-EVs purification methods. Data are presented as standardized mean difference (SMD) with 95% confidence intervals (CI).


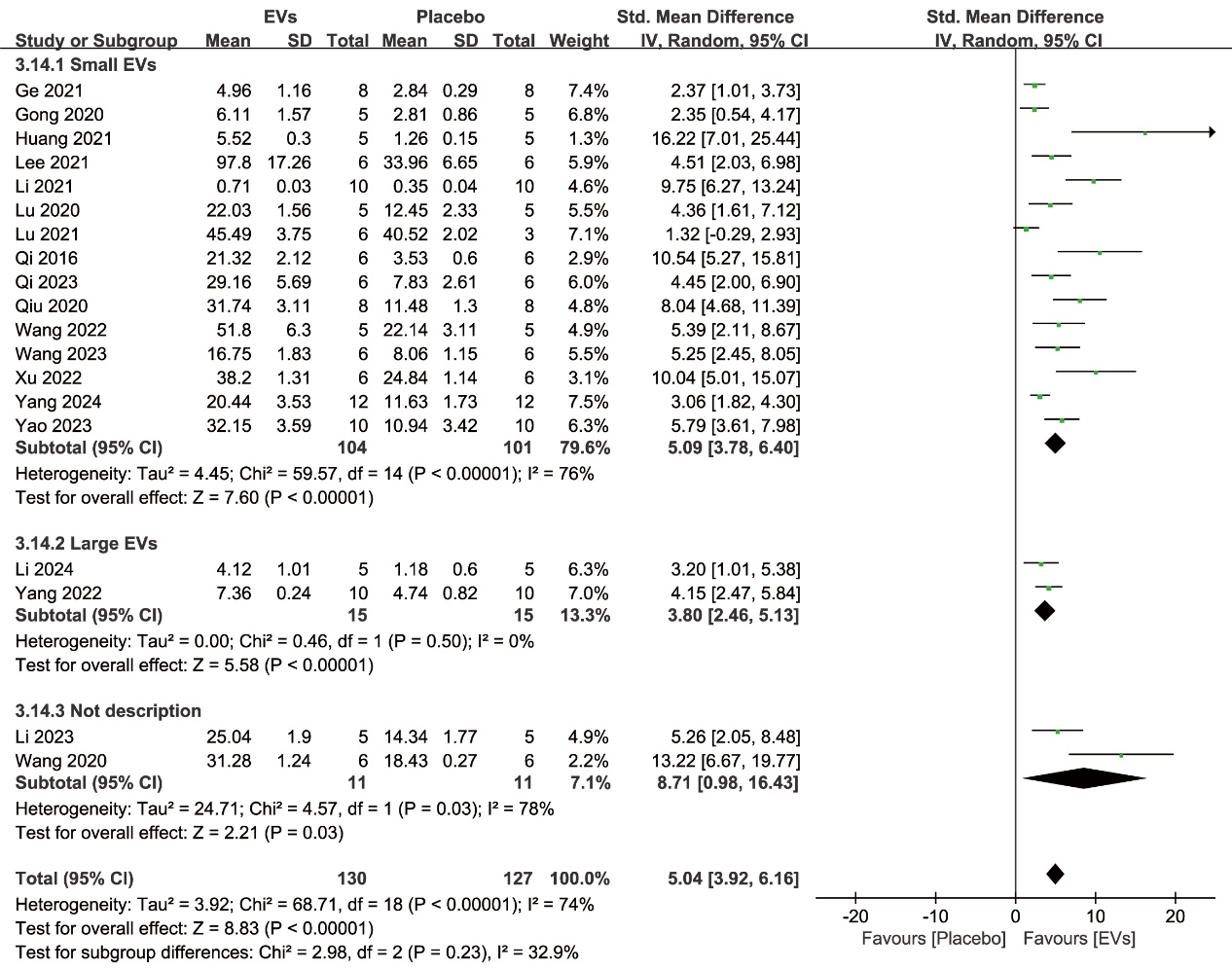


**Figure S12.** Subgroup analysis of BV/TV based on different SC-EV sizes. Data are presented as standardized mean difference (SMD) with 95% confidence intervals (CI).

**
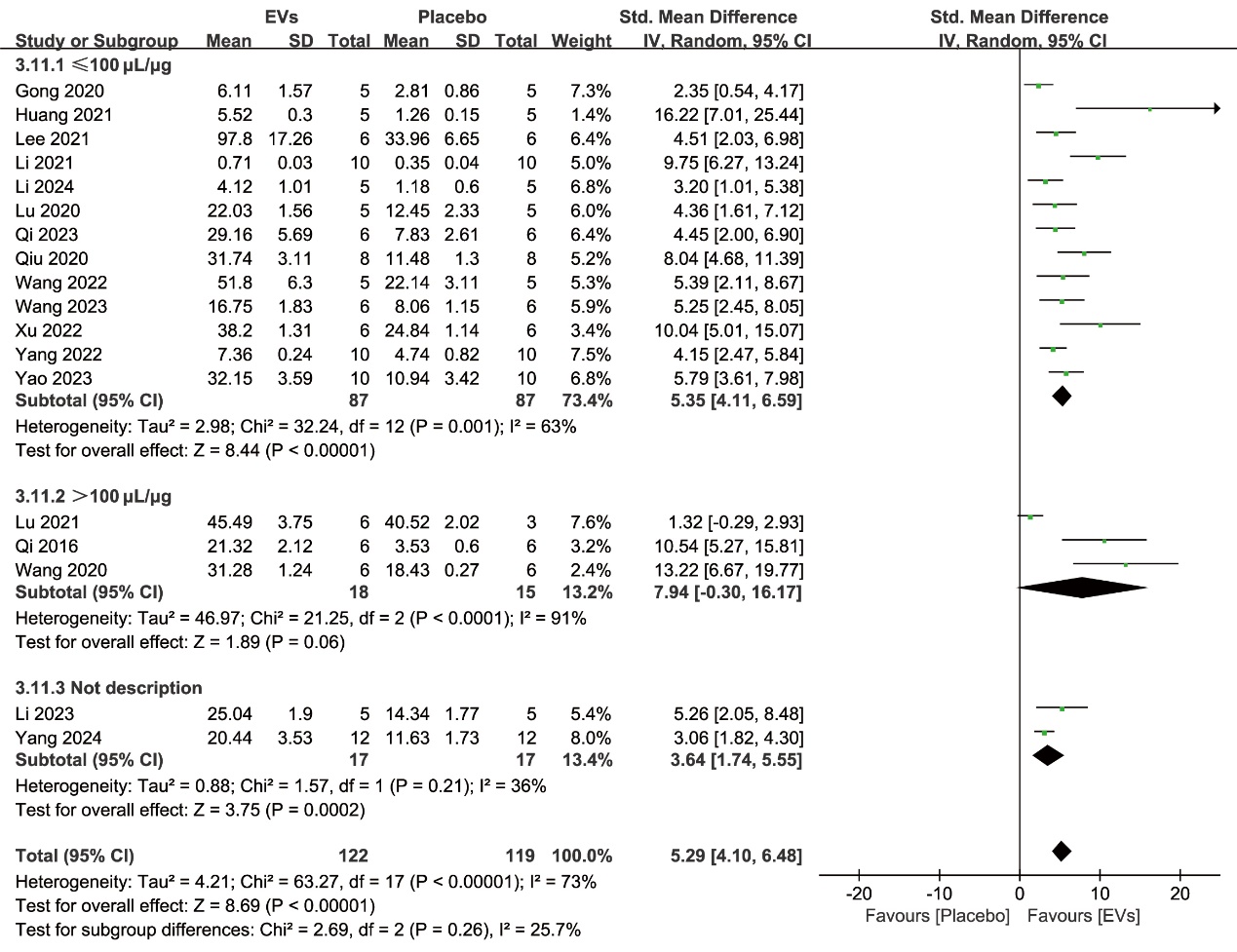
**

**Figure S13.** Subgroup analysis of BV/TV based on different SC-EV intervention doses. Data are presented as standardized mean difference (SMD) with 95% confidence intervals (CI).

**
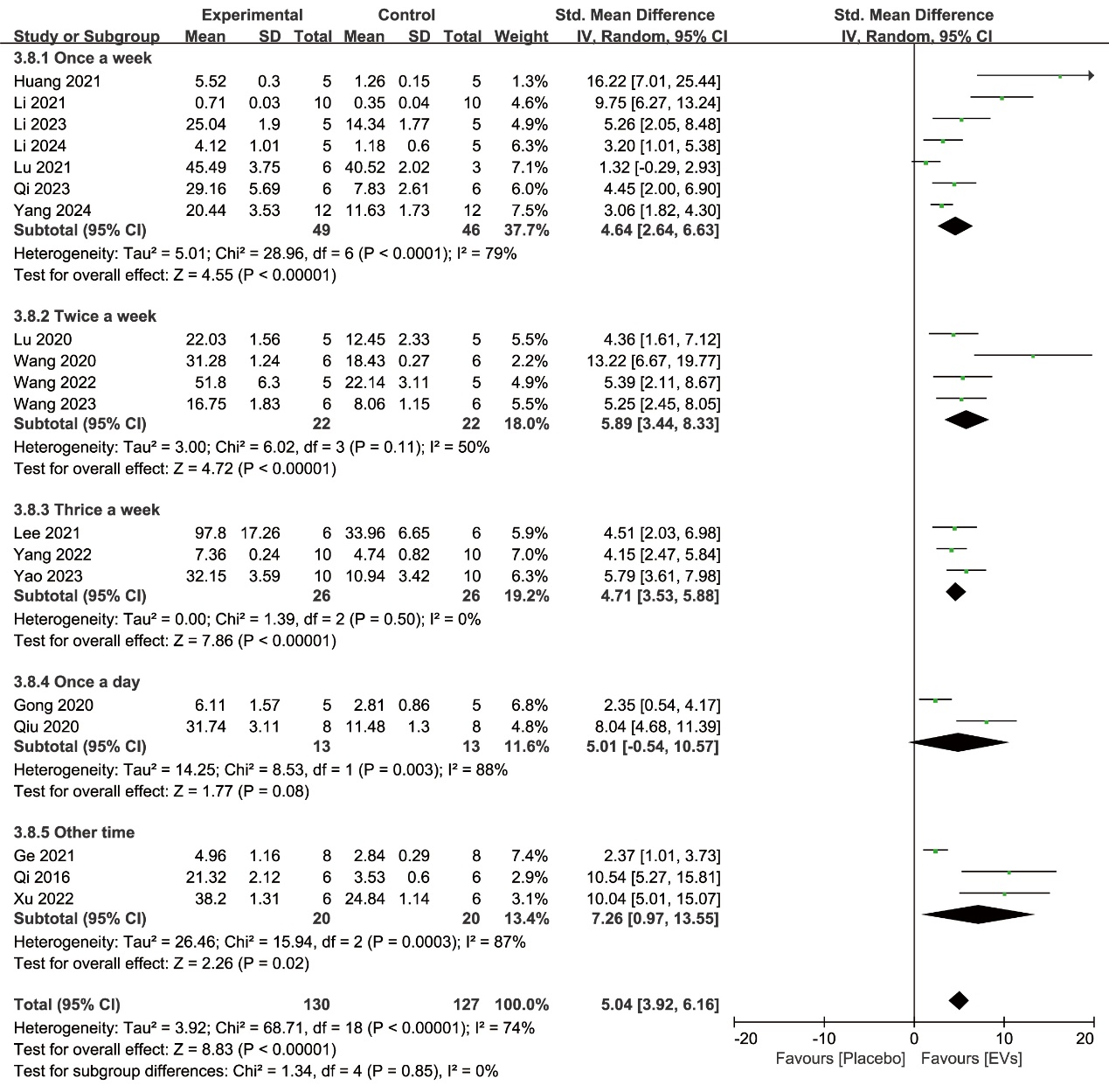
**

**Figure S14.** Subgroup analysis of BV/TV based on different administration frequencies. Data are presented as standardized mean difference (SMD) with 95% confidence intervals (CI).

**
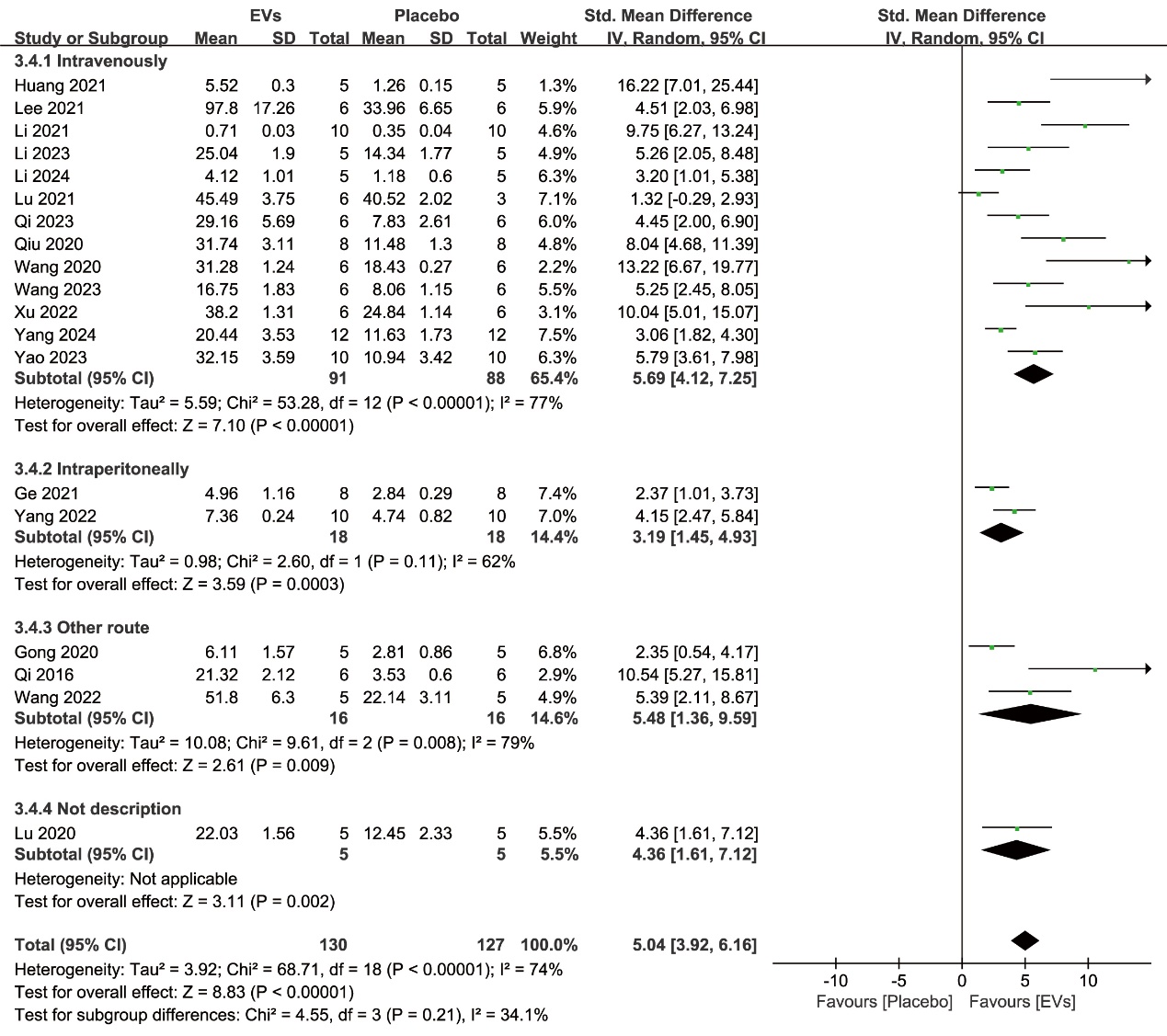
**

**Figure S15.** Subgroup analysis of BV/TV based on different administration routes. Data are presented as standardized mean difference (SMD) with 95% confidence intervals (CI).

**
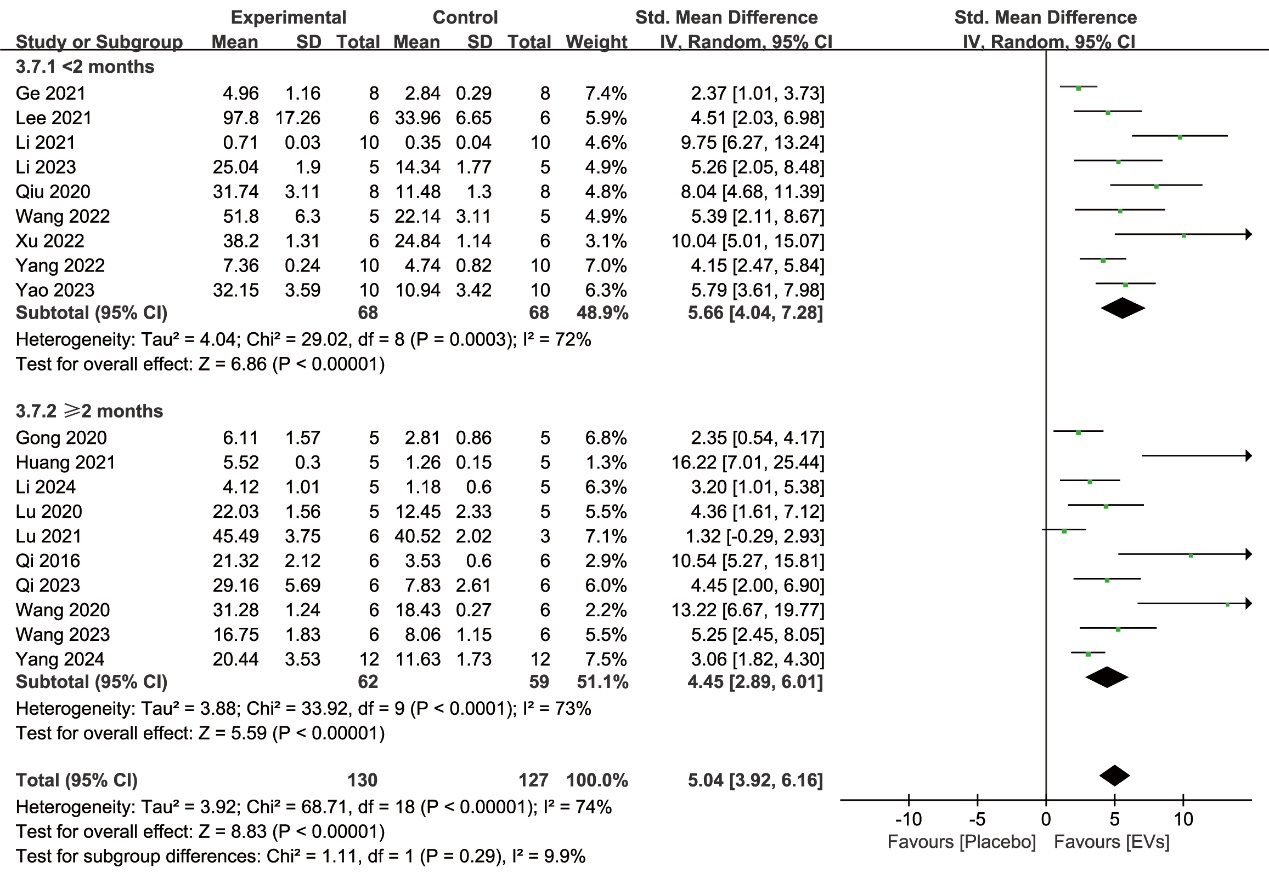
**

**Figure S16.** Subgroup analysis of BV/TV based on different treatment durations. Data are presented as standardized mean difference (SMD) with 95% confidence intervals (CI).


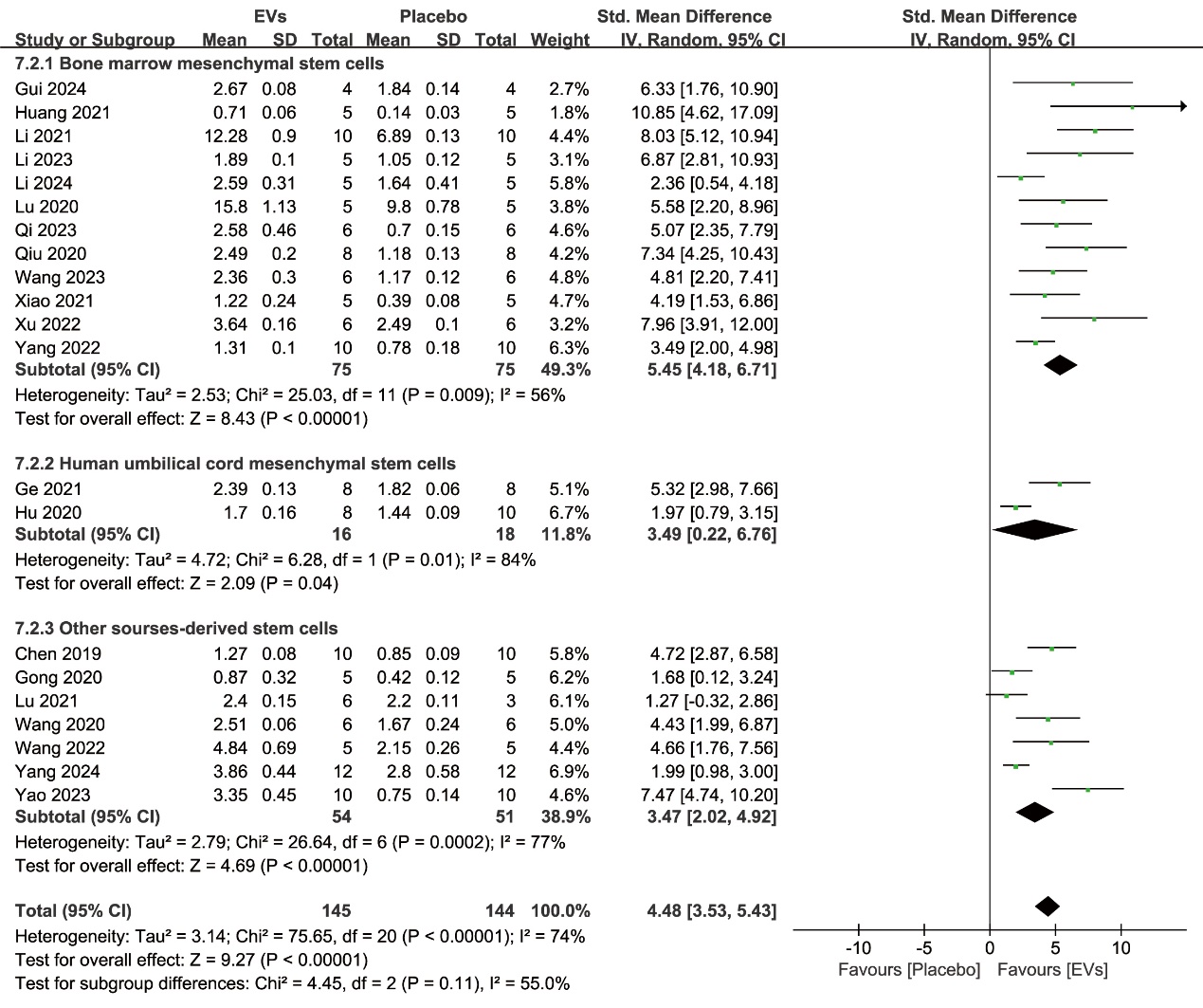


**Figure S17.** Subgroup analysis based on different SC-EVs sources for Tb. N. Data are presented as standardized mean difference (SMD) with 95% confidence intervals (CI).


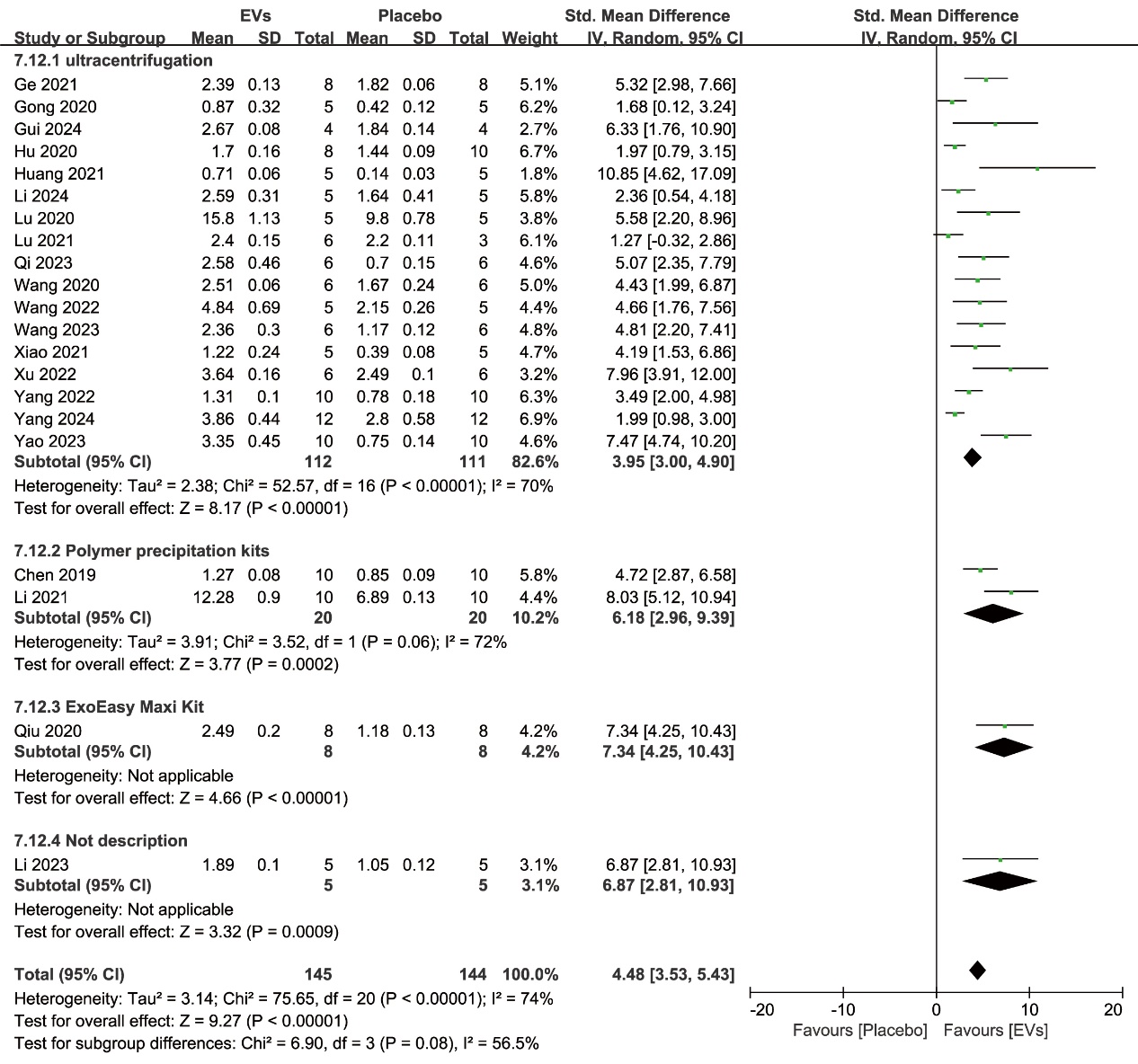


**Figure S18.** Subgroup analysis of Tb. N based on different SC-EVs isolation methods. Data are presented as standardized mean difference (SMD) with 95% confidence intervals (CI).

**
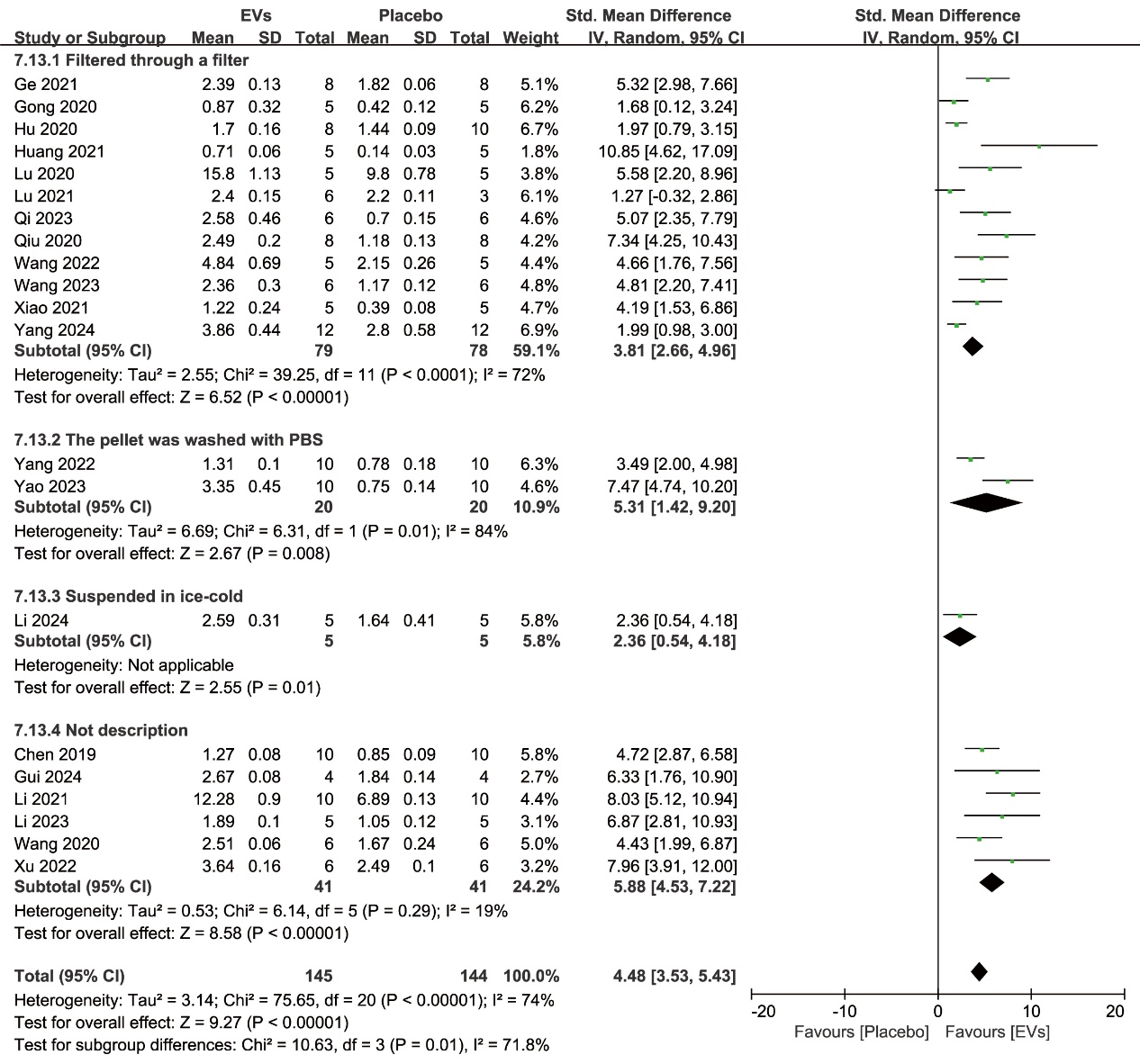
**

**Figure S19.** Subgroup analysis of Tb. N based on different SC-EVs purification methods. Data are presented as standardized mean difference (SMD) with 95% confidence intervals (CI).


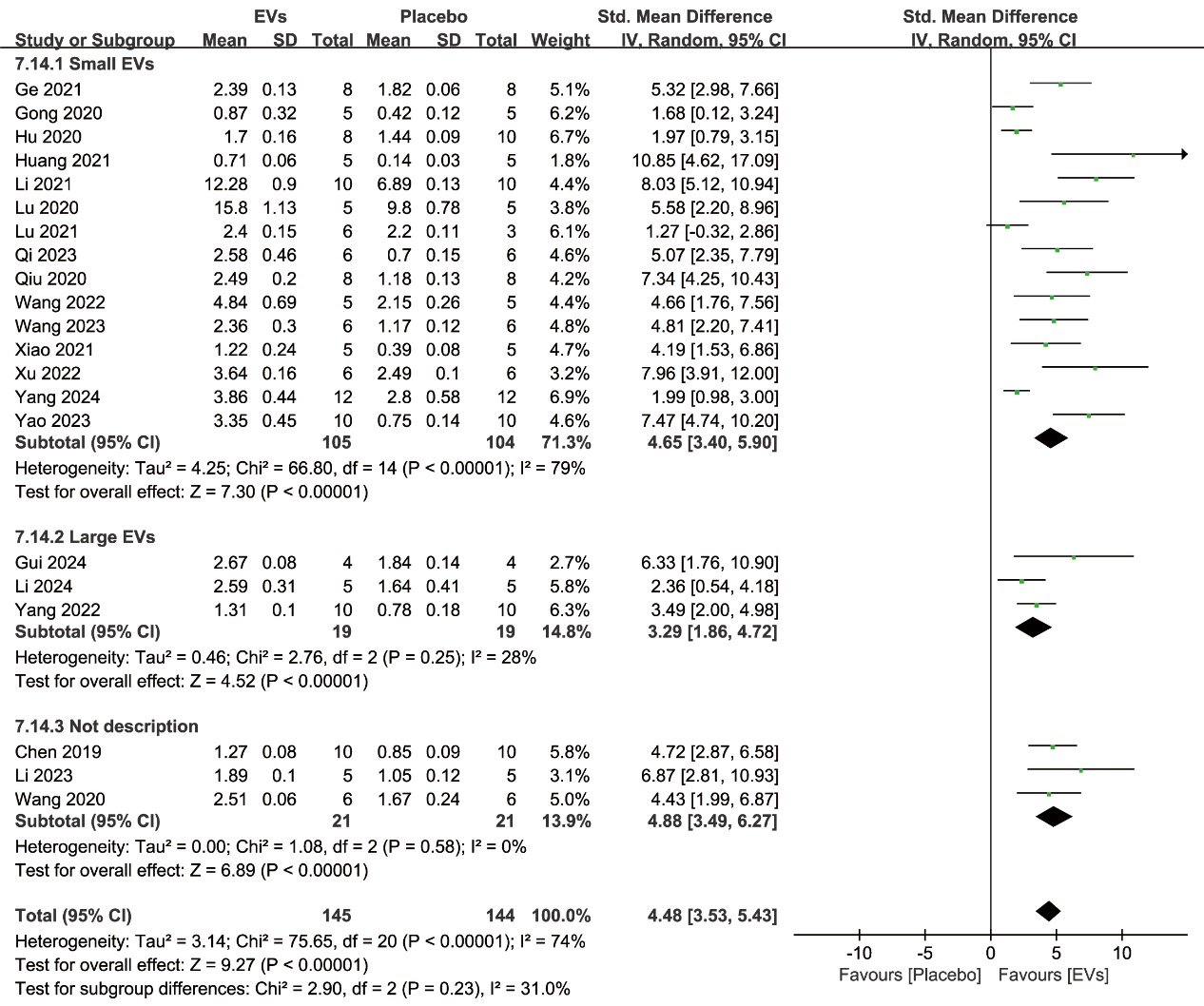


**Figure S20.** Subgroup analysis of Tb. N based on different SC-EV sizes. Data are presented as standardized mean difference (SMD) with 95% confidence intervals (CI).


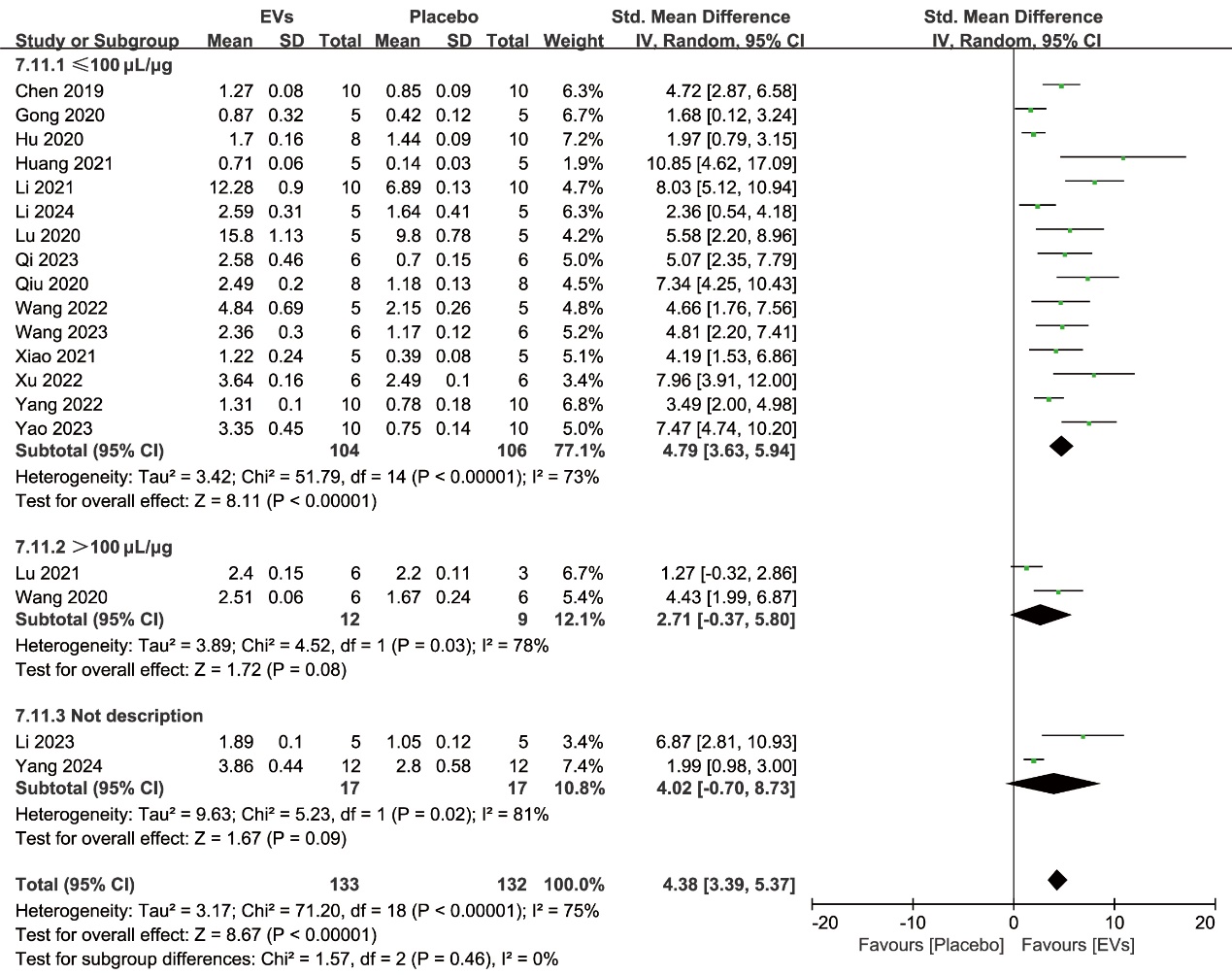


**Figure S21.** Subgroup analysis of Tb. N based on different SC-EV intervention doses. Data are presented as standardized mean difference (SMD) with 95% confidence intervals (CI).


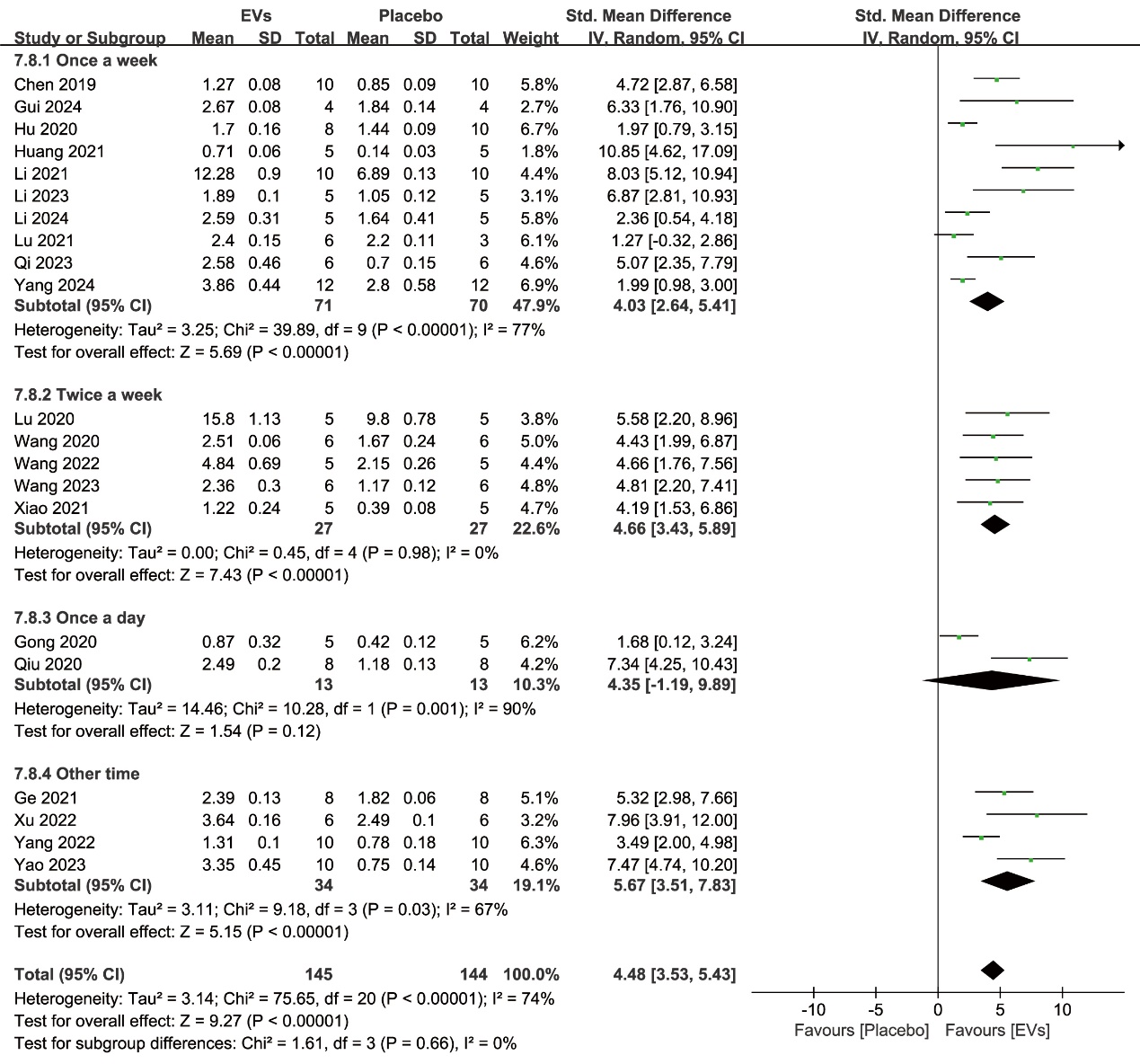


**Figure S22.** Subgroup analysis of Tb. N based on different administration frequencies. Data are presented as standardized mean difference (SMD) with 95% confidence intervals (CI).


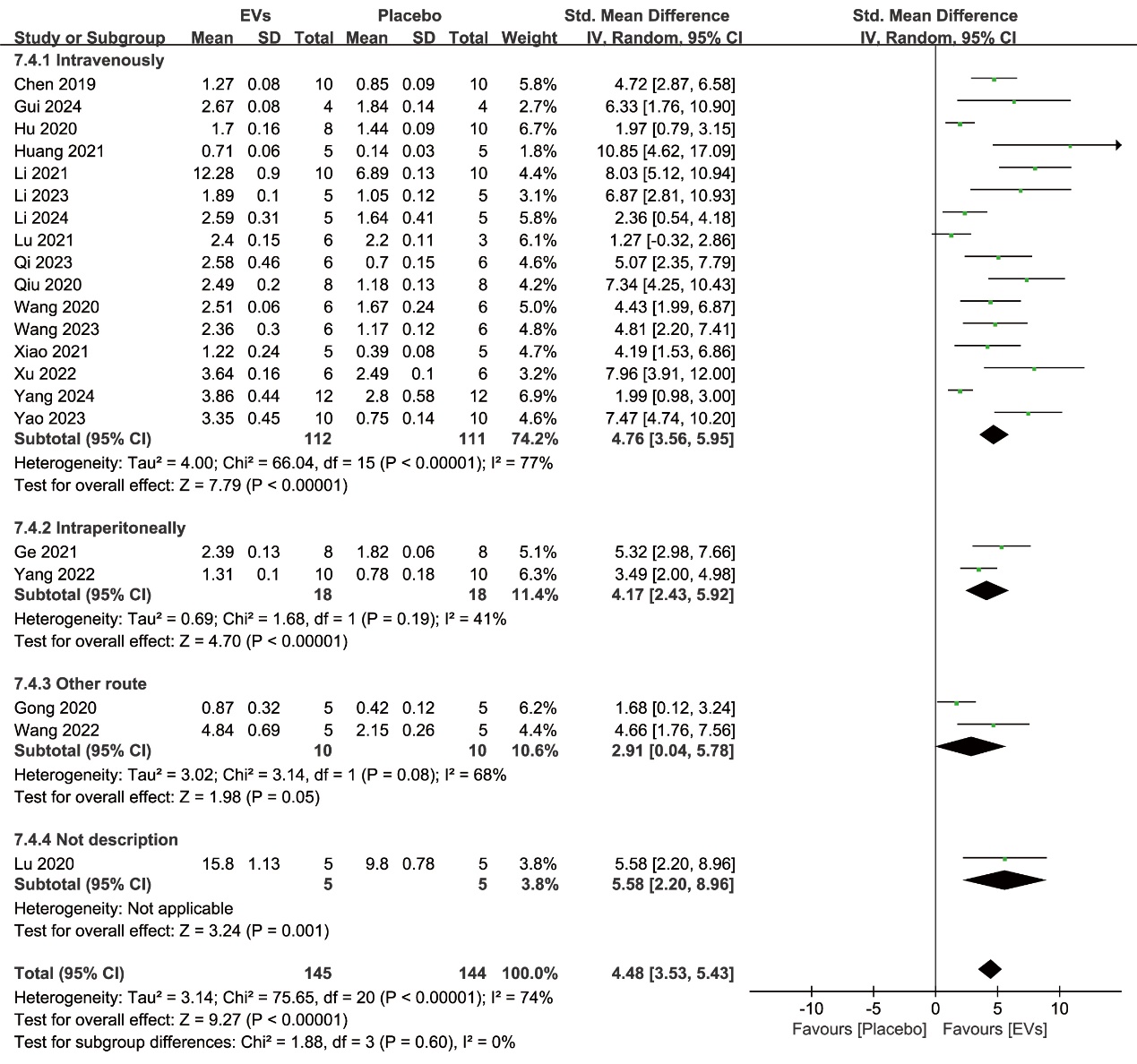


**Figure S23.** Subgroup analysis of Tb. N based on different administration routes. Data are presented as standardized mean difference (SMD) with 95% confidence intervals (CI).


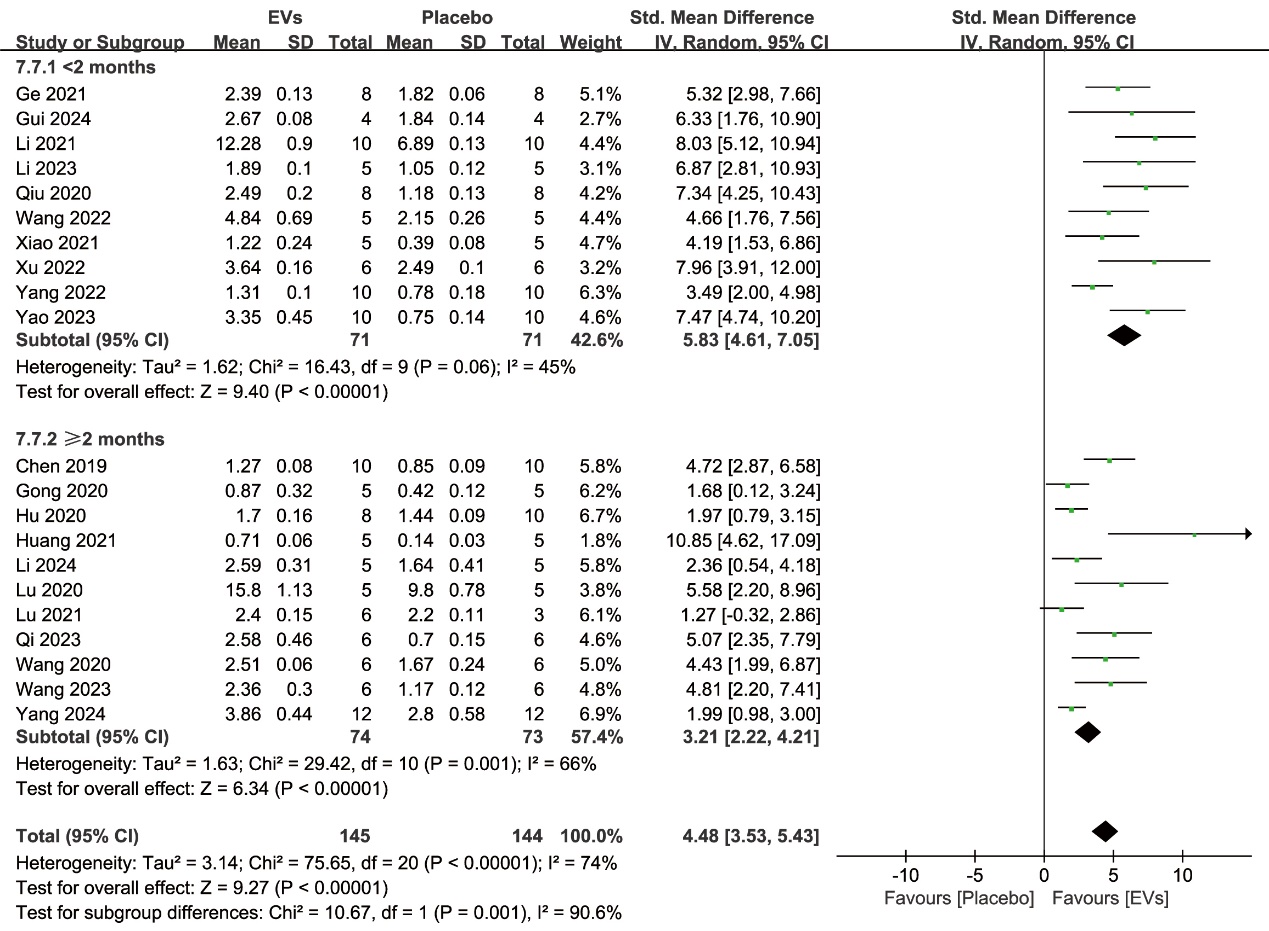


**Figure S24.** Subgroup analysis of Tb. N based on different treatment durations. Data are presented as standardized mean difference (SMD) with 95% confidence intervals (CI).


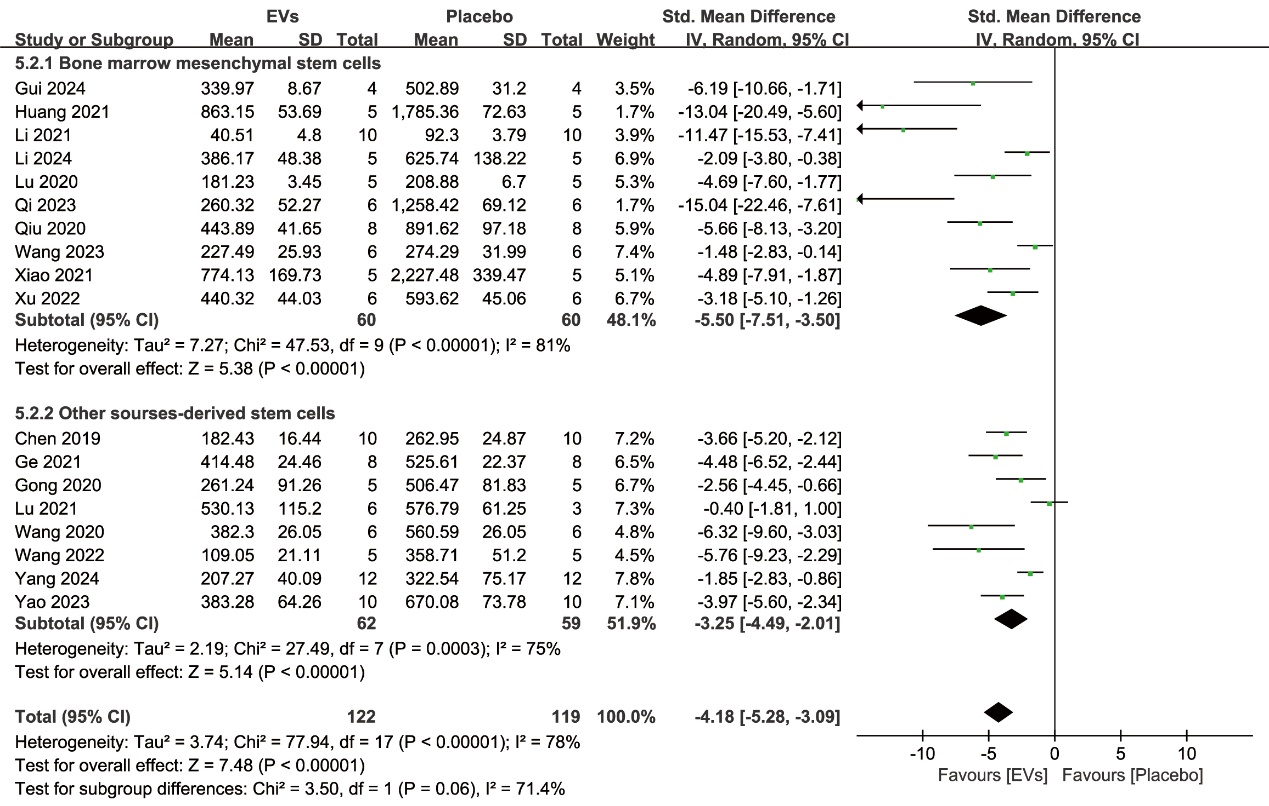


**Figure S25.** Subgroup analysis based on different SC-EVs sources for Tb. Sp. Data are presented as standardized mean difference (SMD) with 95% confidence intervals (CI).


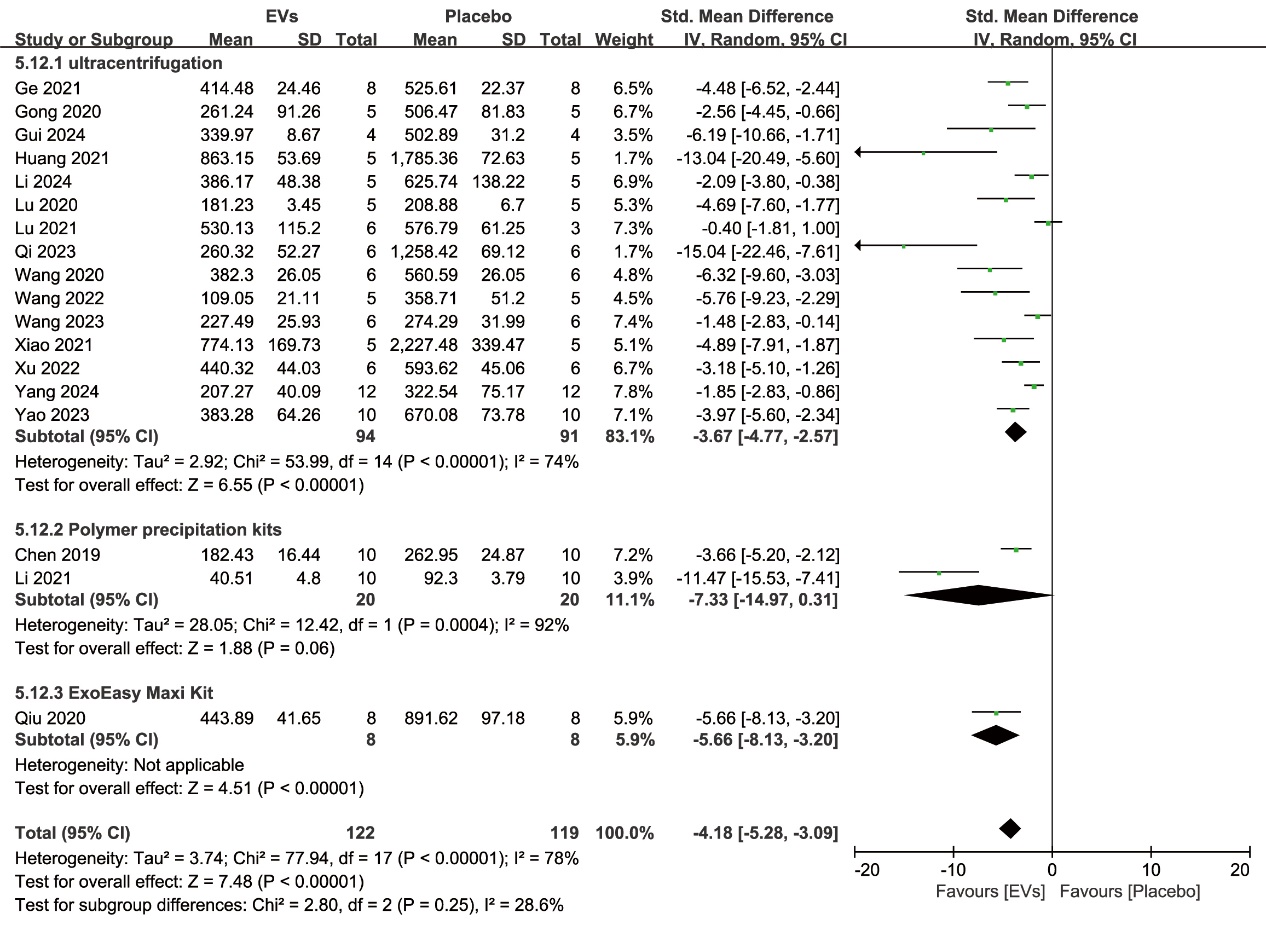


**Figure S26.** Subgroup analysis of Tb. Sp based on different SC-EVs isolation methods. Data are presented as standardized mean difference (SMD) with 95% confidence intervals (CI).


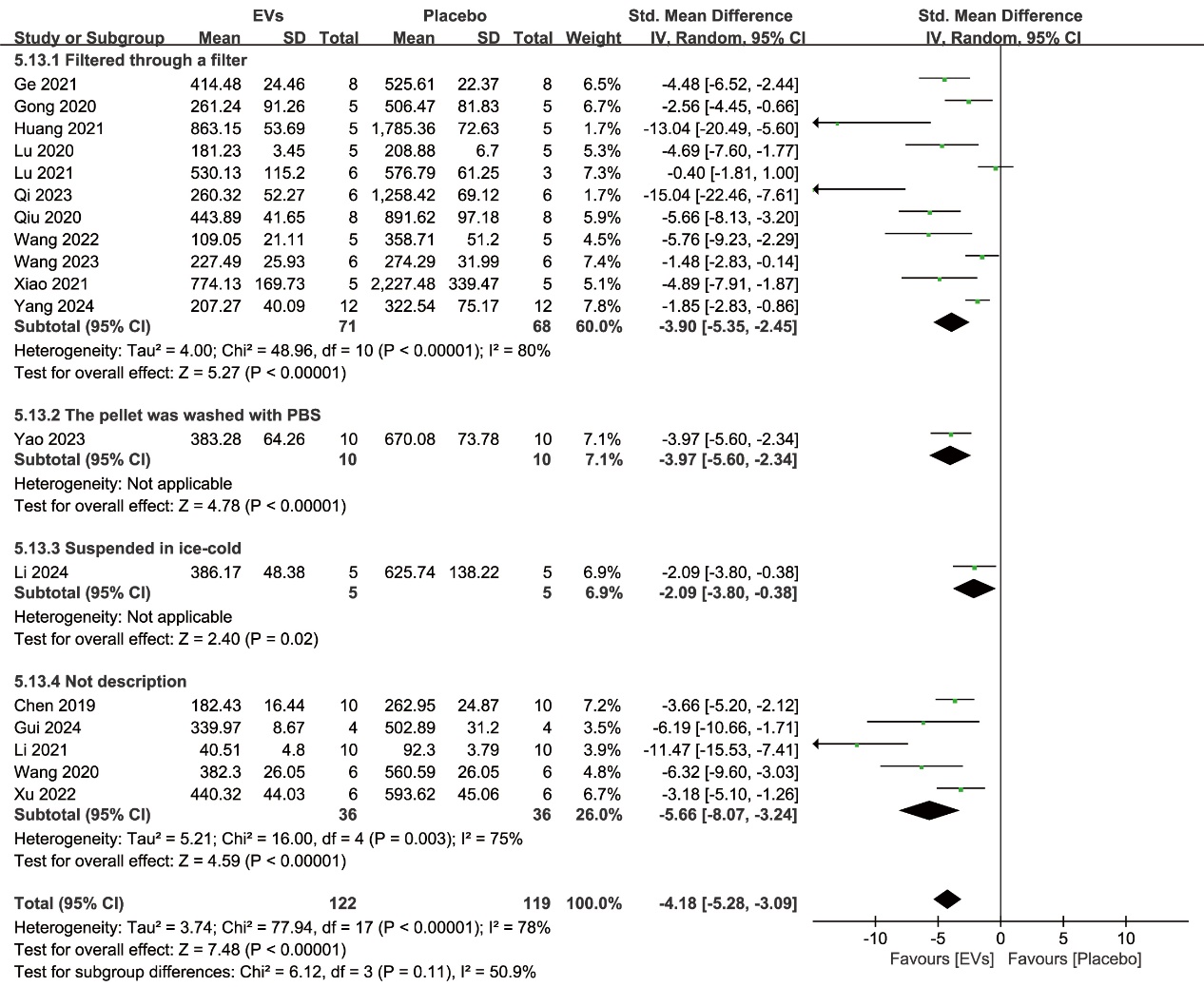


**Figure S27.** Subgroup analysis of Tb. Sp based on different SC-EVs purification methods. Data are presented as standardized mean difference (SMD) with 95% confidence intervals (CI).


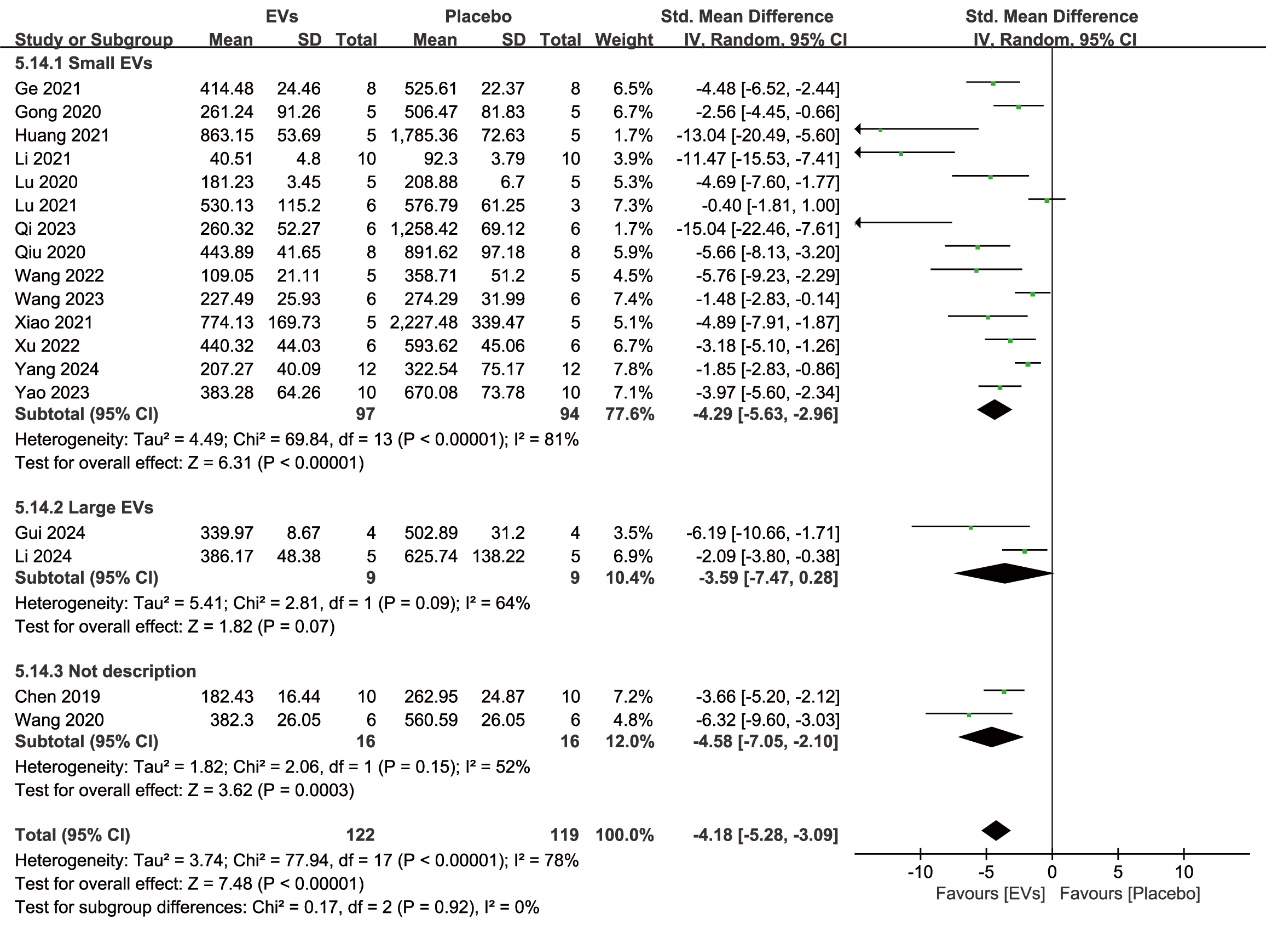


**Figure S28** Subgroup analysis of Tb. Sp based on different SC-EV sizes. Data are presented as standardized mean difference (SMD) with 95% confidence intervals (CI).


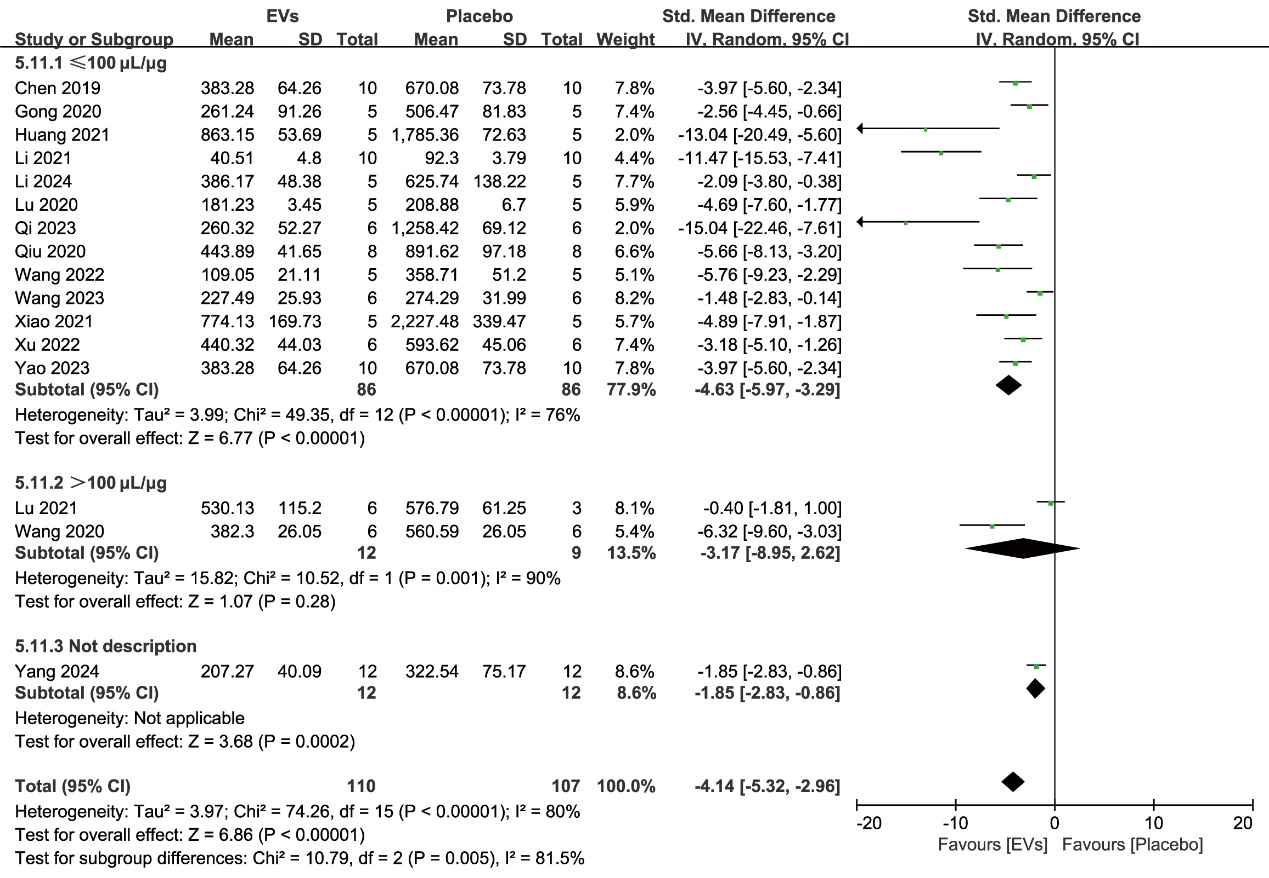


**Figure S29.** Subgroup analysis of Tb. Sp based on different SC-EV intervention doses. Data are presented as standardized mean difference (SMD) with 95% confidence intervals (CI).


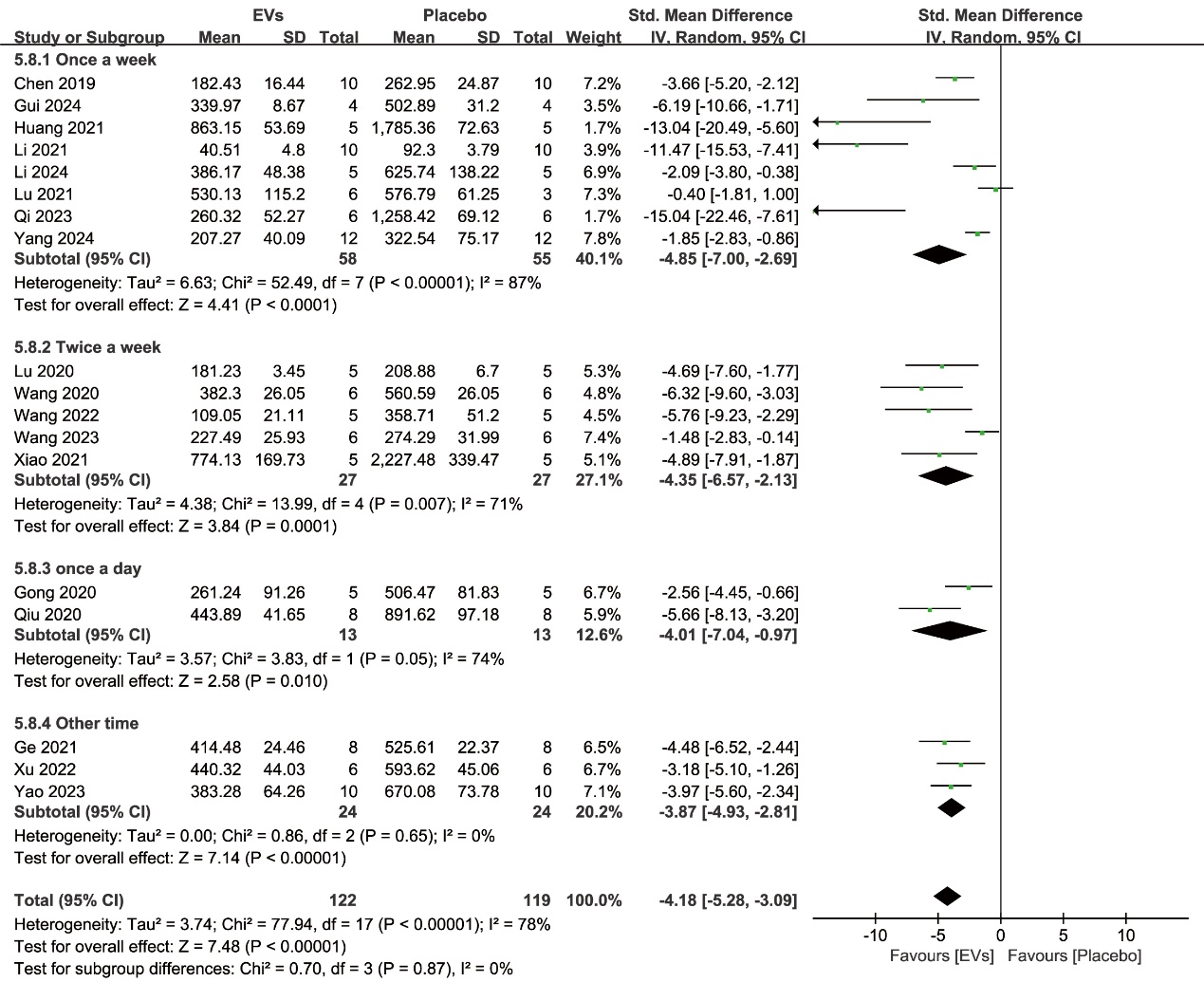


**Figure S30.** Subgroup analysis of Tb. Sp based on different administration frequencies. Data are presented as standardized mean difference (SMD) with 95% confidence intervals (CI).


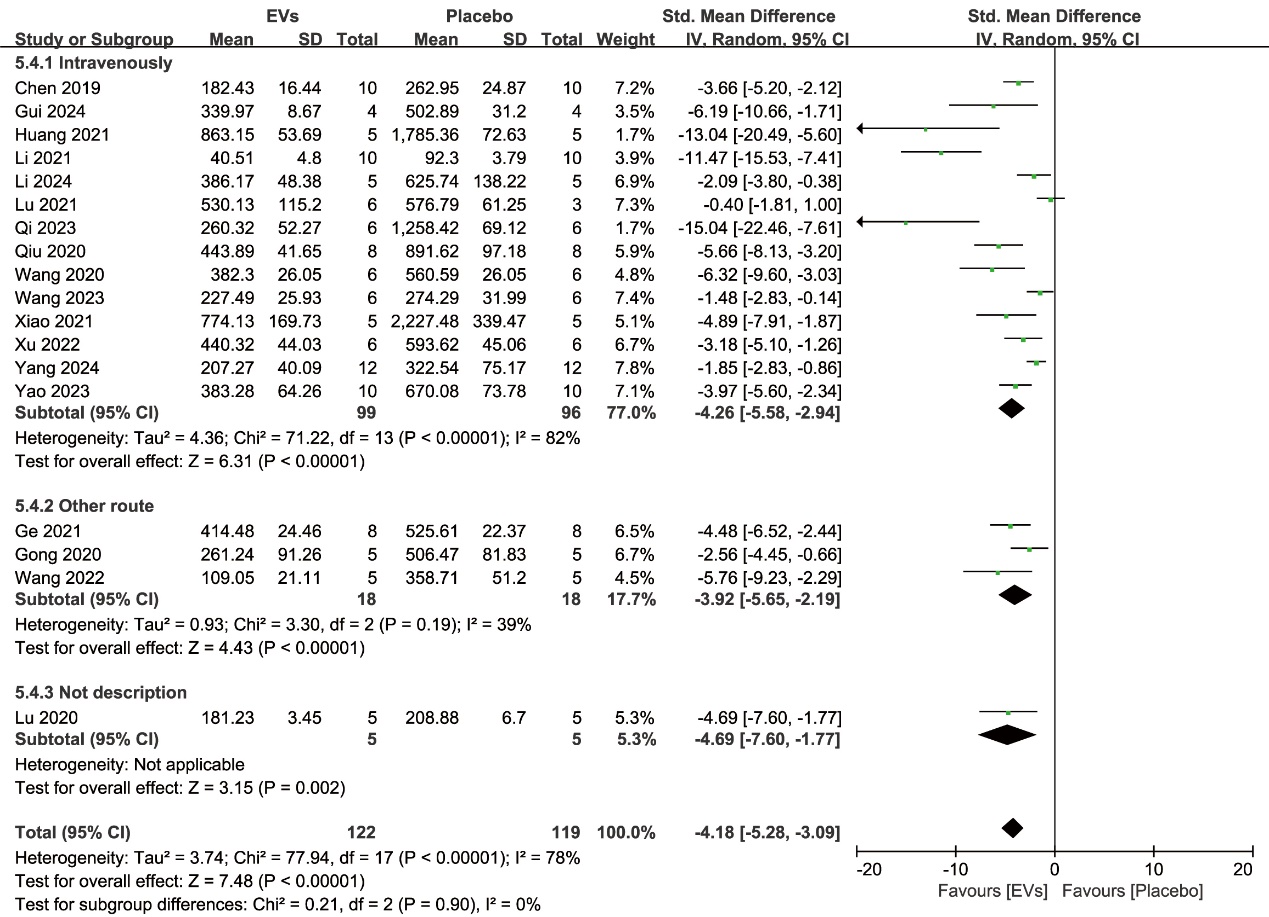


**Figure S31.** Subgroup analysis of Tb. Sp based on different administration routes. Data are presented as standardized mean difference (SMD) with 95% confidence intervals (CI).


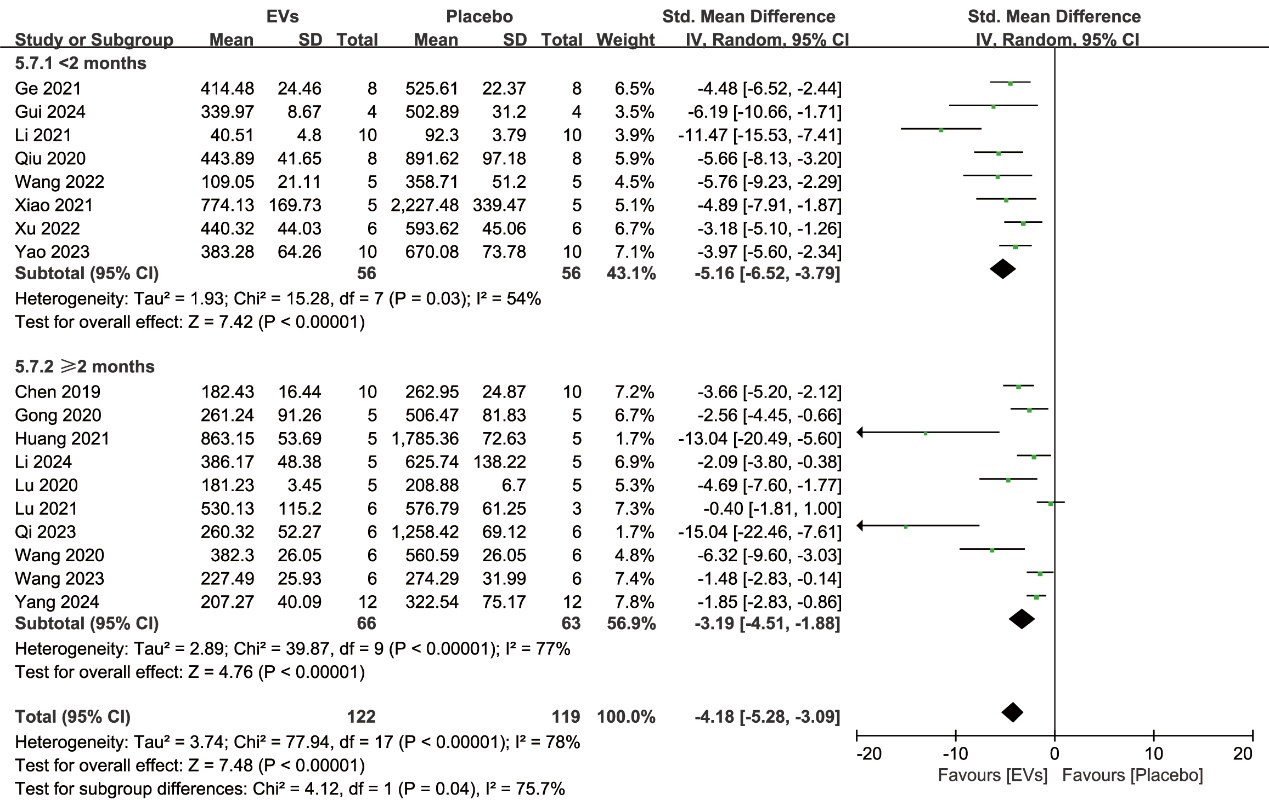


**Figure S32.** Subgroup analysis of Tb. Sp based on different treatment durations. Data are presented as standardized mean difference (SMD) with 95% confidence intervals (CI).


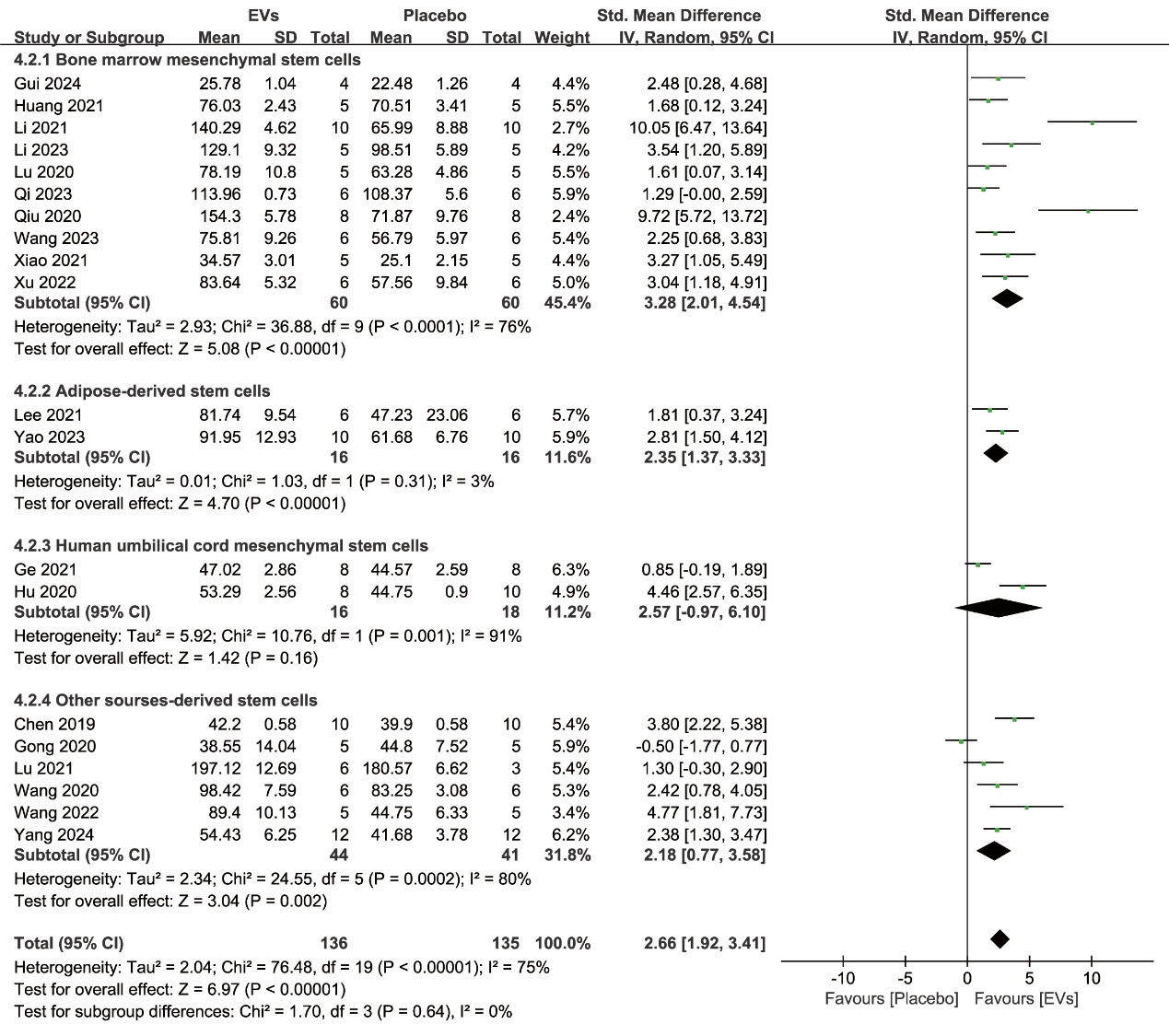


**Figure S33.** Subgroup analysis based on different SC-EVs sources for Tb. Th. Data are presented as standardized mean difference (SMD) with 95% confidence intervals (CI).


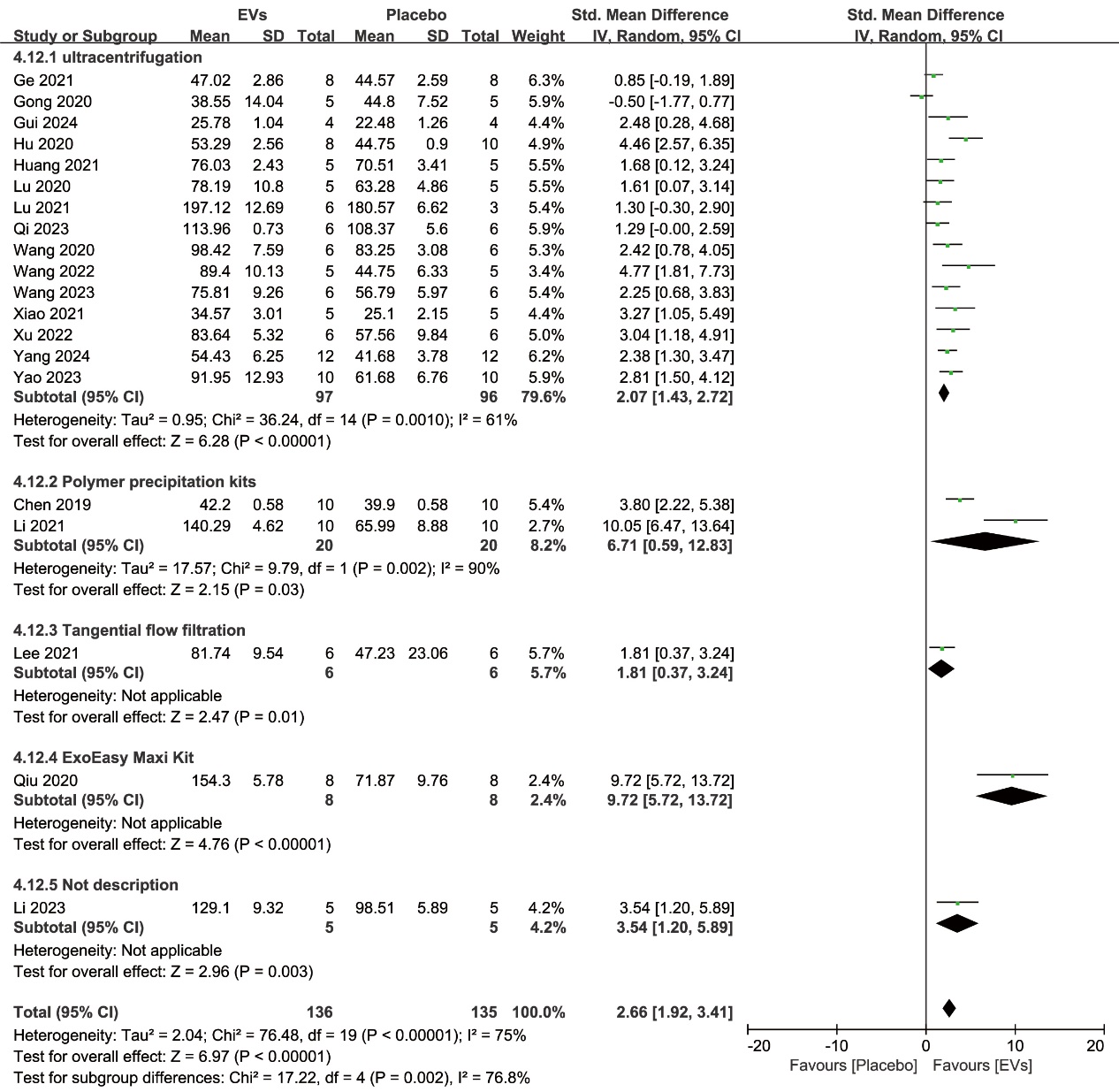


**Figure S34.** Subgroup analysis of Tb. Th based on different SC-EVs isolation methods. Data are presented as standardized mean difference (SMD) with 95% confidence intervals (CI).

**
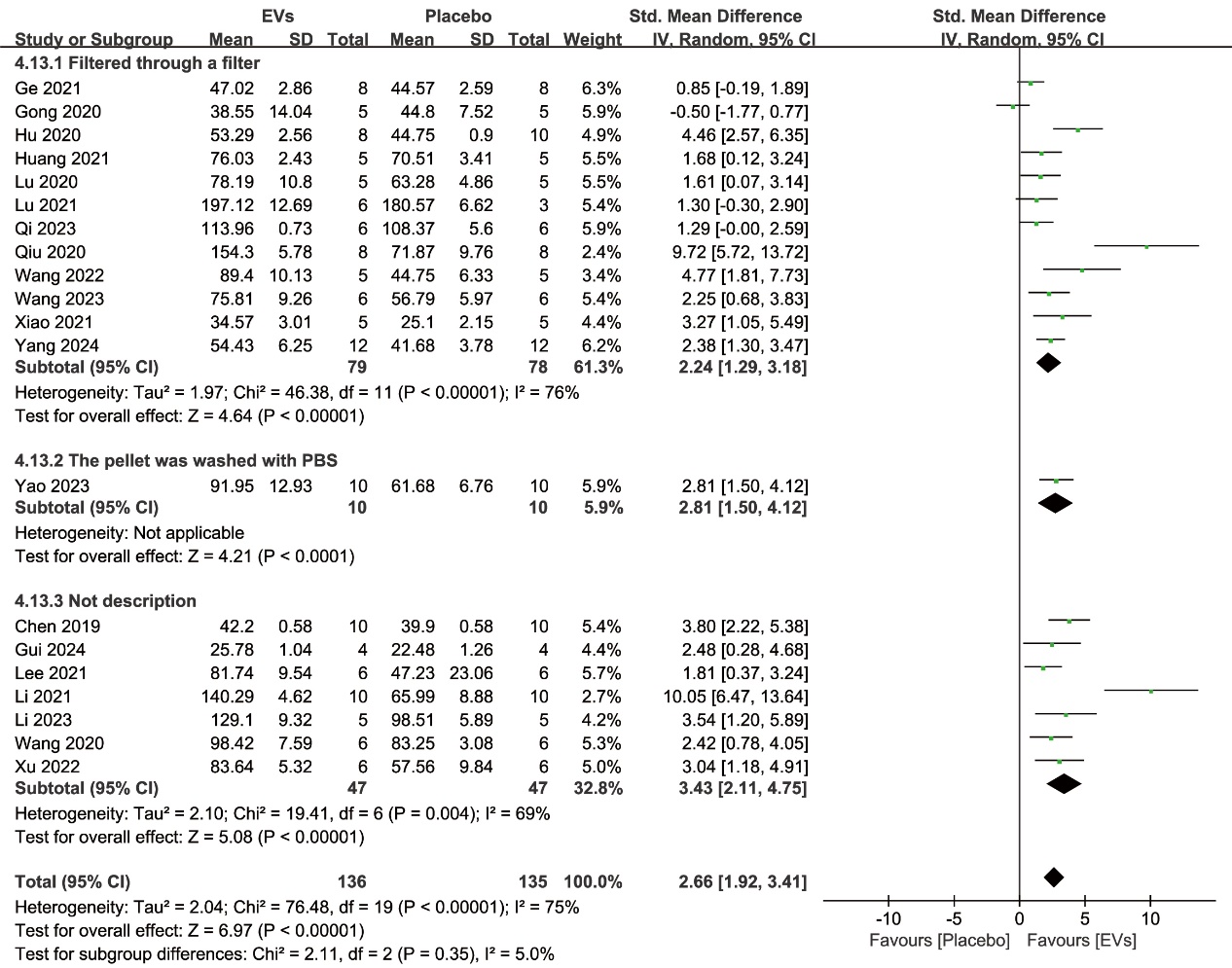
**

**Figure S35.** Subgroup analysis of Tb. Th based on different SC-EVs purification methods. Data are presented as standardized mean difference (SMD) with 95% confidence intervals (CI).

**
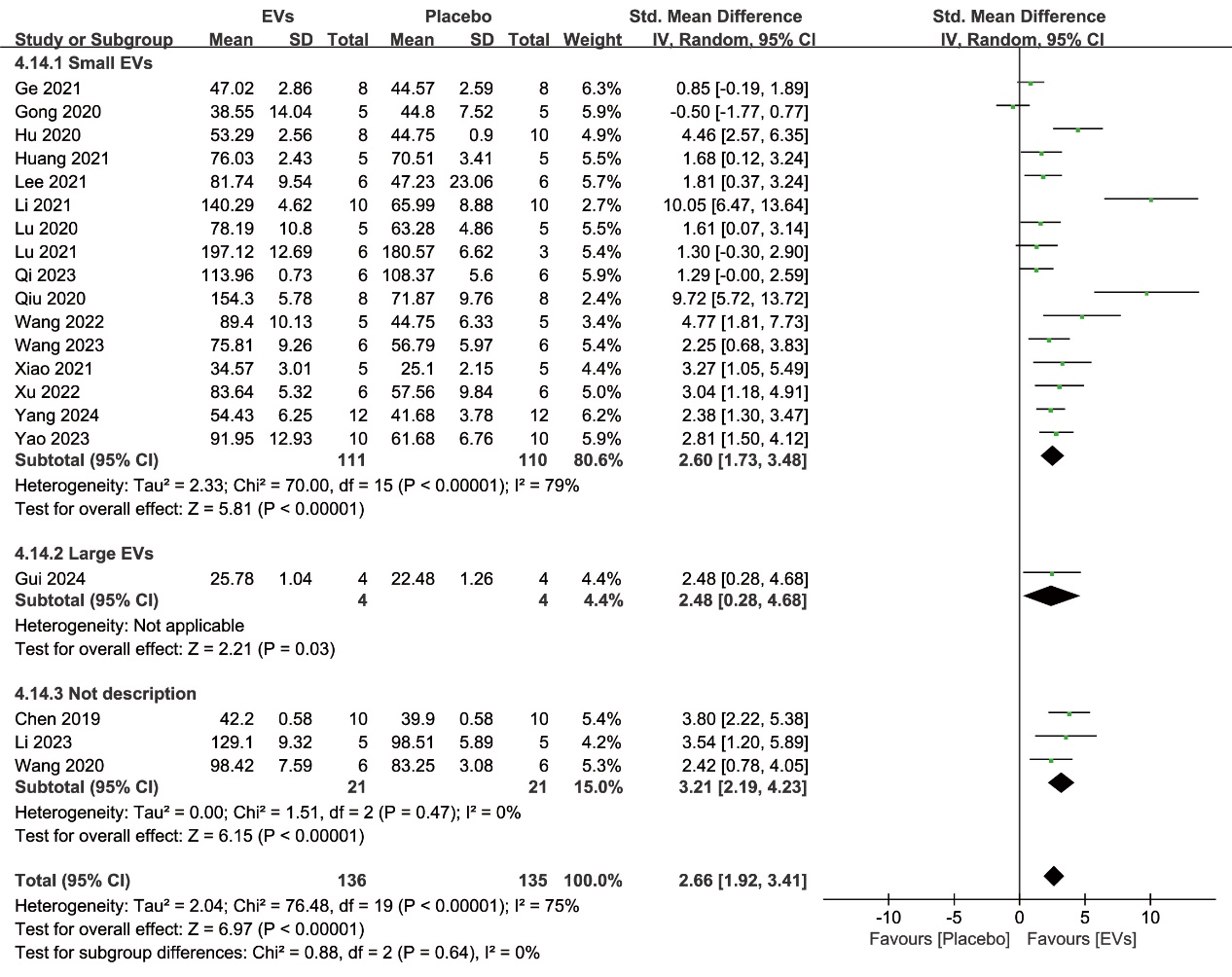
**

**Figure S36.** Subgroup analysis of Tb. Th based on different SC-EV sizes. Data are presented as standardized mean difference (SMD) with 95% confidence intervals (CI).


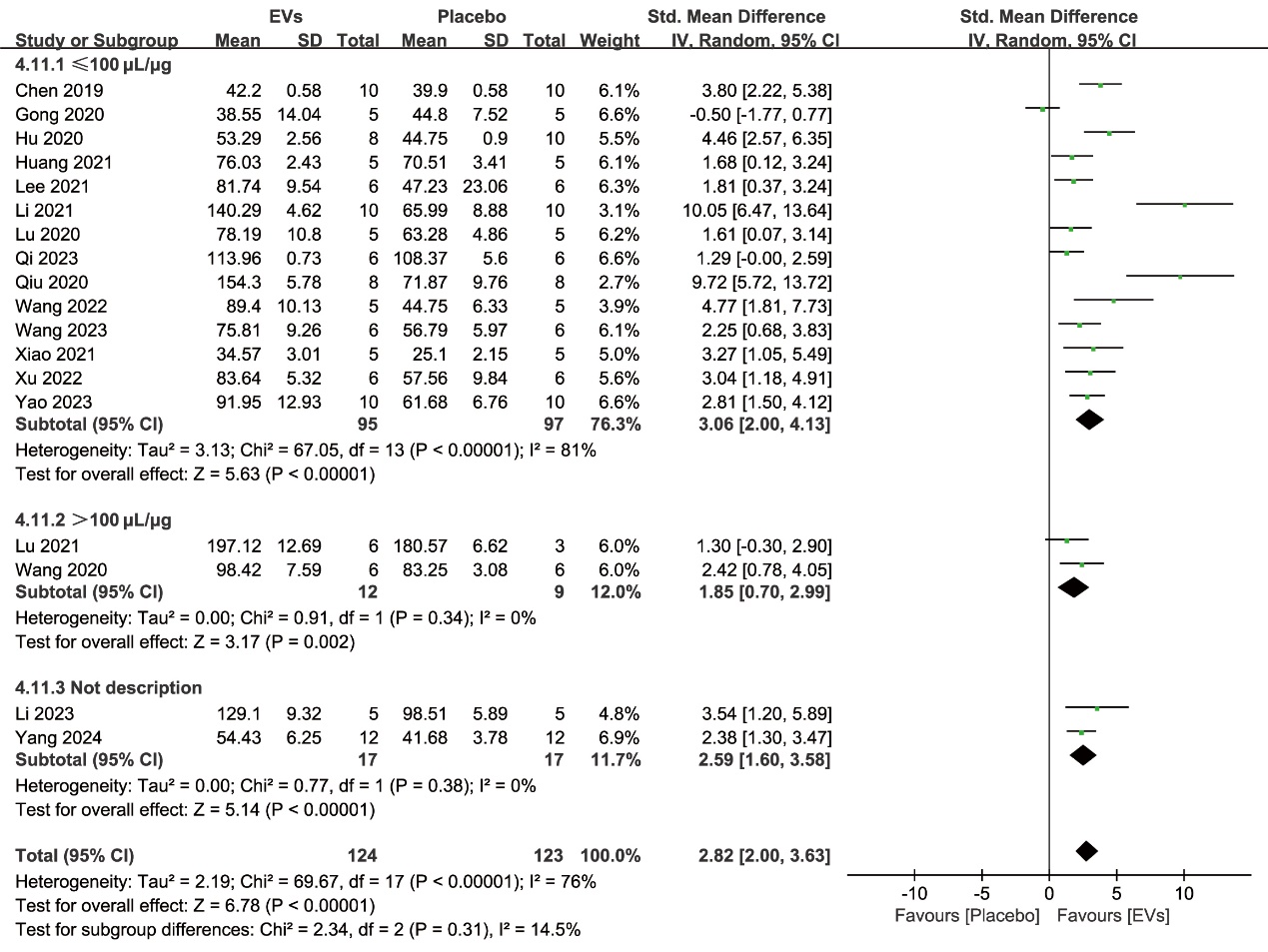


**Figure S37.** Subgroup analysis of Tb. Th based on different SC-EV intervention doses. Data are presented as standardized mean difference (SMD) with 95% confidence intervals (CI).


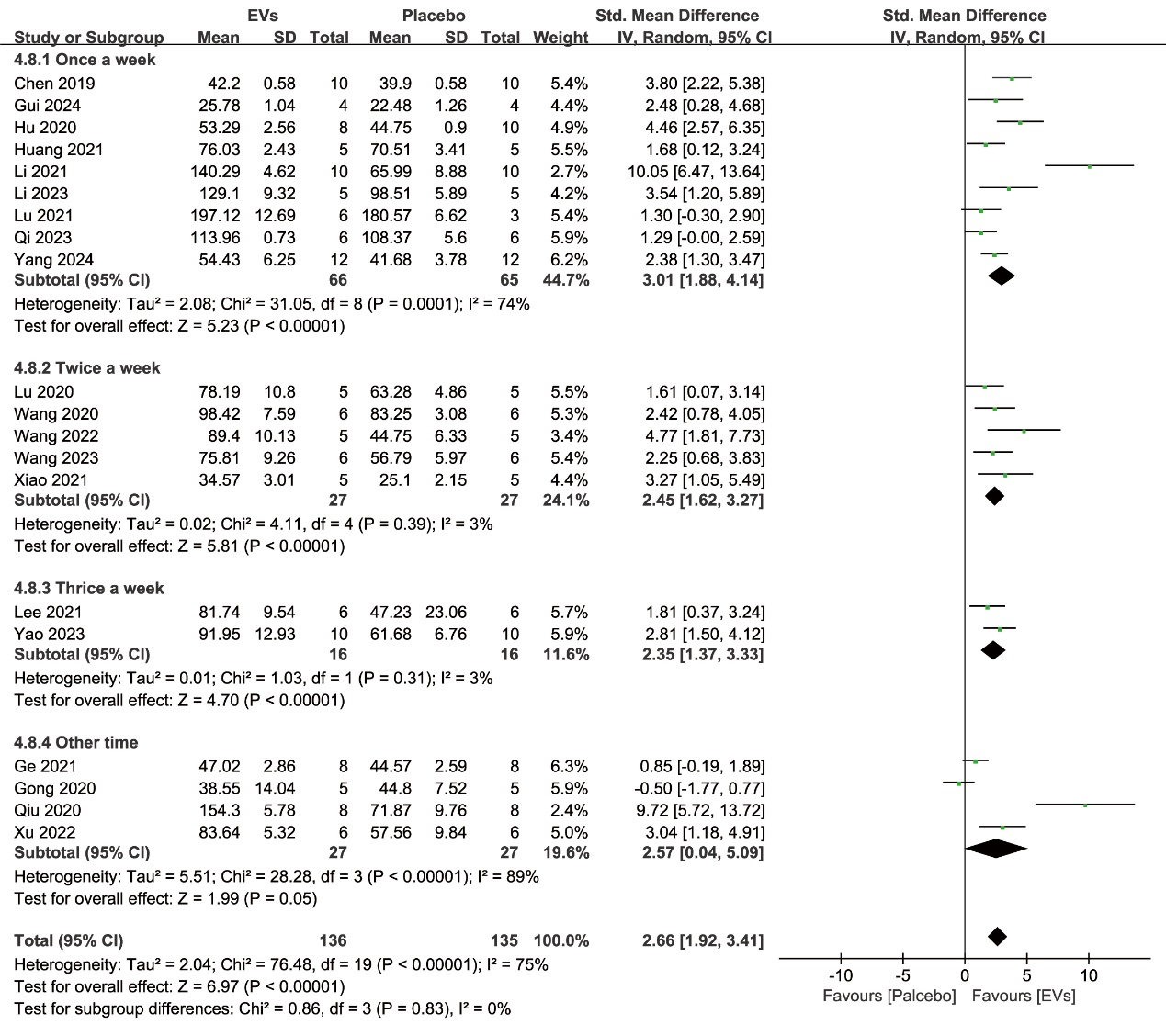


**Figure S38.** Subgroup analysis of Tb. Th based on different administration frequencies. Data are presented as standardized mean difference (SMD) with 95% confidence intervals (CI).


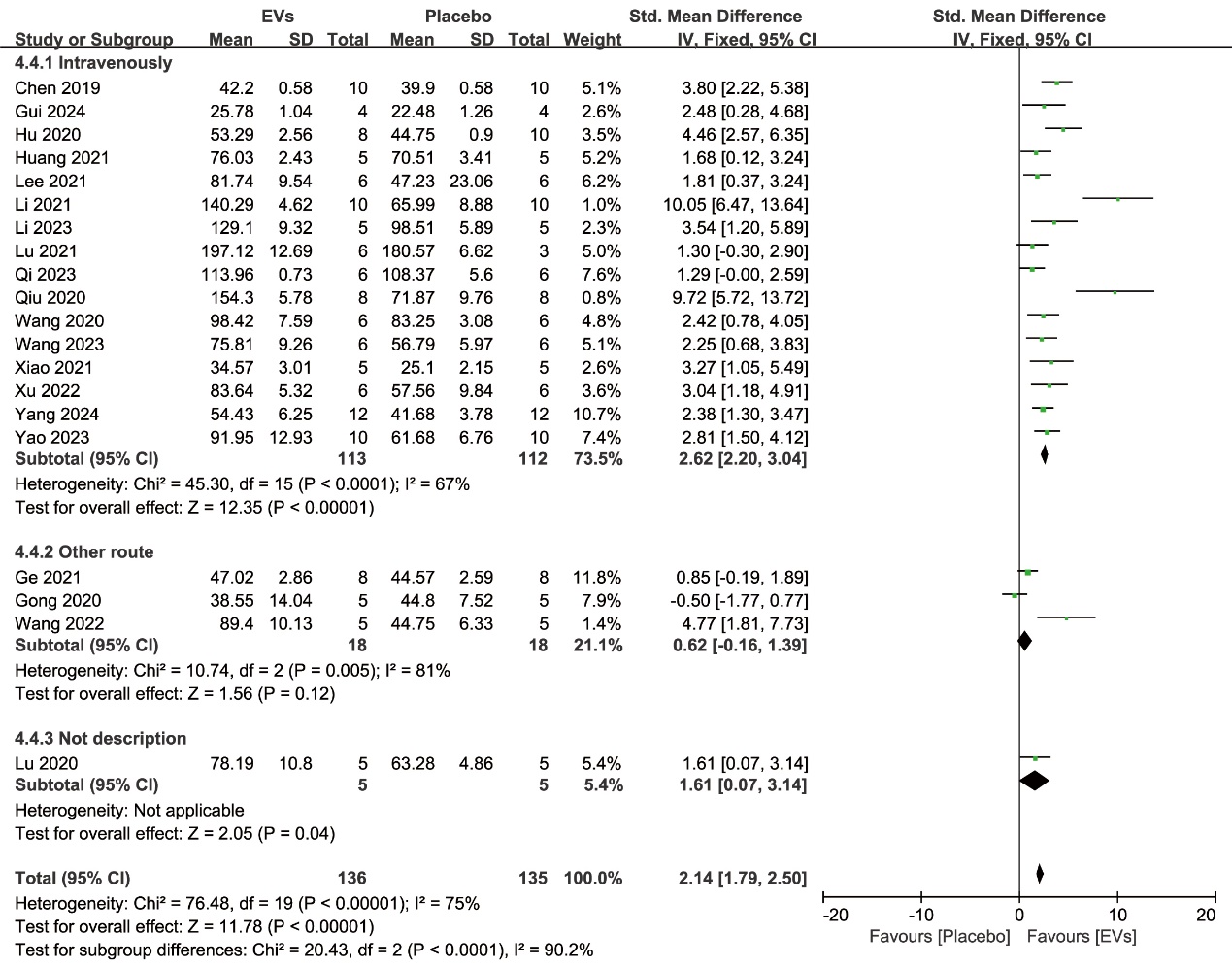


**Figure S39.** Subgroup analysis of Tb. Th based on different administration routes. Data are presented as standardized mean difference (SMD) with 95% confidence intervals (CI).


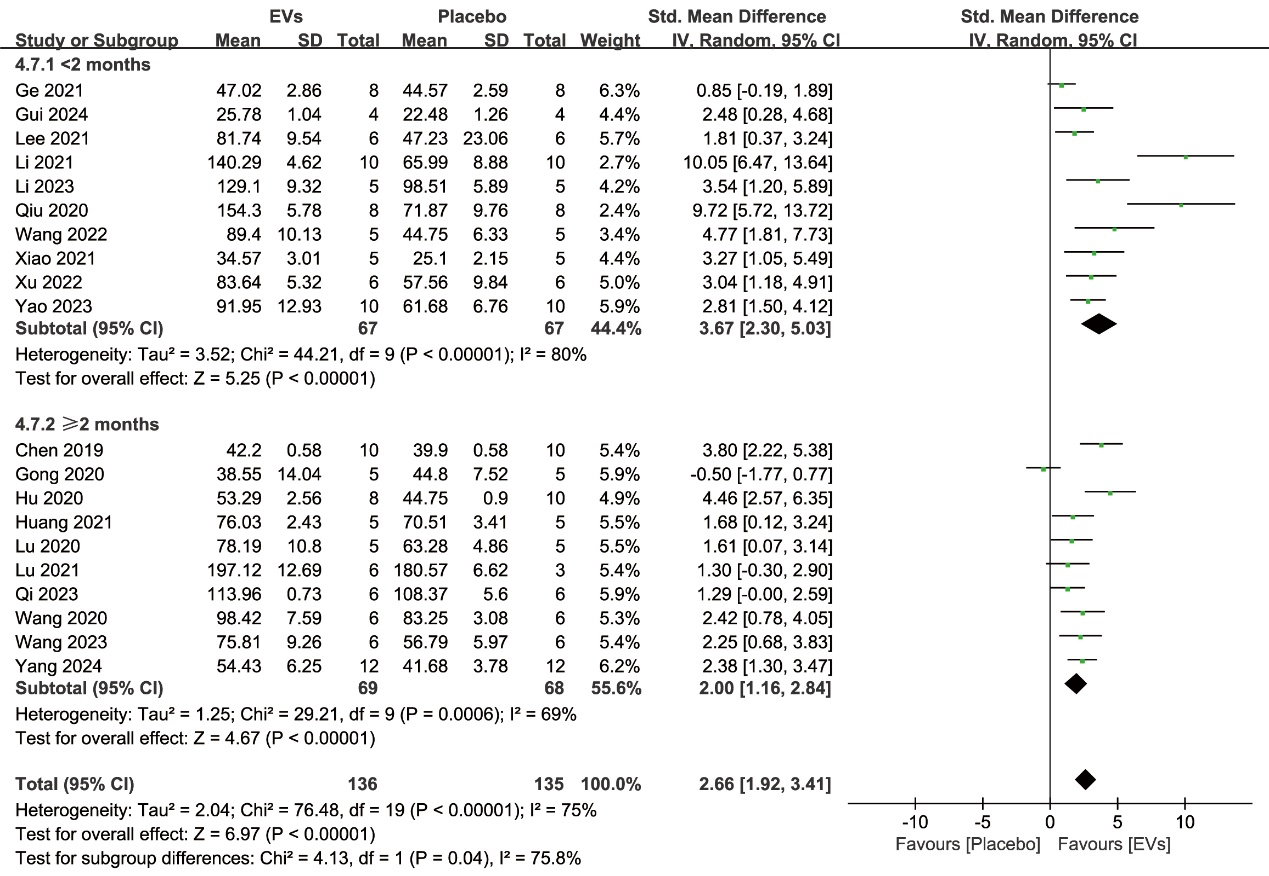


**Figure S40.** Subgroup analysis of Tb. Th based on different treatment durations. Data are presented as standardized mean difference (SMD) with 95% confidence intervals (CI).

**
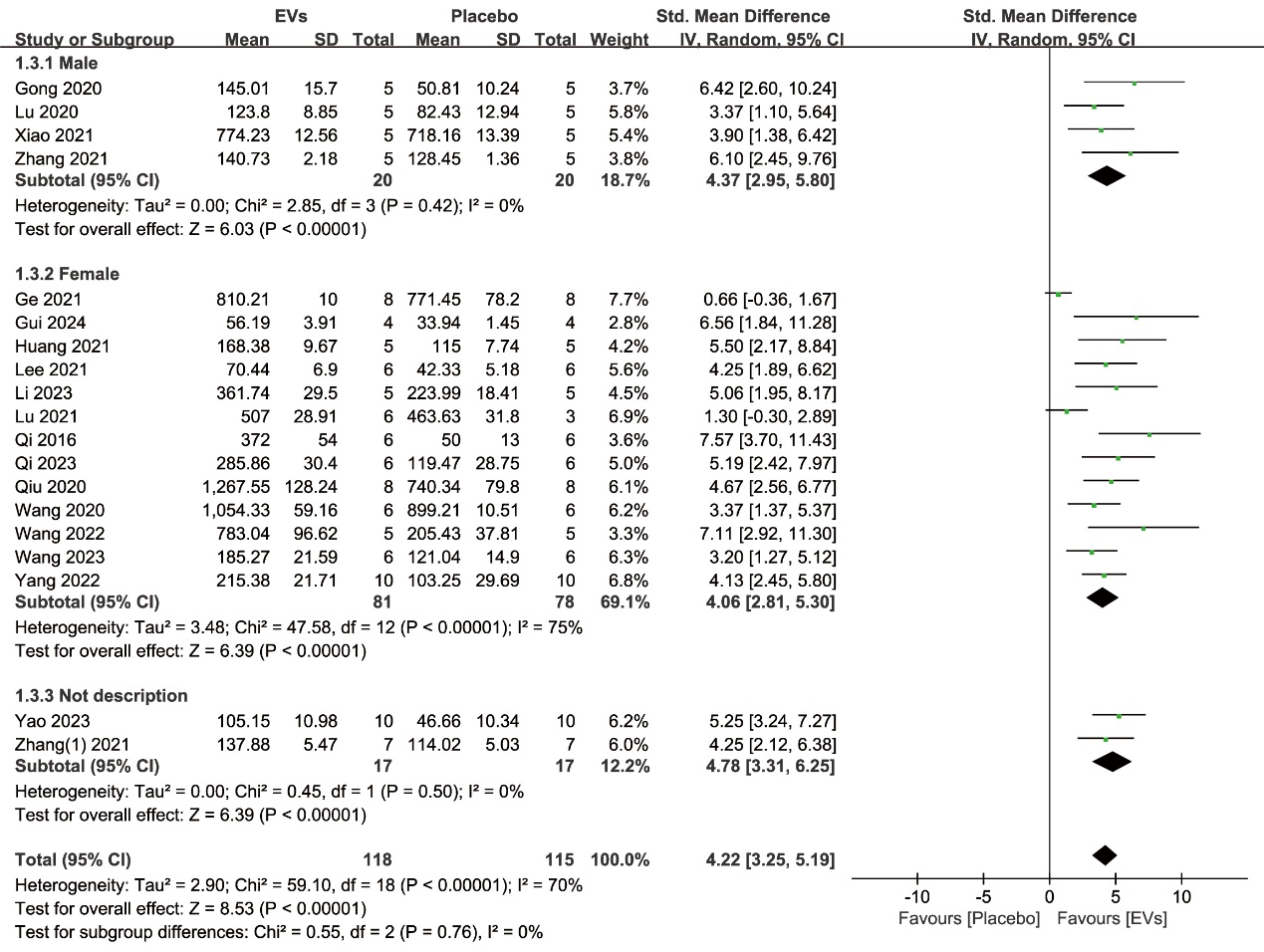
**

**Figure S41.** Subgroup analysis of bone mineral density (BMD) based on different animal sexes. Data are presented as standardized mean difference (SMD) with 95% confidence intervals (CI).


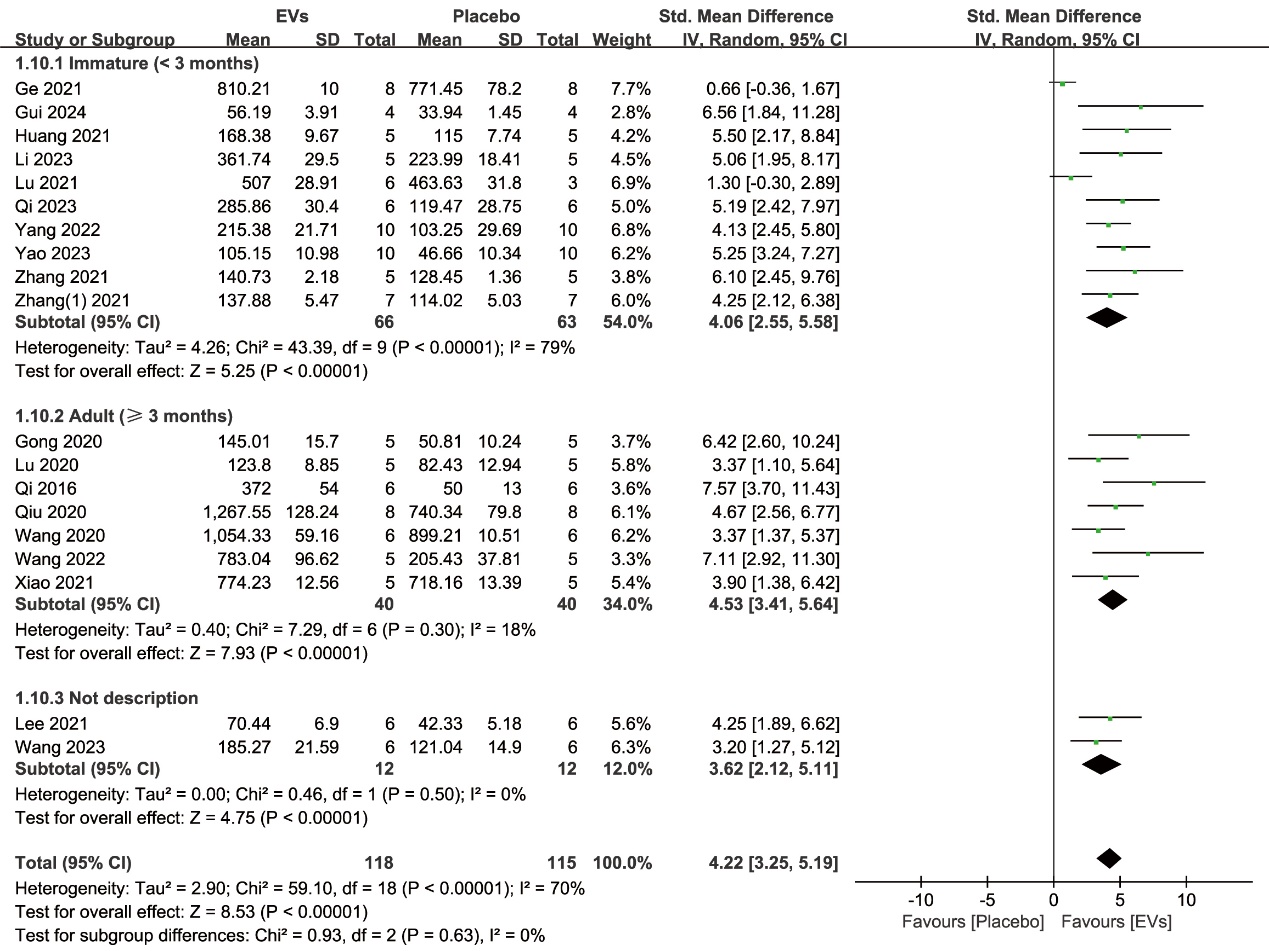


**Figure S42.** Subgroup analysis of bone mineral density (BMD) based on different animal ages. Data are presented as standardized mean difference (SMD) with 95% confidence intervals (CI).


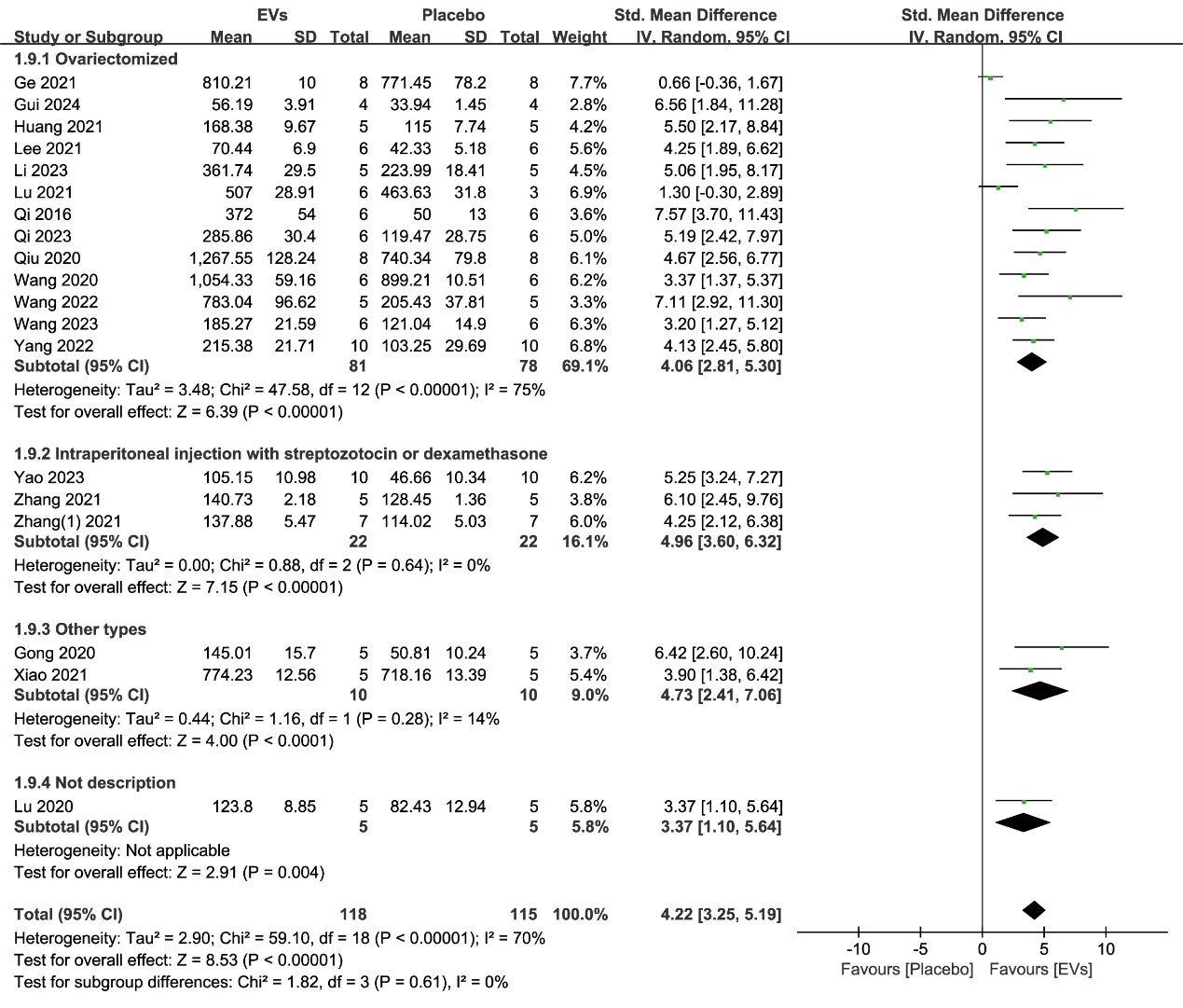


**Figure S43.** Subgroup analysis of bone mineral density (BMD) based on different modeling method. Data are presented as standardized mean difference (SMD) with 95% confidence intervals (CI).

**
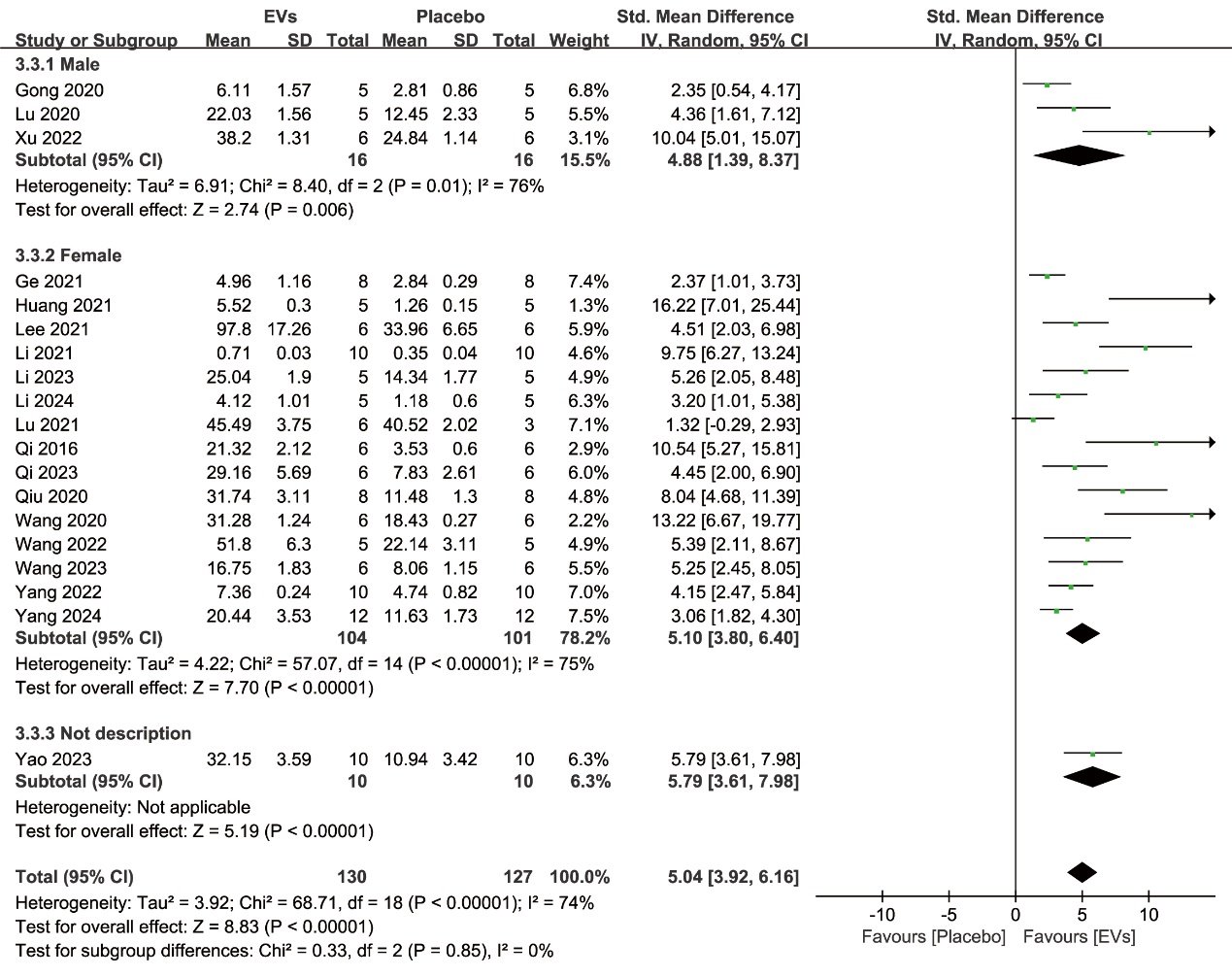
**

**Figure S44.** Subgroup analysis of BV/TV based on different animal sexes. Data are presented as standardized mean difference (SMD) with 95% confidence intervals (CI).

**
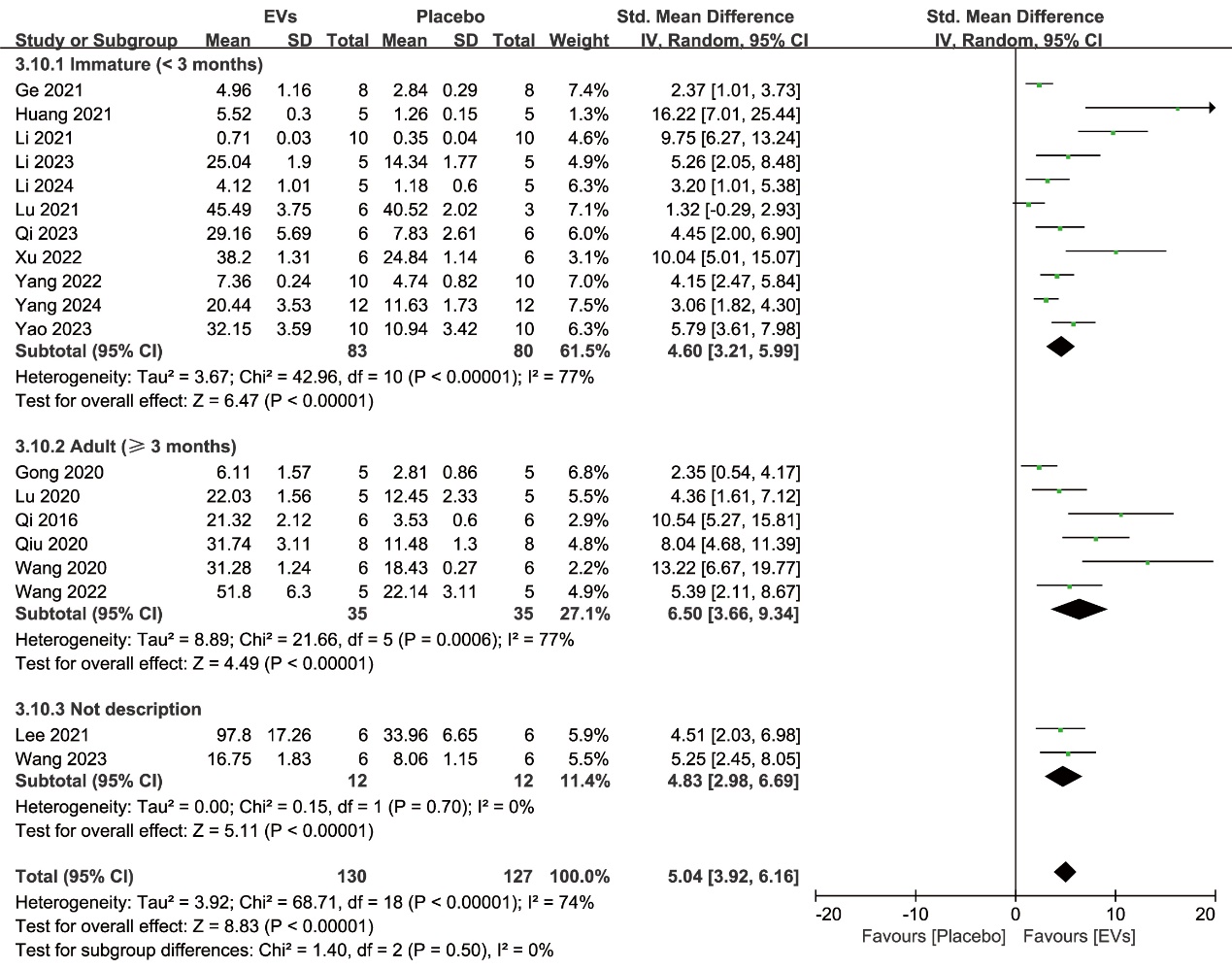
**

**Figure S45.** Subgroup analysis of BV/TV based on different animal ages. Data are presented as standardized mean difference (SMD) with 95% confidence intervals (CI).

**
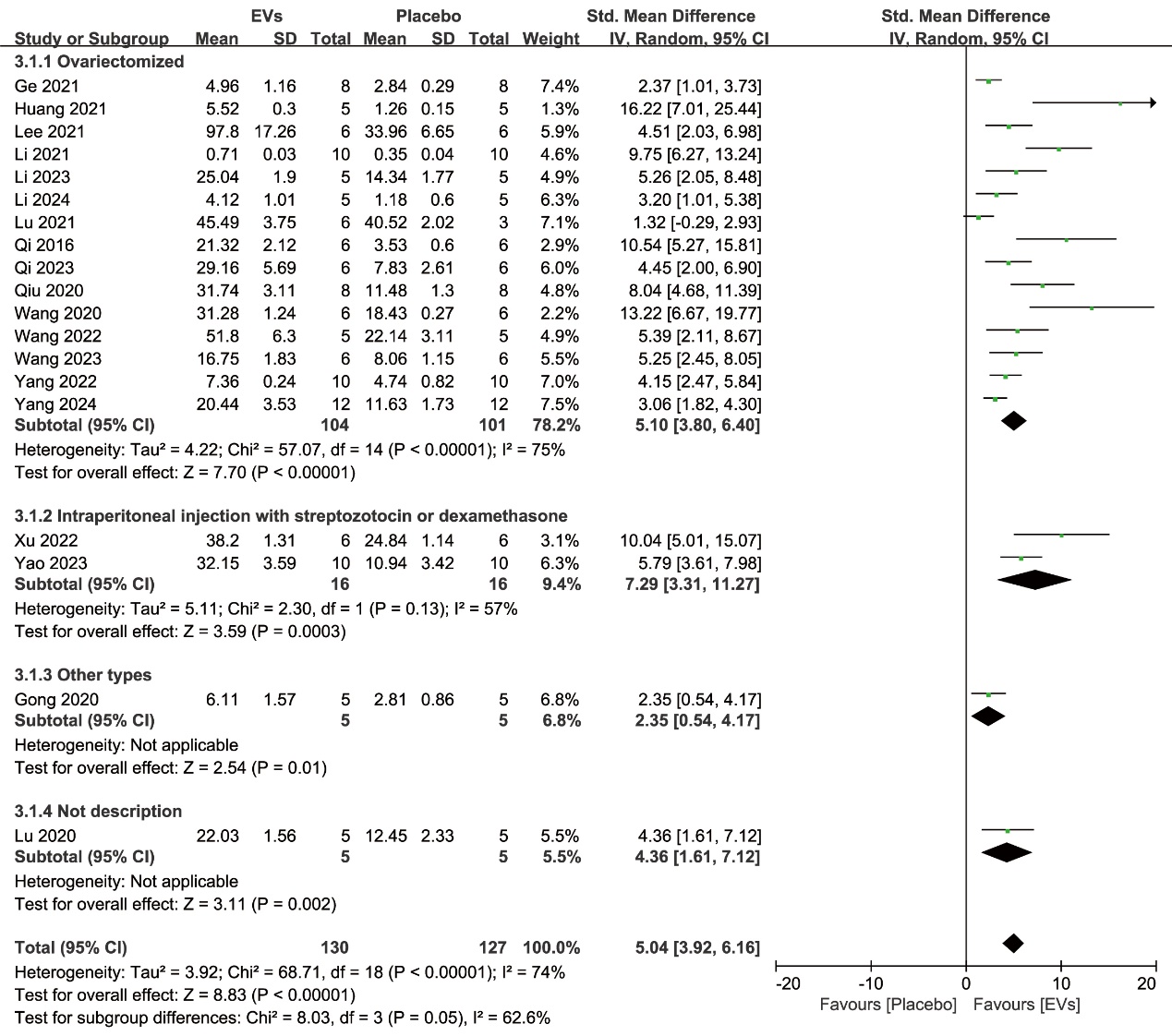
**

**Figure S46.** Subgroup analysis of BV/TV based on different modeling method. Data are presented as standardized mean difference (SMD) with 95% confidence intervals (CI).


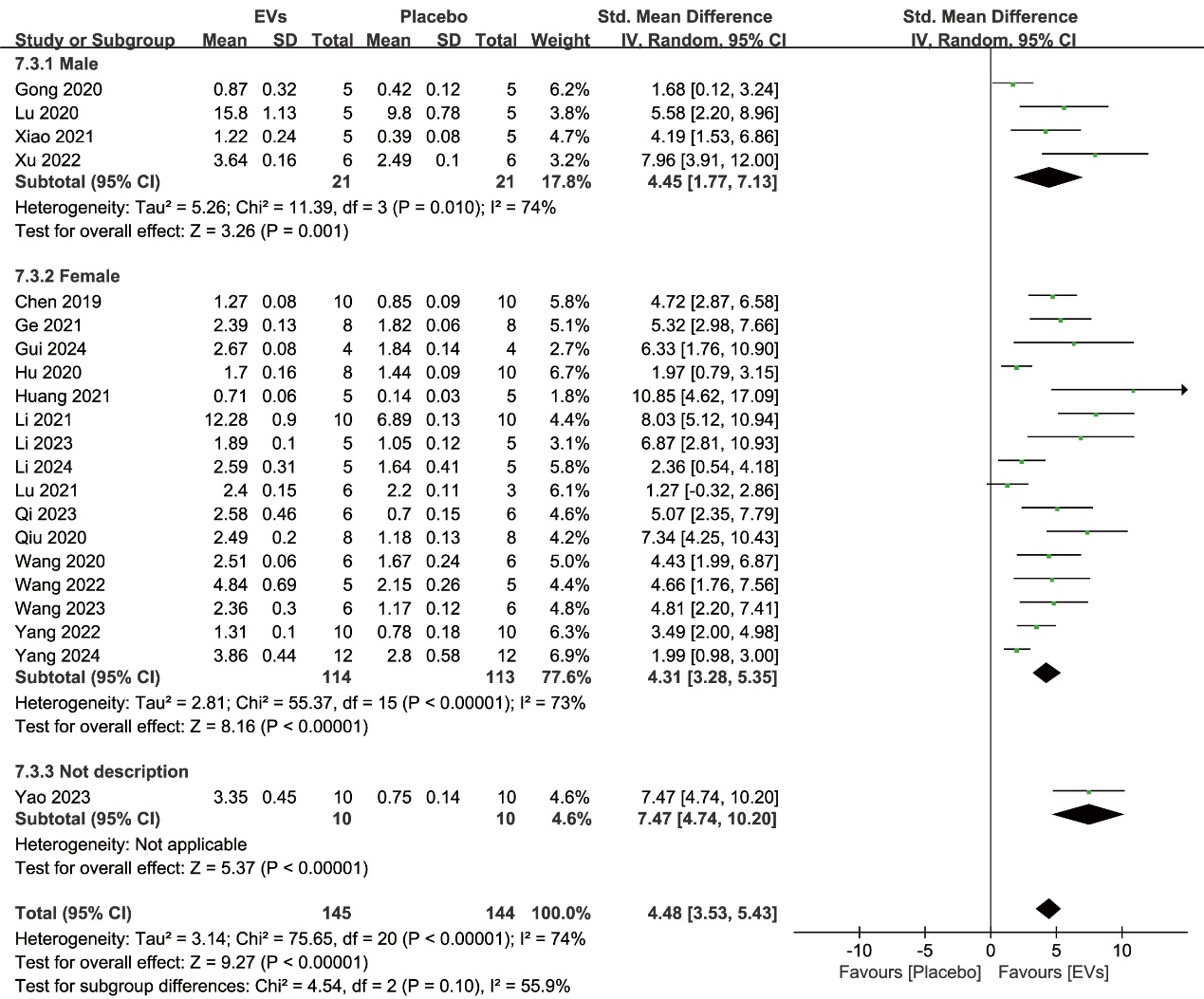


**Figure S47.** Subgroup analysis of Tb. N based on different animal sexes. Data are presented as standardized mean difference (SMD) with 95% confidence intervals (CI).


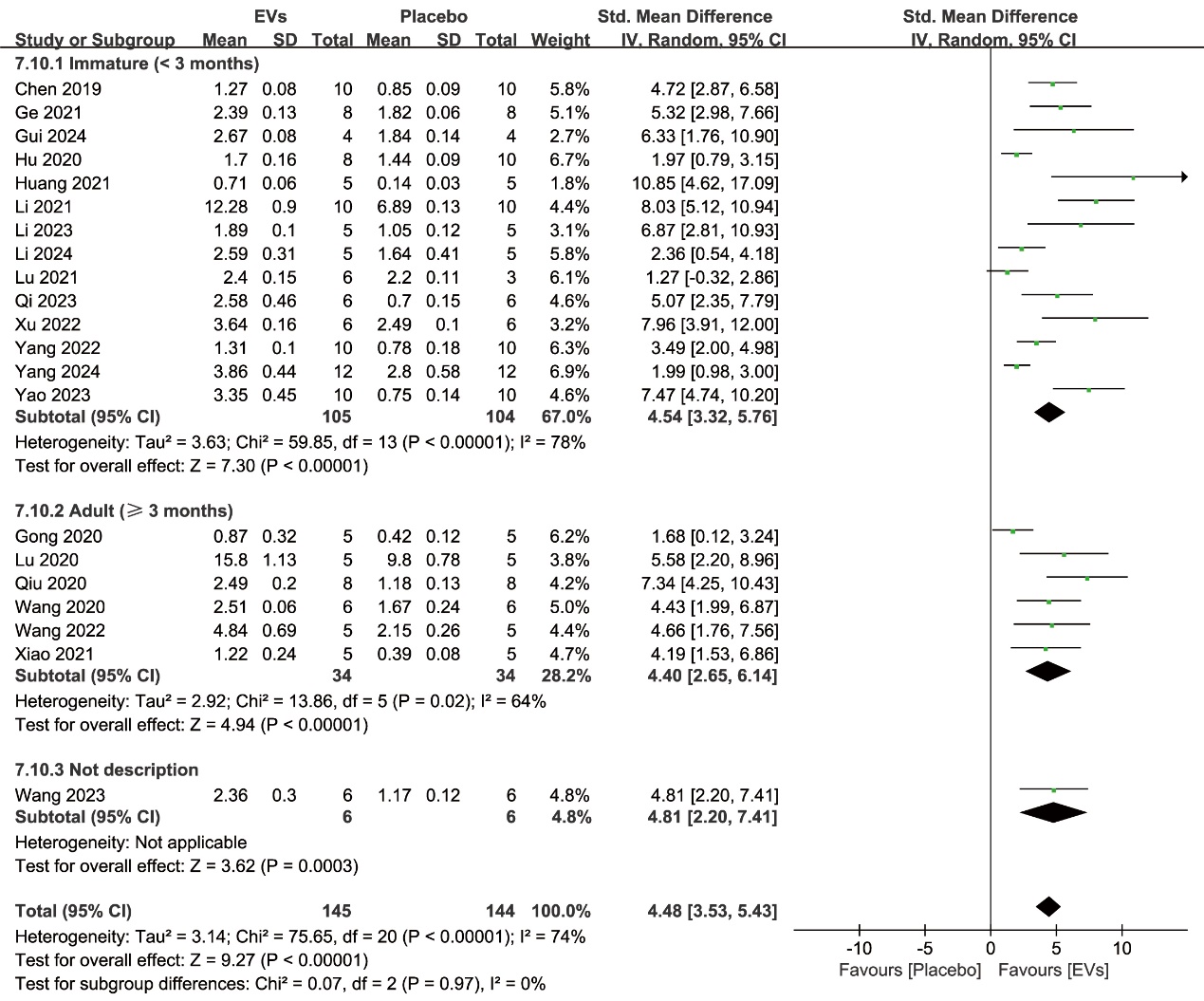


**Figure S48.** Subgroup analysis of Tb. N based on different animal ages. Data are presented as standardized mean difference (SMD) with 95% confidence intervals (CI).


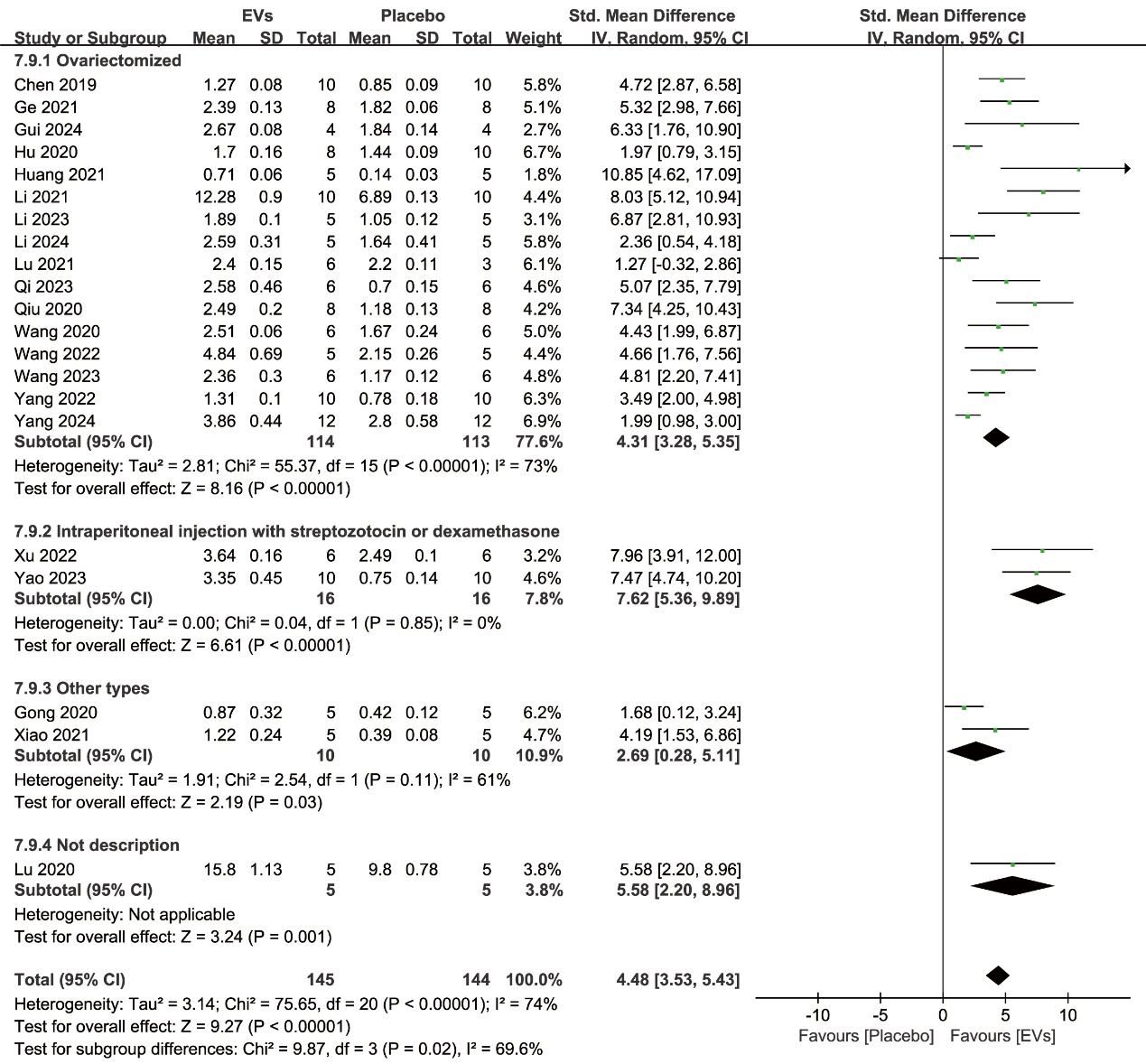


**Figure S49.** Subgroup analysis of Tb. N based on different modeling method. Data are presented as standardized mean difference (SMD) with 95% confidence intervals (CI).


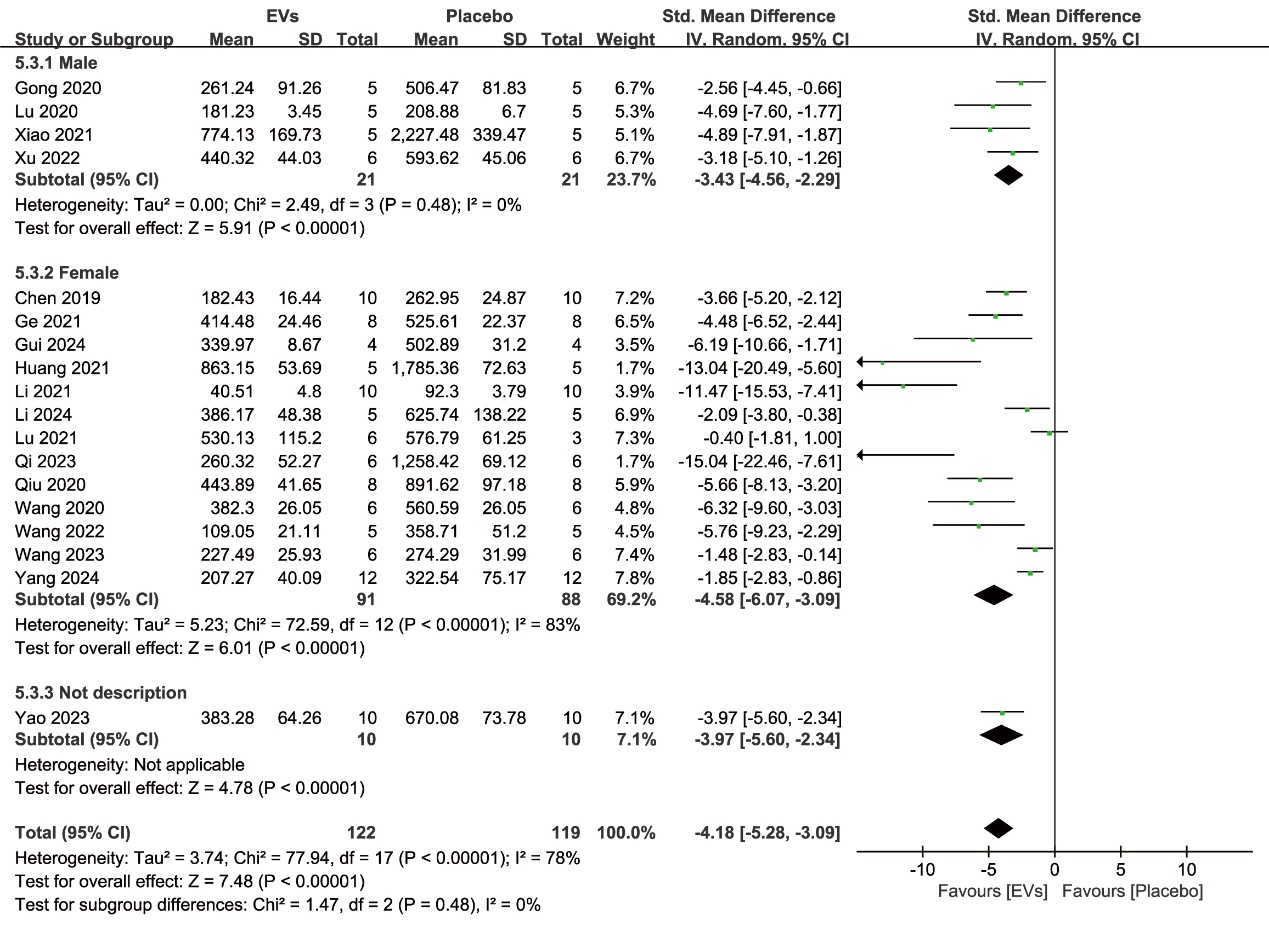


**Figure S50.** Subgroup analysis of Tb. Sp based on different animal sexes. Data are presented as standardized mean difference (SMD) with 95% confidence intervals (CI).


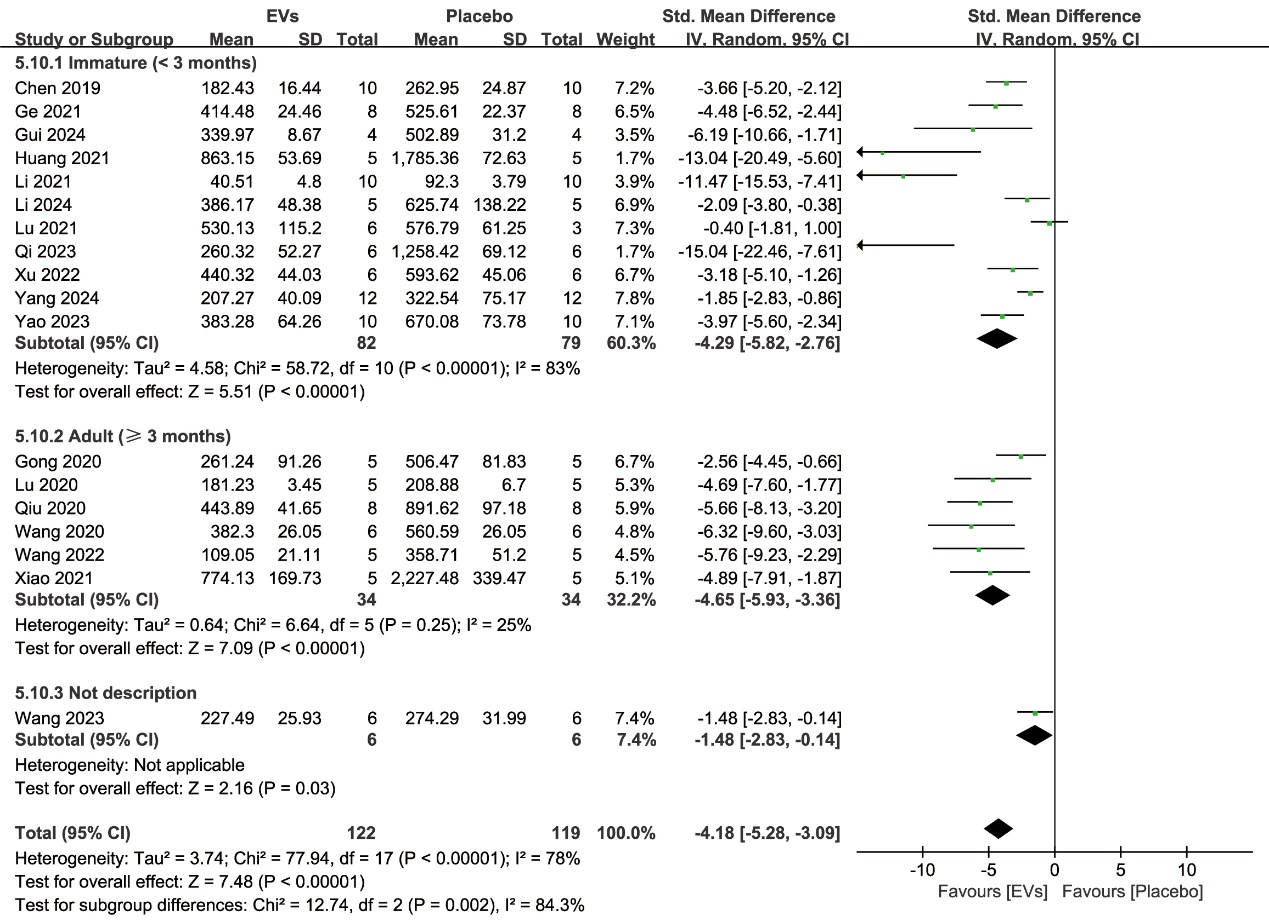


**Figure S51.** Subgroup analysis of Tb. Sp based on different animal ages. Data are presented as standardized mean difference (SMD) with 95% confidence intervals (CI).


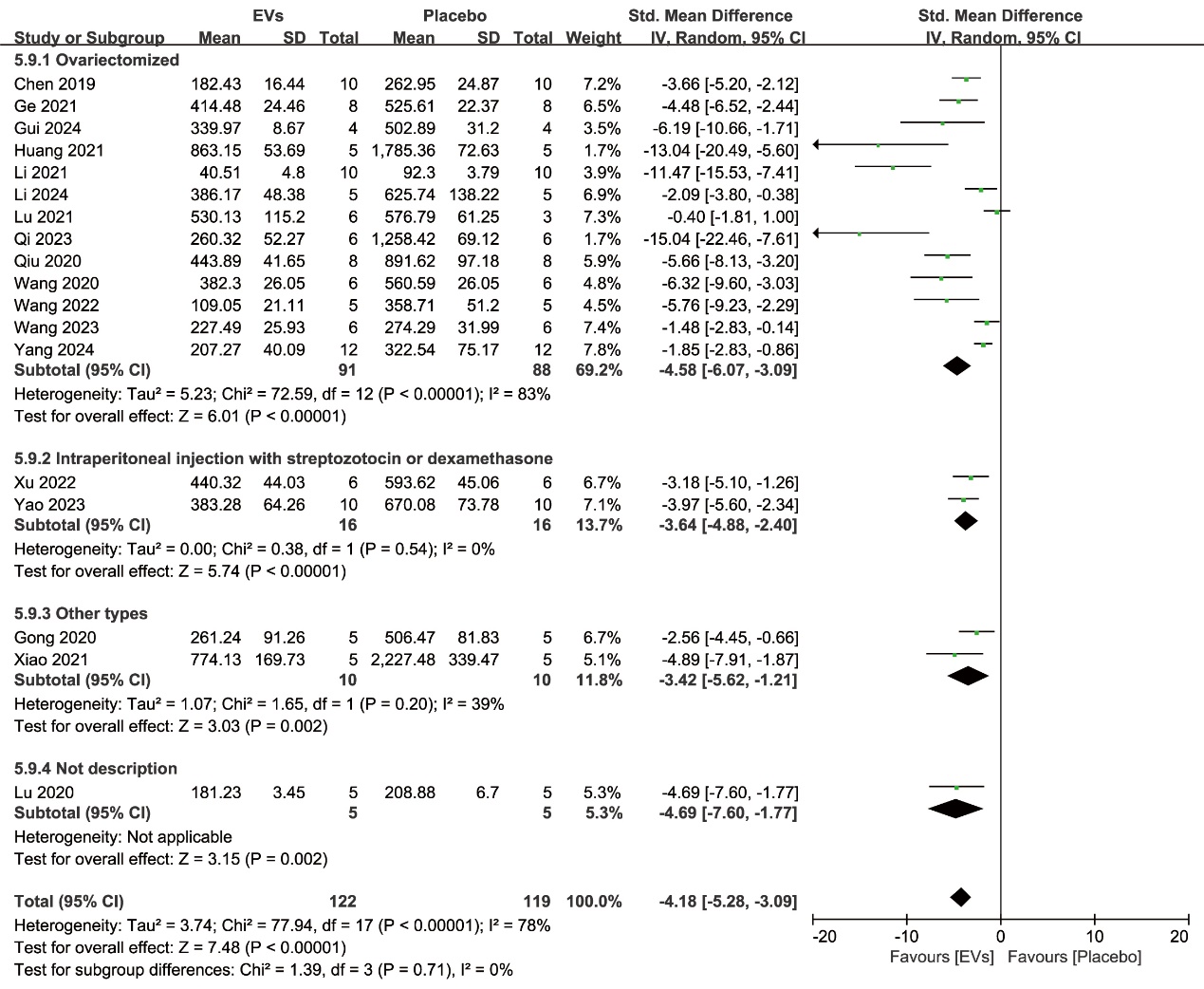


**Figure S52.** Subgroup analysis of Tb. Sp based on different modeling method. Data are presented as standardized mean difference (SMD) with 95% confidence intervals (CI).


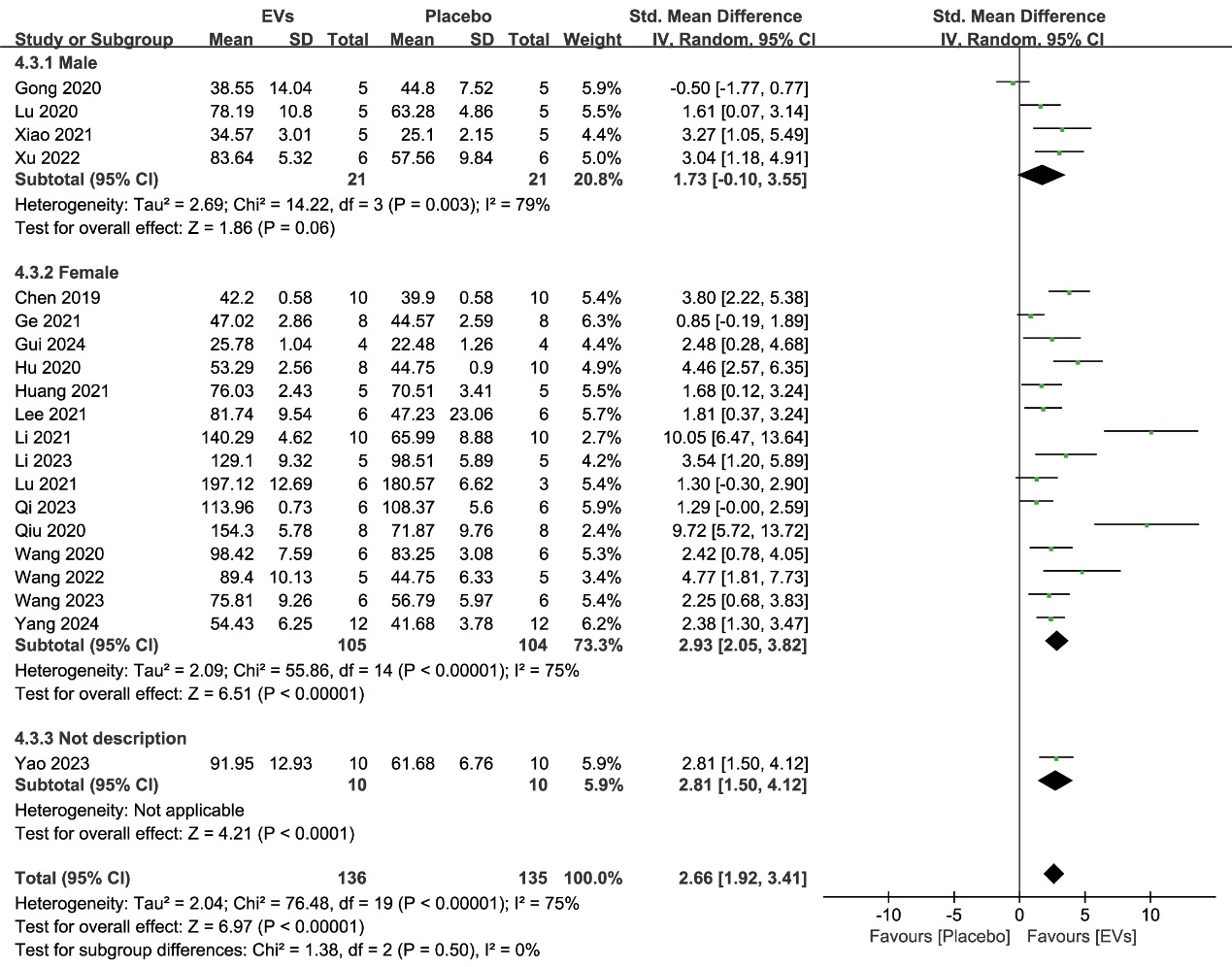


**Figure S53.** Subgroup analysis of Tb. Th based on different animal sexes. Data are presented as standardized mean difference (SMD) with 95% confidence intervals (CI).


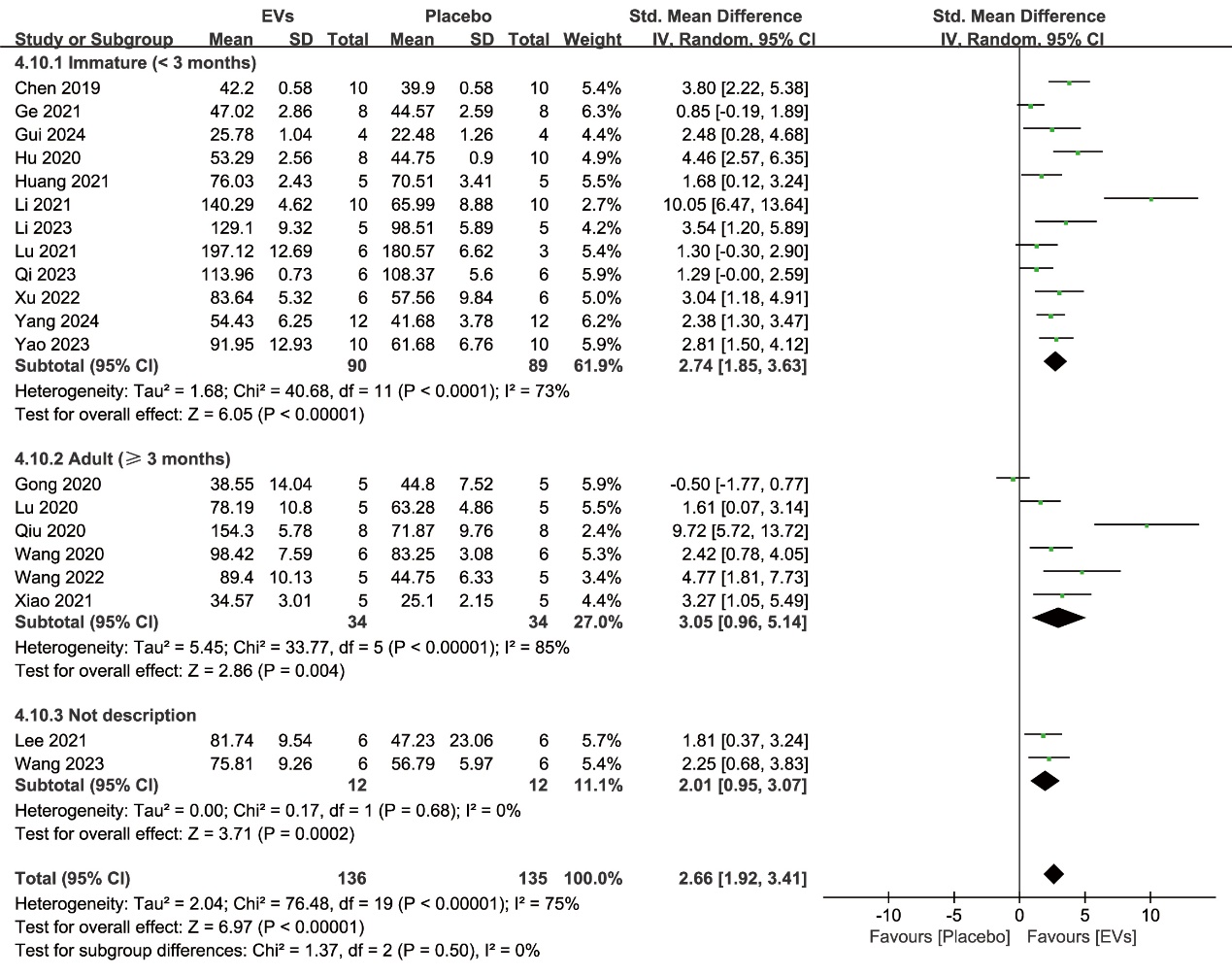


**Figure S54.** Subgroup analysis of Tb. Th based on different animal ages. Data are presented as standardized mean difference (SMD) with 95% confidence intervals (CI).


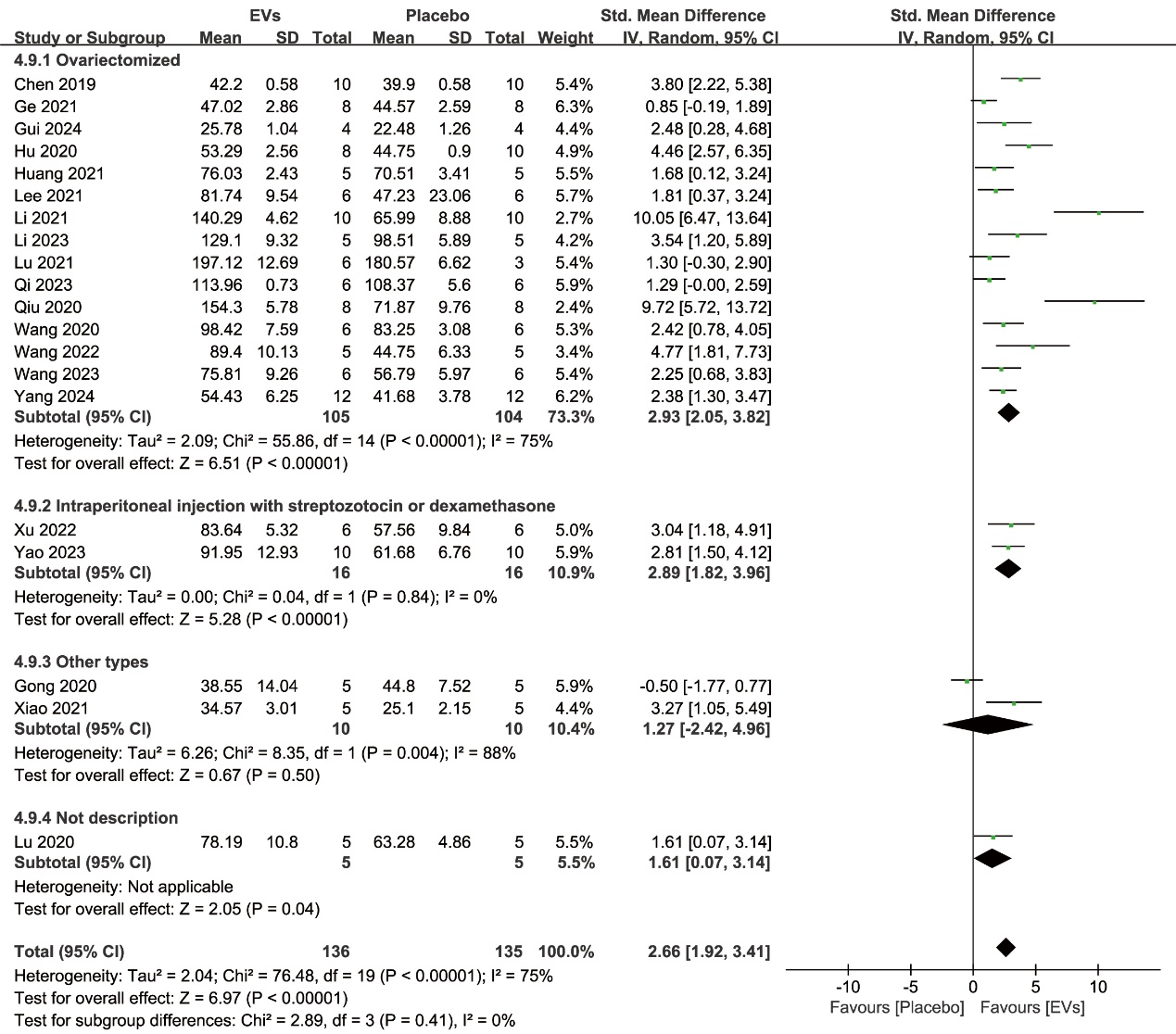


**Figure S55.** Subgroup analysis of Tb. Th based on different modeling method. Data are presented as standardized mean difference (SMD) with 95% confidence intervals (CI).


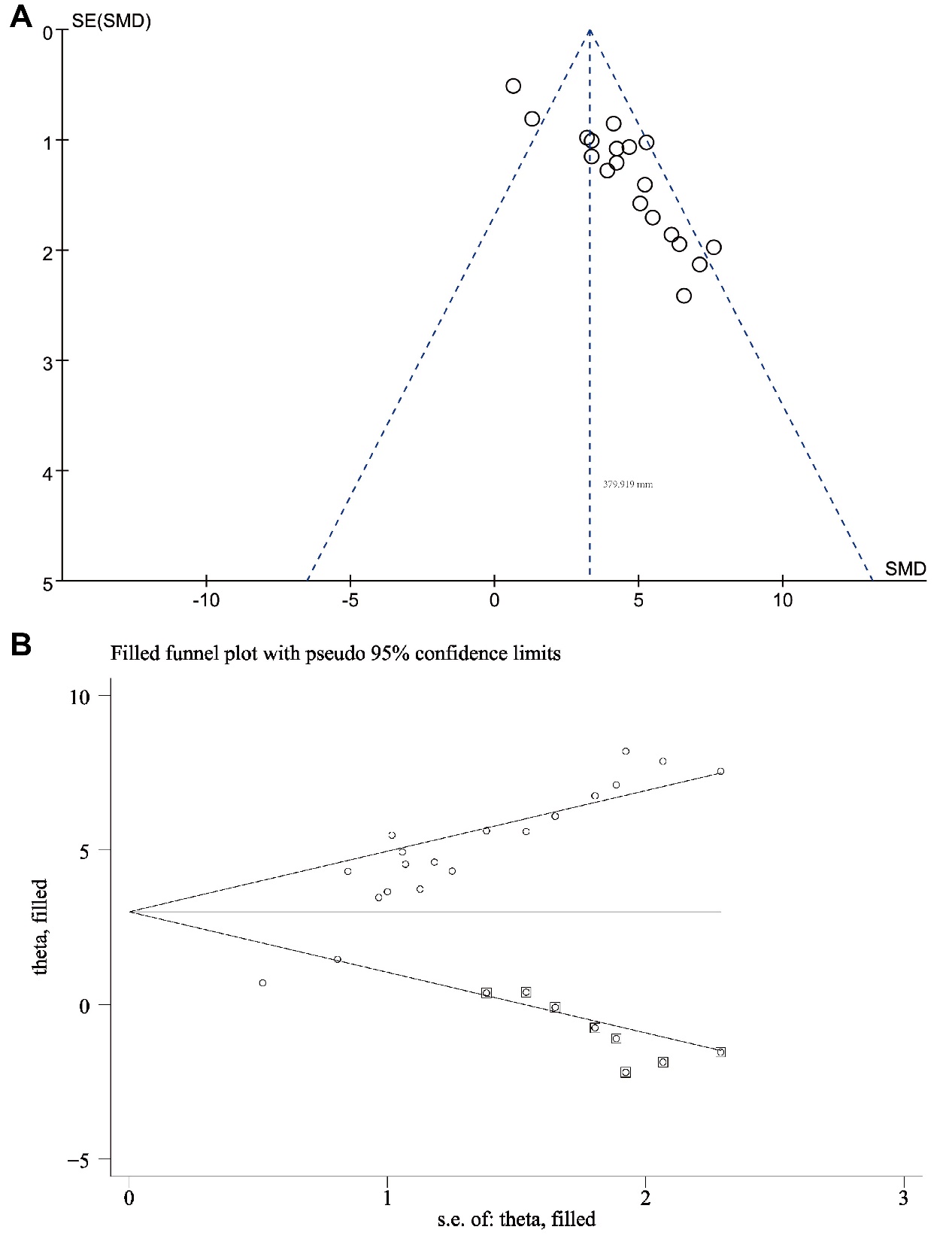


**Figure S56.** Assessment of publication bias for bone mineral density (BMD). (A) Funnel plot evaluating publication bias; (B) Filled funnel plot with pseudo-95% confidence limits.


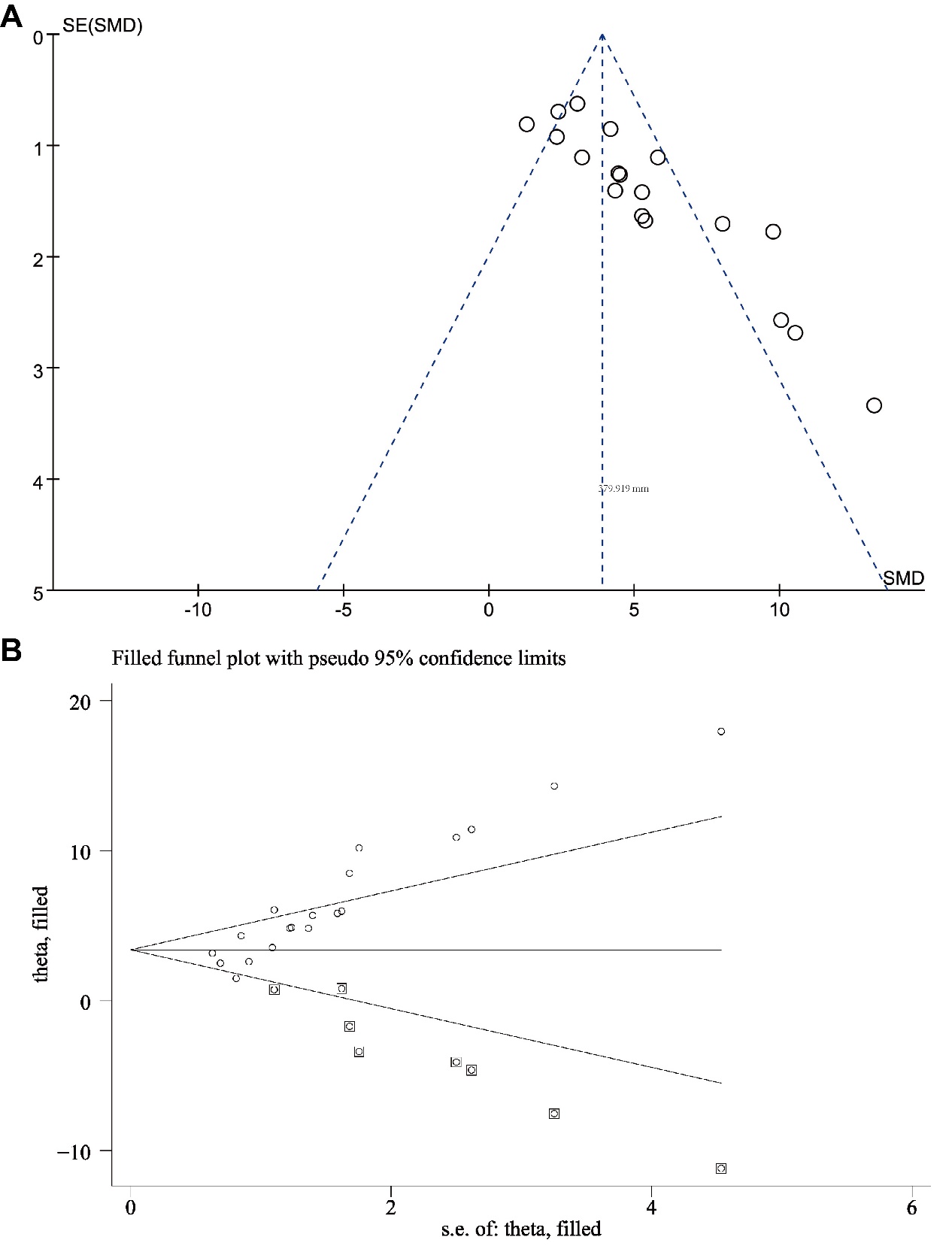


**Figure S57.** Assessment of publication bias for BV/TV. (A) Funnel plot evaluating publication bias; (B) Filled funnel plot with pseudo-95% confidence limits.


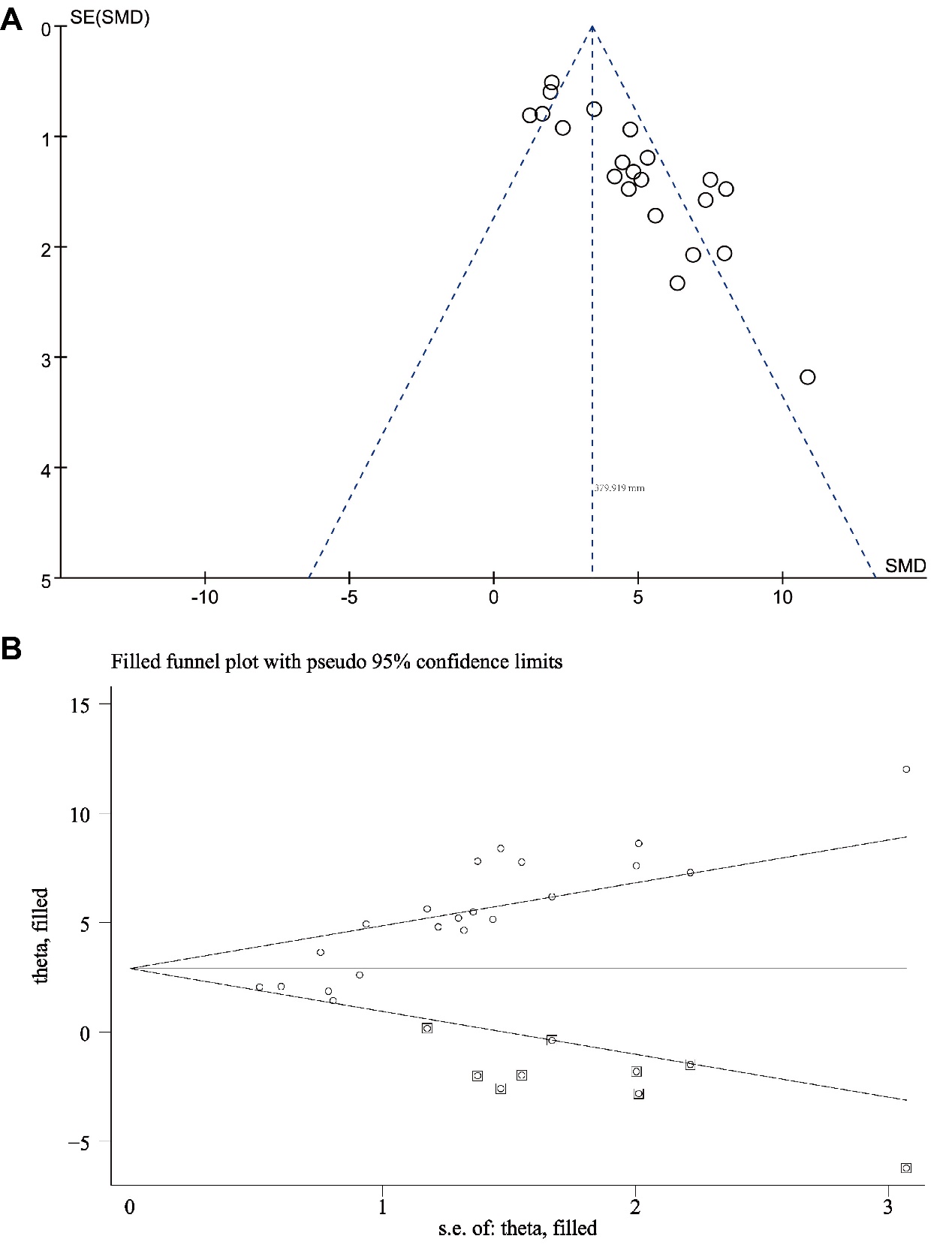


**Figure S58.** Assessment of publication bias for Tb. N. (A) Funnel plot evaluating publication bias; (B) Filled funnel plot with pseudo-95% confidence limits.


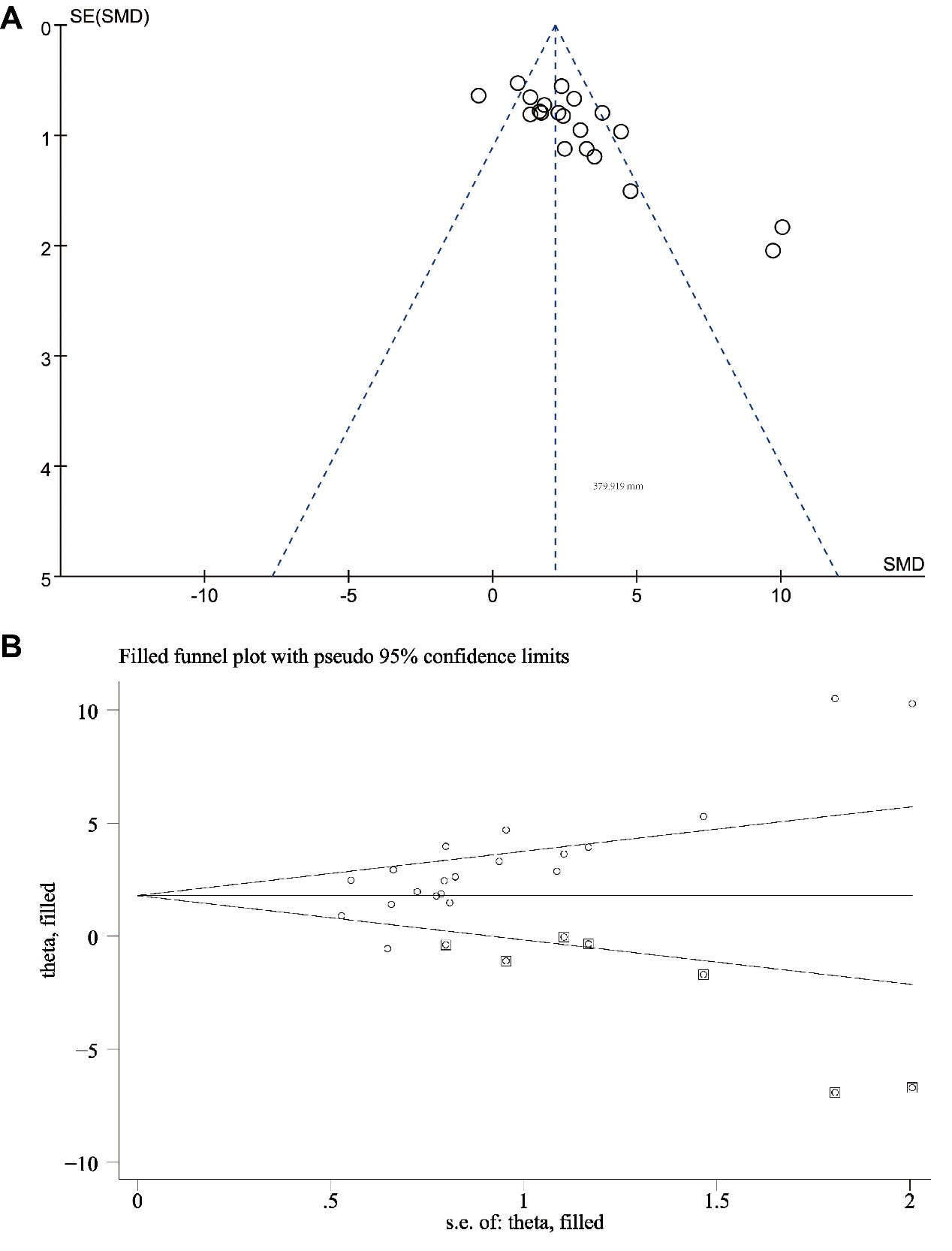


**Figure S59.** Assessment of publication bias for Tb. Th. (A) Funnel plot evaluating publication bias; (B) Filled funnel plot with pseudo-95% confidence limits.


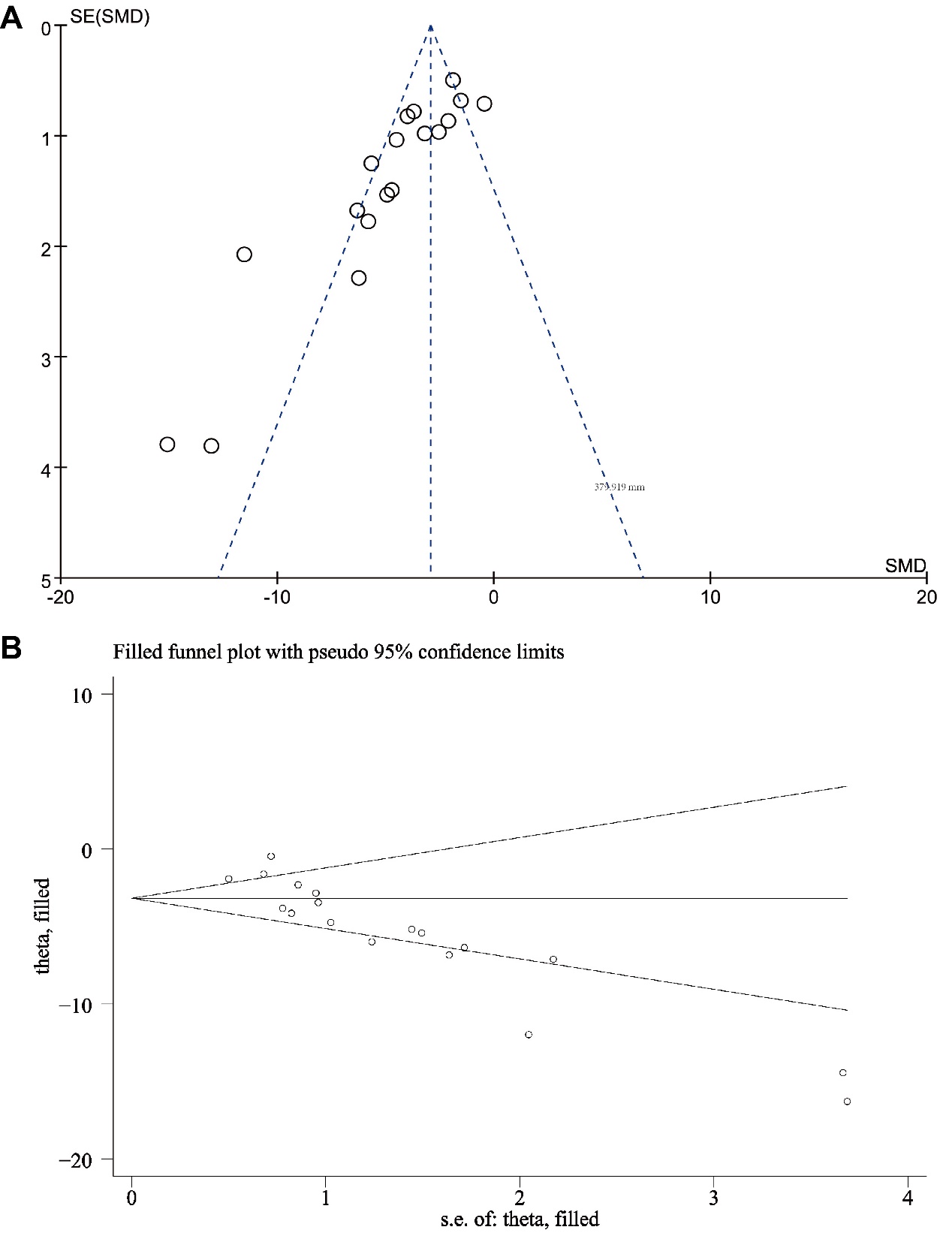


**Figure S60.** Assessment of publication bias for Tb. Sp. (A) Funnel plot evaluating publication bias for Tb. Sp; (B) Filled funnel plot with pseudo-95% confidence limits.

| **Table S1**. Publication bias analysis. | | | |
| --- | --- | --- | --- |
| Outcome | Egger's test (*p* value) | *t* value | Pooling model |
| BMD | 0.000 | 8.78 | Random |
| BV/TV | 0.000 | 8.50 | Random |
| Tb. N | 0.000 | 8.44 | Random |
| Tb. Sp | 0.000 | -7.44 | Random |
| Tb. Th | 0.000 | 5.38 | Random |

| **Table S2**. Results of the trim-and-fill method for BMD. | | | | | | |
| --- | --- | --- | --- | --- | --- | --- |
| **Method** | **Pooled Est** | **95% CI** | | **Asymptotic** | | **Number of studies** |
|  |  | **Lower** | **Upper** | **z value** | ***p* value** |  |
| Fixed | 20.132 | 12.813 | 31.633 | 13.022 | 0.000 | 27 |
| Random | 28.029 | 10.222 | 76.859 | 6.477 | 0.000 |  |
| Test for heterogeneity: Q = 113.015 on 26 degrees of freedom (*p* = 0.000). Moment-based estimate of between studies variance = 4.997. | | | | | | |

| **Table S3**. Results of the trim-and-fill method for BV/TV. | | | | | | |
| --- | --- | --- | --- | --- | --- | --- |
| **Method** | **Pooled Est** | **95% CI** | | **Asymptotic** | | **Number of studies** |
|  |  | **Lower** | **Upper** | **z value** | ***p* value** |  |
| Fixed | 29.722 | 18.486 | 47.789 | 13.999 | 0.000 | 27 |
| Random | 38.089 | 10.370 | 139.907 | 5.483 | 0.000 |  |
| Test for heterogeneity: Q = 163.292 on 26 degrees of freedom (*p* = 0.000). Moment-based estimate of between studies variance = 8.691. | | | | | | |

| **Table S4**. Results of the trim-and-fill method for Tb. N. | | | | | | |
| --- | --- | --- | --- | --- | --- | --- |
| **Method** | **Pooled Est** | **95% CI** | | **Asymptotic** | | **Number of studies** |
|  |  | **Lower** | **Upper** | **z value** | ***p* value** |  |
| Fixed | 18.134 | 12.085 | 27.210 | 13.995 | 0.000 | 30 |
| Random | 24.648 | 8.486 | 71.591 | 5.891 | 0.000 |  |
| Test for heterogeneity: Q = 173.238 on 29 degrees of freedom (*p* = 0.000). Moment-based estimate of between studies variance = 6.644. | | | | | | |

| **Table S5**. Results of the trim-and-fill method for Tb. Th. | | | | | | |
| --- | --- | --- | --- | --- | --- | --- |
| **Method** | **Pooled Est** | **95% CI** | | **Asymptotic** | | **Number of studies** |
|  |  | **Lower** | **Upper** | **z value** | ***p* value** |  |
| Fixed | 5.974 | 4.312 | 8.278 | 10.741 | 0.000 | 27 |
| Random | 6.456 | 2.734 | 15.246 | 4.254 | 0.000 |  |
| Test for heterogeneity: Q = 165.593 on 26 degrees of freedom (*p* = 0.000). Moment-based estimate of between studies variance = 4.080. | | | | | | |
